# Regulation of PKR-dependent RNA translation inhibition by TRIM21 upon virus infection or other stress

**DOI:** 10.1371/journal.ppat.1011443

**Published:** 2023-06-16

**Authors:** Huiyi Li, Shun Liu, Qing Feng, Rilin Deng, Jingjing Wang, Xintao Wang, Renyun Tian, Yan Xu, Shengwen Chen, Qian Liu, Luoling Wang, Xinran Li, Mengyu Wan, Yousong Peng, Songqing Tang, Binbin Xue, Haizhen Zhu

**Affiliations:** 1 Institute of Pathogen Biology and Immunology of College of Biology, Hunan Provincial Key Laboratory of Medical Virology, State Key Laboratory of Chemo/Biosensing and Chemometrics, Hunan University, Changsha, Hunan, China; 2 Key Laboratory of Tropical Translational Medicine of Ministry of Education, Department of Pathogen Biology and Immunology, Institute of Pathogen Biology and Immunology, School of Basic Medicine and Life Science, The University of Hong Kong Joint Laboratory of Tropical Infectious Diseases, The First Affiliated Hospital and The Second Affiliated Hospital of Hainan Medical University, Hainan Medical University, Hainan, China; The Ohio State University, UNITED STATES

## Abstract

The host always employs various ways to defend against viral infection and spread. However, viruses have evolved their own effective strategies, such as inhibition of RNA translation of the antiviral effectors, to destroy the host’s defense barriers. Protein synthesis, commonly controlled by the α-subunit of eukaryotic translation initiation factor 2 (eIF2α), is a basic cellular biological process among all species. In response to viral infection, in addition to inducing the transcription of antiviral cytokines by innate immunity, infected cells also inhibit the RNA translation of antiviral factors by activating the protein kinase R (PKR)-eIF2α signaling pathway. Regulation of innate immunity has been well studied; however, regulation of the PKR-eIF2α signaling pathway remains unclear. In this study, we found that the E3 ligase TRIM21 negatively regulates the PKR-eIF2α signaling pathway. Mechanistically, TRIM21 interacts with the PKR phosphatase PP1α and promotes K6-linked polyubiquitination of PP1α. Ubiquitinated PP1α augments its interaction with PKR, causing PKR dephosphorylation and subsequent translational inhibition release. Furthermore, TRIM21 can constitutively restrict viral infection by reversing PKR-dependent translational inhibition of various previously known and unknown antiviral factors. Our study highlights a previously undiscovered role of TRIM21 in regulating translation, which will provide new insights into the host antiviral response and novel targets for the treatment of translation-associated diseases in the clinic.

## Author summary

Transcriptional induction of antiviral cytokines, especially interferons (IFNs), is a hallmark of innate immunity upon viral infection. In addition, viral infection can inhibit RNA translation of the antiviral factors by activating the PKR-eIF2α axis. Regulation of the innate immune signaling pathway has been well studied. However, regulation of the PKR signaling pathway remains unclear. Our previous study demonstrated that TRIM21, an E3 ligase, positively regulates the innate immune response by catalyzing K27-linked ubiquitination of MAVS. However, whether TRIM21 regulates the PKR signaling pathway is unknown.

In this study, we found that TRIM21 can negatively regulate PKR activation by promoting K6-linked ubiquitination of the PKR phosphatase PP1α, contributing to the enhancement of the PKR-PP1α interaction, which leads to dephosphorylation of PKR. Moreover, our study identified various previously known and unknown antiviral factors regulated by the TRIM21-PKR axis. Our study highlights TRIM21-mediated PKR-dependent RNA translation in the antiviral response, which may provide a target for the treatment of translation-associated diseases in the clinic.

## Introduction

To survive and produce viral particles, viruses employ various strategies, such as shutdown of the protein synthesis of antiviral factors, to escape immune supervision by host cells. Protein synthesis is a basic biological process among all species, including three steps: initiation, elongation and termination. Appropriate regulation of RNA translation is essential for the maintenance of protein homeostasis, and disruption of RNA translation by extrinsic or intrinsic stresses is extremely prone to causing severe organ damage, which contributes to tumorigenesis and inflammation [1]. In response to extracellular or intracellular alterations, eukaryotic cells commonly employ programs that conserve energy and facilitate the reprogramming of gene expression by inhibiting RNA translation to adapt to alterations in the cellular environment [2]. However, this protective mechanism of the host can be also beneficial to the survival of the virus because of the inhibition of antiviral effector RNA translation upon viral infection. The central mechanism regulating protein synthesis in cells upon stimulation involves the phosphorylation of the translation initiation factor eIF2α [3]. There are four kinases, the dsRNA-dependent protein kinase known as protein kinase R (PKR), general control nonrepressed 2 (GCN2), PKR-like endoplasmic reticulum kinase (PERK) and heme-regulated eIF2α kinase (HRI), that are responsible for eIF2α phosphorylation in eukaryotic cells [4]. Among them, as one of the pattern recognition receptors (PRRs) involved in innate immunity, PKR is distinct for its essential role in response to viral infection or inflammasome-mediated innate immunity [5].

Viral infection triggers PKR-dependent protein synthesis inhibition. PKR is a serine-threonine kinase comprised of two conserved double-stranded RNA binding motifs (dsRBMs) in its N-terminal domain and a C-terminal kinase domain. In the cytoplasm, by detecting dsRNA via its N-terminal dsRBM, PKR dimerizes and rapidly phosphorylates itself at residues Thr446 and Thr451 and then activates eIF2α by phosphorylation, resulting in protein synthesis inhibition [6,7]. Accumulated evidence have demonstrated that PKR-mediated inhibition of translation initiation plays crucial roles in human physiological and pathological processes, such as the regulation of cell proliferation, differentiation, apoptosis, viral infection, cancer and inflammation [8–11]. However, the precise regulatory mechanism remains unclear. In addition to activating the PKR-eIF2α signaling pathway, dsRNA can also trigger innate immunity activation by binding to the PRRs in the cytosol, such as retinoic acid-inducible gene I (RIG-I) and melanoma differentiation associated protein 5 (MDA5). RIG-I- or MDA5-mediated innate immune signaling accounts for the activation of the transcription factors IRF3 and NF-κB, subsequently resulting in the induction of the transcription of multiple antiviral cytokines, particularly IFNs [12,13]. It is well known that regulation of the RIG-I- or MDA5-mediated signaling pathway has been extensively studied. However, regulation of the PKR signaling pathway remains unclear.

TRIM21 is a member of the tripartite motif (TRIM) superfamily, which plays important roles in diverse biological and pathophysiological processes [14]. Our previous study showed that TRIM21, by catalyzing the K27-linked ubiquitination of mitochondrial antiviral signaling protein (MAVS), augments RIG-I/MDA5-mediated transcription of IFNs upon viral infection [15]. However, whether TRIM21 is able to restrict viral infection via regulation of the protein synthesis of antiviral effectors remains elusive. In this study, we found that TRIM21 negatively regulates PKR-dependent translational shutdown upon viral infection or thapsigargin (TG) treatment. Knockout or knockdown of *TRIM21* augments virus- or TG-induced PKR phosphorylation. Mechanistically, viral infection or TG treatment promotes TRIM21 binding to the PKR phosphatase PP1α, which leads to the K6-linked polyubiquitination of PP1α. Ubiquitinated PP1α augments its interaction with PKR, resulting in dephosphorylation of PKR and subsequent eIF2α inactivation, resulting in the release of protein synthesis inhibition. Moreover, in addition to its antiviral function through promotion of the transcription of IFNs, proteomics analysis revealed that TRIM21 is able to restrict viral infection by reversing PKR-mediated inhibition of the protein synthesis of previously known and unknown intrinsic antiviral genes in cells. Collectively, our data highlight the essential role of TRIM21 in regulating RNA translation, which may provide new strategies for the treatment of translation initiation-associated diseases.

## Results

### TRIM21 inhibits the activation of PKR and promotes RNA translation

**Fig 1:**
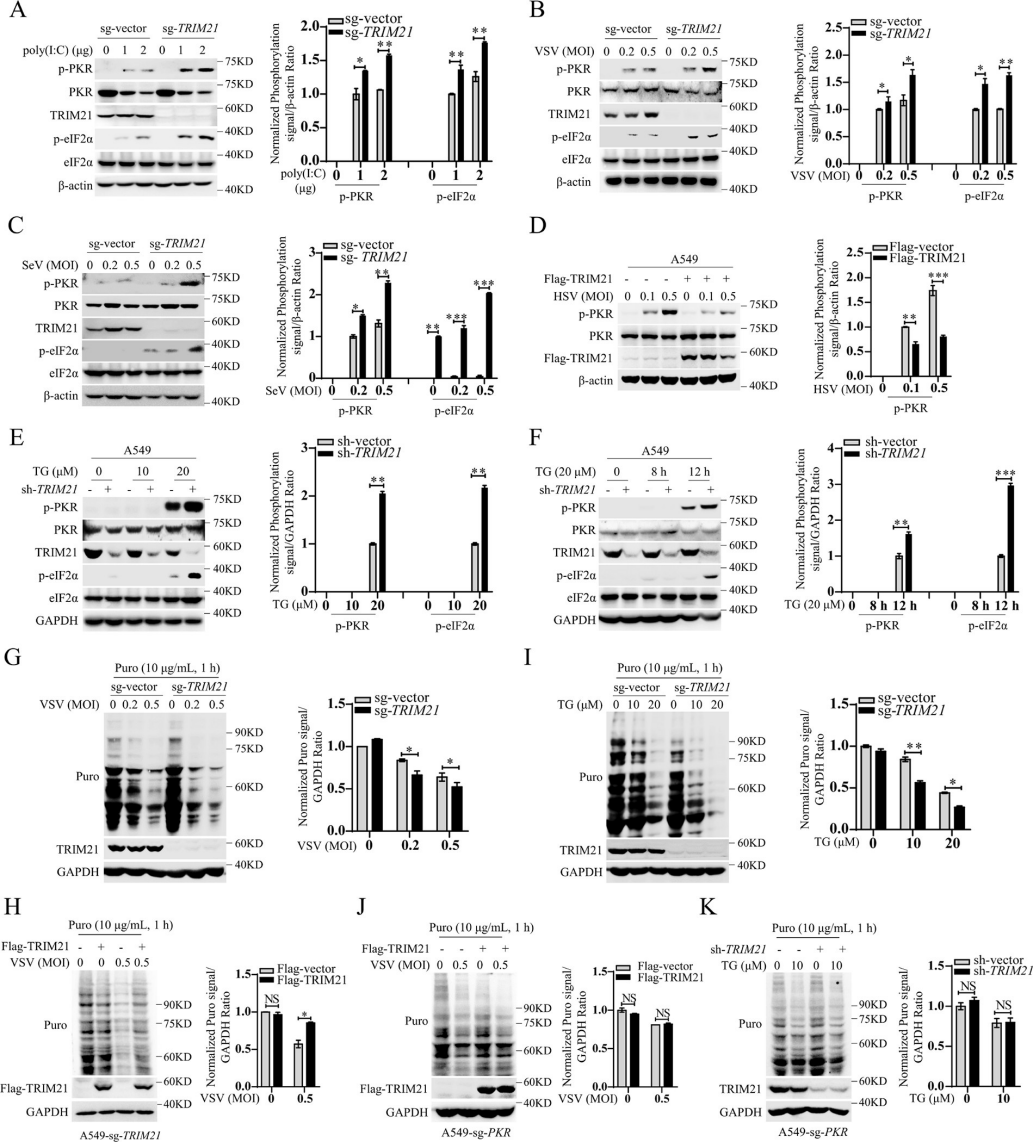
TRIM21 inhibits the activation of PKR. (**A-C**) Wild-type (sg-vector) or *TRIM21*-knocked out (sg-*TRIM21*) A549 cells were transfected with poly(I:C) for 12 h (A), or infected with VSV (MOI = 0.2 or 0.5) for 6 h (B) or infected with SeV (MOI = 0.2 or 0.5) for 12 h (C). The indicated proteins were detected by western blot. β-actin was used as an internal control. (**D**) A549 cells were transfected with p3xFlag-CMV-vector or p3xFlag-CMV-TRIM21 for 48 h, then infected with HSV (MOI = 0.1 or 0.5) for 6 h. The indicated proteins were detected by western blot. β-actin was used as an internal control. (**E-F**) A549 cells were infected with lentivirus-sh-vector (sh-vector) or lentivirus-sh-*TRIM21* (sh-*TRIM21*) for 48 h, then stimulated by thapsigargin (TG) (10 μM or 20 μM) for 12 h (E), or TG (20 μM) for 8 h or 12 h (F). The indicated proteins were detected by western blot. GAPDH was used as a control. (**G**) Puromycin incorporation assays demonstrated the cellular protein synthesis in mock- or VSV-infected wild-type A549 cells (sg-vector) or *TRIM21*-deficient A549 cells (sg-*TRIM21*). The cells were pretreated with puromycin (10 μg/mL) for 1 h. The indicated proteins were detected by western blot. GAPDH was used as a control. (**H**) Puromycin incorporation assays of the cellular protein synthesis in *TRIM21*-deficient A549 cells (sg-*TRIM21*) transfected with p3xFlag-CMV-vector or p3xFlag-CMV-TRIM21 for 48 h, then infected by VSV (MOI = 0.5) for 6 h. GAPDH was used as an internal control. (**I**) Immunoblot analysis of puromycin and TRIM21 in wild-type A549 cells (sg-vector) or *TRIM21*-deficient A549 cells (sg-*TRIM21*) stimulated with TG (10 μM or 20 μM) for 12 h. GAPDH was used as an internal control. (**J-K**) Puromycin incorporation assays of the cellular protein synthesis in *PKR*-deficient A549 cells (sg-*PKR*) transfected with p3xFlag-CMV-vector or p3xFlag-CMV-TRIM21 for 48 h, then infected by VSV (MOI = 0.5) for 6 h (J), or infected with lentivirus-sh-vector (sh-vector) or lentivirus-sh-*TRIM21* (sh-*TRIM21*) for 48 h, then stimulated by TG (10 μM) for 12 h (K), GAPDH was used as an internal control. The relative ratios of p-PKR or p-eIF2α in **(A-F)** and the relative ratios of the puro signals in **(G-K)** were quantified by densiometric analysis, which were normalized to the value in the control group. Experiments were independently repeated two or three times with similar results, and the data shown are mean ± SD. *P* values were determined by Student’s *t*-test. *p<0.05, **p<0.01, ***p<0.001, NS, no significance difference.

To ascertain whether TRIM21 affects PKR signaling pathway activation, we used CRISPR–Cas9 gene editing technology to establish *TRIM21*-deficient A549 cell lines (sg-*TRIM21*) (S1A Fig). We assessed the effect of TRIM21 in the regulation of the PKR signaling pathway by employing a classic agonist of PKR, poly (I:C), in these cells. By transfection of poly (I:C), the phosphorylation levels of PKR and eIF2α in *TRIM21*-deficient cells were higher than those in wild-type cells, suggesting a negative regulatory role of TRIM21 in PKR signaling pathway activation (Fig 1A). To rule out the possibility of an off-target effect of *TRIM21* sgRNA, we used shRNA specifically targeting *TRIM21* to knockdown the expression of TRIM21 in A549 cells (S1B Fig). Similarly, knockdown of *TRIM21* enhanced poly (I:C)-induced phosphorylation of PKR and eIF2α (S1C Fig). Next, we examined the effects of TRIM21 on PKR signaling activation upon viral infection. In line with previous studies, vesicular stomatitis virus (VSV) or Sendai virus (SeV) infection constitutively activated PKR and eIF2α, and *TRIM21* deficiency enhanced their activation (Fig 1B and 1C). Moreover, we obtained similar results in *TRIM21*-silenced cells upon VSV or SeV infection (S1D and S1E Fig). To exclude the specificity of the cell lines, we performed the above experiments in HLCZ01 cells, a liver cell line, which supports the entire life cycle of hepatitis B virus (HBV) and hepatitis C virus (HCV) [16]. Consistently, the levels of p-PKR and p-eIF2α were also increased in HLCZ01 cells with *TRIM21* knockdown upon poly (I:C) treatment or viral infection (S1F–S1H Fig), demonstrating that TRIM21 can repress dsRNA- or virus-induced PKR signaling pathway activation. To further investigate the negative regulatory role of TRIM21 in PKR signaling pathway activation, we used different stimuli. Because dsRNAs produced during viral infection can activate PKR [17], we tried to test the function of TRIM21 during infection with a DNA virus, herpes simplex virus-1 (HSV-1). Notably, HSV-1 infection substantially activated PKR, while this activation was inhibited by TRIM21 overexpression in the cells (Fig 1D), suggesting that TRIM21 inhibits DNA virus-induced PKR signaling pathway activation. Moreover, TRIM21 also repressed another PKR agonist, ER stress activator thapsigargin (TG)-induced PKR activation (Fig 1E and 1F) [18]. These data demonstrated that TRIM21 negatively regulates PKR signaling pathway activation. PKR activation commonly leads to global protein synthesis inhibition, consistent with its inhibition of PKR activation, TRIM21 reversed the global translation inhibition caused by viral infection or TG treatment (Fig 1G–1I). However, the phenomenon was lost upon PKR deficiency (Figs 1J, 1K and S1I), indicating that TRIM21 can release PKR-dependent protein synthesis shutdown. Collectively, these data demonstrated that TRIM21 can inhibit PKR signaling pathway activation and reverse PKR activation-mediated protein synthesis inhibition.

### The ubiquitin ligase activity of TRIM21 is required for TRIM21-mediated inhibition of PKR activation

**Fig 2:**
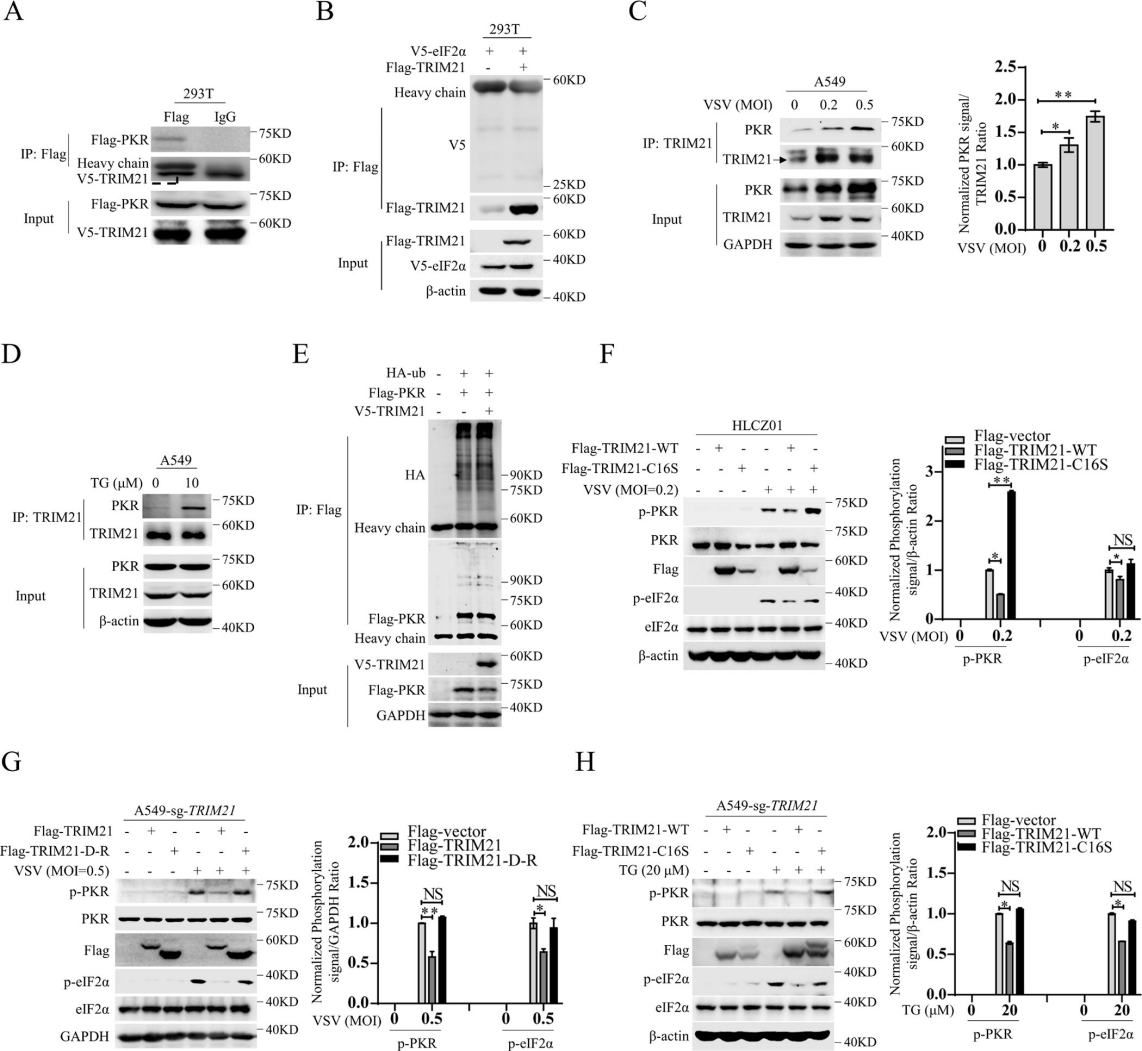
The ubiquitin ligase activity of TRIM21 is required for TRIM21-mediated inhibition of PKR activation. (**A**) Immunoprecipitation analysis the interaction between PKR and TRIM21. HEK293T cells were transfected with p3xFlag-CMV-PKR and pcDNA3.1a-TRIM21 for 48 h. The anti-Flag antibody was used to perform immunoprecipitation and detect Flag-PKR, the anti-V5 antibody was used to detect V5-TRIM21. (**B**) HEK293T cells were co-transfected with p3xFlag-CMV-TRIM21 and pcDNA3.1a-eIF2α for 48 h. The anti-Flag antibody was used to perform immunoprecipitation and detect Flag-PKR. The anti-V5 antibody was used to detect V5-eIF2α. β-actin was used as an internal control. (**C-D**) Endogenous PKR and TRIM21 interaction was analyzed by IP in A549 cells with VSV (MOI = 0.2 or 0.5) infection for 6 h (C) or with TG (10 μM) treatment for 12 h (D). The anti-TRIM21 antibody was used to perform immunoprecipitation. The indicated proteins were detected by the corresponding antibodies. GAPDH (C) or β-actin (D) was used as an internal control. (**E**) Immunoprecipitation analysis of the ubiquitination of PKR. HEK293T cells were co-transfected p3xFlag-CMV-PKR with pcDNA3.1a-vector or pcDNA3.1a-TRIM21 as well as HA-ub for 48 h. The anti-Flag antibody was used to perform immunoprecipitation and detect Flag-PKR. The anti-V5 antibody was used to detect V5-TRIM21. GAPDH was used as an internal control. (**F**) HLCZ01 cells were infected with lentivirus-Flag-TRIM21-WT or lentivirus-Flag-TRIM21-C16S for 48 h, then infected with VSV (MOI = 0.2) for 6 h. The indicated proteins were detected by western blot. β-actin was used as an internal control. (**G**) *TRIM21*-deficient A549 cells (A549-sg-*TRIM21*) were transfected with p3xFlag-CMV-TRIM21 or p3xFlag-CMV-TRIM21-D-R for 48h, then infected with VSV (MOI = 0.5) for 6 h. The indicated proteins were detected by western blot. GAPDH was used as an internal control. (**H**) *TRIM21*-deficient A549 cells (A549-sg-*TRIM21*) were infected with lentivirus-Flag-TRIM21-WT or lentivirus-Flag-TRIM21-C16S for 48 h, then stimulated with TG (20 μM) for 12 h. The indicated proteins were detected by western blot. β-actin was used as an internal control. The relative interaction ratios of PKR and TRIM21 in **(C)** were quantified by densiometric analysis, which were normalized to the value in the control group. The relative ratios of p-PKR or p-eIF2α in (**F-H**) were quantified by densiometric analysis, which were normalized to the value in the control group. Experiments were independently repeated two or three times with similar results, and the data shown are mean ± SD. *P* values were determined by Student’s *t*-test. *p<0.05, **P<0.01, NS, no significance difference.

To explore the mechanism of TRIM21-mediated inhibition of the PKR signaling pathway, we first examined the interaction between TRIM21 and the signaling molecules in the pathway. Notably, TRIM21 interacted with PKR but not eIF2α (Fig 2A and 2B), and the endogenous interaction between them was further confirmed in A549 cells, in which their interaction was enhanced upon viral infection or TG treatment (Fig 2C and 2D), indicating that TRIM21 may inhibit PKR activation by targeting PKR. TRIM21 is an E3 ligase with enzymatic activity within its RING finger domain, which is essential for its function in multiple biological and pathological processes. To investigate whether the E3 ligase activity of TRIM21 is involved in the regulation of PKR activation, we cotransfected pFlag-tagged PKR and hemagglutinin (HA)-tagged ubiquitin (HA-ub) together with pV5-tagged TRIM21 into HEK293T cells and found that TRIM21 has no effect on PKR ubiquitination, indicating that TRIM21 does not directly ubiquitinate PKR (Fig 2E). However, the E3 ligase-inactive mutant (TRIM21-C16S), in which Cys16 was replaced with Ser16, lost the ability to reduce VSV-induced PKR phosphorylation (Fig 2F). To confirm the essential role of the E3 ligase activity in PKR inactivation, we constructed a TRIM21 mutant deleting RING domain (TRIM21-D-R), and found that the phosphorylation level of PKR in the cells expressing the full-length TRIM21 (Flag-TRIM21-FL) was lower than that in the cells transfecting Flag-vector upon VSV infection, however, the phenomenon was rescued by transfection with Flag-TRIM21-D-R (Fig 2G). Moreover, the E3 ligase activity deficiency also lost the ability to inactive PKR (Fig 2H), suggesting that the E3 ligase activity of TRIM21 is required for PKR inactivation. Collectively, all of the data demonstrated that TRIM21 inhibits PKR activation by targeting PKR and that the E3 ligase activity of TRIM21 is crucial for its inactivation.

### TRIM21 represses PKR activation by targeting PP1α

**Fig 3:**
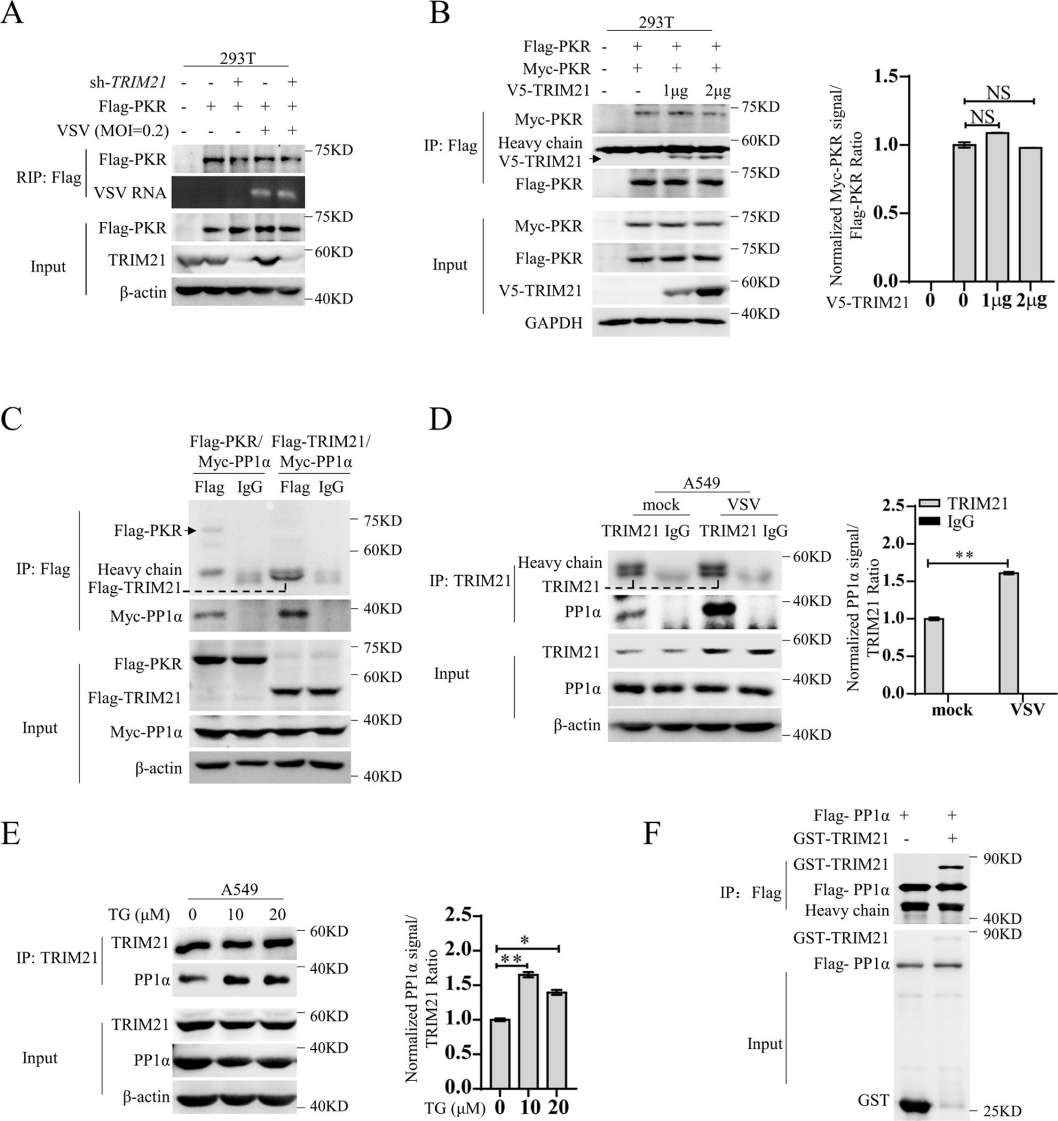
TRIM21 represses PKR activation by targeting on PP1α. (**A**) HEK293T cells pre-infected with lentivirus-sh-vector (sh-vector) or lentivirus-sh-*TRIM21* (sh-*TRIM21*) for 24 h were transfected with p3xFlag-CMV-PKR for additional 48 h, then infected with VSV (MOI = 0.2) for 6 h. RIP assay was performed to test the VSV RNA binding to PKR. (**B**) Immunoprecipitation detecting PKR dimerization. HEK293T cells were co-transfected p3xFlag-CMV-PKR with pCMV-N-Myc-PKR or pcDNA3.1a-vector or different doses of pcDNA3.1a-TRIM21 (1 μg or 2 μg) for 48 h. The anti-Flag tag antibody was used to perform the immunoprecipitation and detect Flag-PKR, the anti-Myc tag antibody was used to detect Myc-PKR and the anti-V5 tag antibody was used to detect V5-TRIM21. GAPDH was used as an internal control. The PKR dimerization effected by TRIM21 was quantified by densiometric analysis which were normalized to the value of PKR dimerization in TRIM21 non-transfection. The data shown are mean ± SD. *P* values were determined by Student’s *t*-test. NS, no significance difference. (**C**) IP analysis of the interaction of PP1α and TRIM21 as well as PP1α and PKR in HEK293T cells by co-transfection with p3xFlag-CMV-PKR and pCMV-N-Myc-PP1α or p3xFlag-CMV-TRIM21 and pCMV-N-Myc-PP1α for 48 h. β-actin was used as an internal control. (**D-E**) Endogenous PP1α and TRIM21 interaction was analyzed by immunoprecipitation in A549 cells infected with VSV (MOI = 0.2) for 6 h (D) or stimulated with TG (10 μM or 20 μM) for 12 h (E). The amounts of PP1α bound TRIM21 were quantified by densiometric analysis which were normalized to the value in the control group. The data shown are mean ± SD. *P* values were determined by Student’s *t*-test. *p<0.05, **P<0.01. The TRIM21 antibody was used to perform immunoprecipitation and the indicated proteins were detected by the corresponding antibodies. β-actin was used as an internal control. (**F**) In vitro immunoprecipitation analysis of the interaction between GST-TRIM21 protein and Flag-PP1α protein purified from bacteria. Experiments were independently repeated two or three times with similar results.

PKR is a serine–threonine kinase comprised of a kinase domain and two dsRNA binding domains that regulate its activity. Upon engagement with dsRNA in the cytoplasm, PKR undergoes homodimerization and subsequent rapid autophosphorylation at Thr446 and Thr451, increasing its catalytic activity. Therefore, there are three steps in PKR activation: dsRNA detection, homodimerization and autophosphorylation. To investigate how TRIM21 represses PKR activation, we examined whether TRIM21 affects the processes of PKR activation. First, we performed RNA immunoprecipitation (RIP) analysis to explore whether TRIM21 affects PKR binding to viral RNA in HEK293T cells after infection with VSV. Consistently, PKR constitutively bound to viral RNA, and *TRIM21* knockdown did not abolish their interaction (Fig 3A), suggesting that TRIM21 has no effect on the detection of dsRNA by PKR. To test whether TRIM21 affects PKR homodimerization, we delivered pFlag-tagged PKR and pMyc-tagged PKR together with different doses of pV5-tagged TRIM21 into HEK293T cells. Notably, overexpression of TRIM21 did not abolish the interaction between Flag-tagged PKR and Myc-tagged PKR (Fig 3B), suggesting that TRIM21 has no effect on PKR homodimerization, which implies that TRIM21 may impair PKR phosphorylation. Given that PKR phosphorylates itself and TRIM21 has no effect on PKR dimerization, which is indispensable for the kinase activity of PKR, we speculated that TRIM21 may target the phosphatases of PKR to inactive PKR. To confirm our hypothesis, we focused on a phosphatase, serine/threonine protein phosphatase type 1 alpha (PP1α), the key phosphatase of PKR [19]. Indeed, PP1α interacted with PKR as well as TRIM21 (Fig 3C), the endogenous interaction between PP1α and TRIM21 was also evidenced, and their interaction was enhanced upon VSV infection or TG stimulation (Fig 3D and 3E). Moreover, an in vitro pull-down assay with purified recombinant proteins demonstrated a direct interaction between TRIM21 and PP1α (Fig 3F). All the data indicated that TRIM21 may target PP1α for PKR inactivation.

### TRIM21 promotes PP1α polyubiquitination

**Fig 4:**
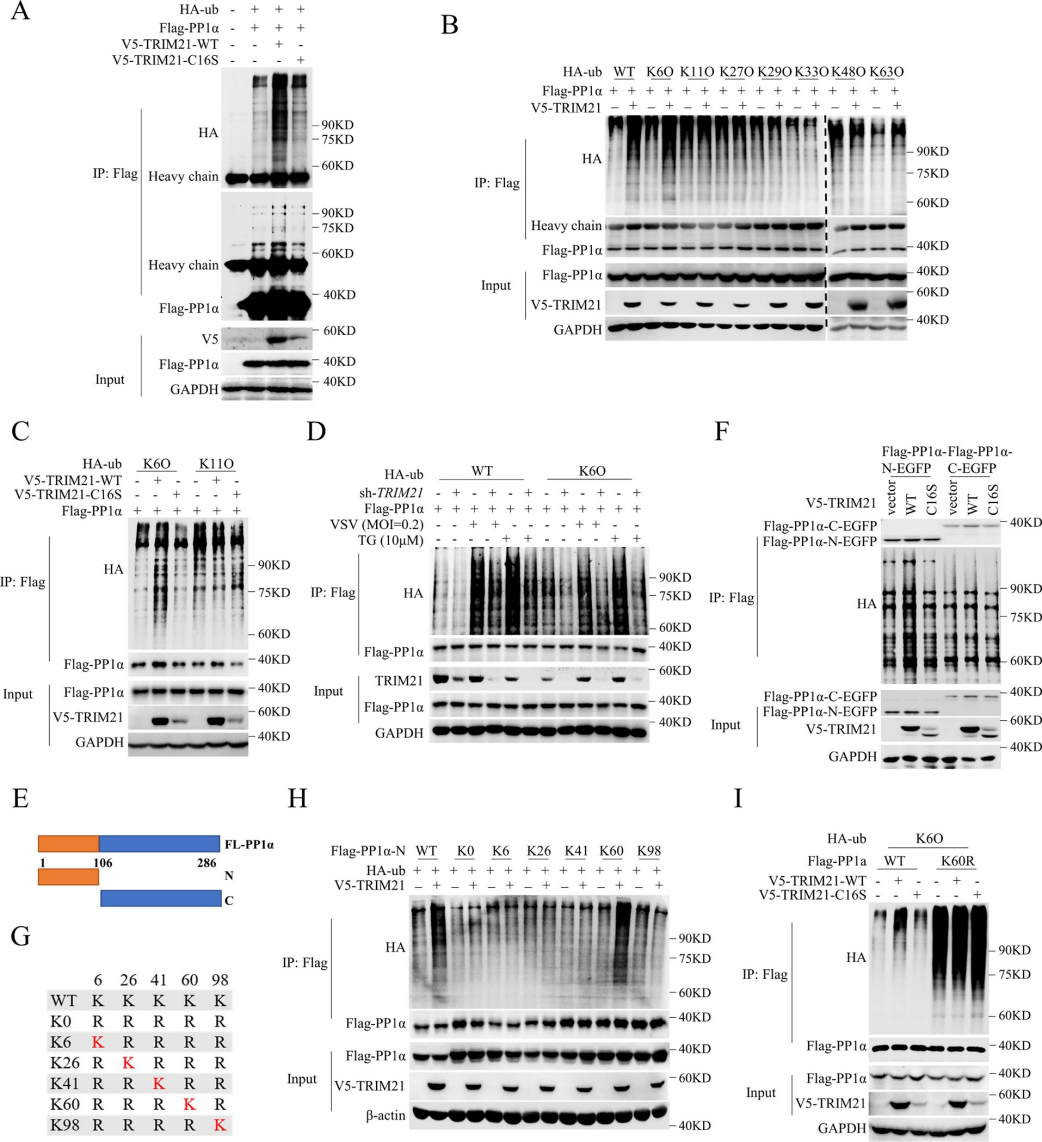
TRIM21 promotes the polyubiquitination of PP1α. (**A)** IP analysis of the ubiquitination of PP1α in HEK293T cells by co-transfecting p3xFlag-CMV-PP1α with pcDNA3.1a-vector or pcDNA3.1a-TRIM21-WT or pcDNA3.1a-TRIM21-C16S as well as HA-ub for 48 h. The anti-Flag antibody was used to perform immunoprecipitation and detect Flag-PP1α, the anti-V5 antibody was used to detect V5-TRIM21-WT/C16S and the anti-HA antibody was used to detect HA-ub. GAPDH was used as an internal control. (**B**) Ubiquitination analysis ubiquitination of PP1α. HEK293T cells were co-transfected with p3xFlag-CMV-PP1α and pcDNA3.1a-TRIM21 as well as the indicated HA-tagged ubiquitin mutants for 48 h. The anti-Flag antibody was used to perform immunoprecipitation and detect Flag-PP1α, the anti-V5 antibody was used to detect V5-TRIM21 and the anti-HA antibody was used to detect HA-ub. GAPDH was as an internal control. (**C**) Ubiquitination analysis the K6-linked ubiquitination of PP1α. HEK293T cells were co-transfected p3xFlag-CMV-PP1α with pcDNA3.1a-vector or pcDNA3.1a-TRIM21-WT or pcDNA3.1a-TRIM21-C16S as well as the indicated HA-tagged ubiquitin mutants for 48 h. Immunoprecipitation was performed with anti-Flag antibody. Ubiquitination was detected by anti-HA antibody and GAPDH was used as an internal control. (**D**) Ubiquitination analysis the K6-linked ubiquitination of PP1α under stress. HEK293T cells pre-infected with lentivirus-sh-vector or lentivirus-sh-*TRIM21* for 12 h were co-transfected p3xFlag-CMV-PP1α with HA-ub-WT or HA-ub-K6O for 36 h, then infected with VSV (MOI = 0.2) for 6 h or treated with TG (10 μM) for 12 h. Immunoprecipitation was performed with anti-Flag antibody. Ubiquitination was detected by anti-HA antibody, TRIM21 was detected by anti-TRIM21 antibody and GAPDH was used an internal control. (**E**) A schematic diagram of PP1α truncations. (**F**) Ubiquitination of C-terminus and N-terminus of PP1α in HEK293T cells by co-transfecting p3xFlag-CMV-PP1α-C-EGFP or p3xFlag-CMV-PP1α-N-EGFP with pcDNA3.1a-vector or pcDNA3.1a-TRIM21-WT or pcDNA3.1a-TRIM21-C16S as well as HA-ub for 48 h. Immunoprecipitation was performed with anti-Flag antibody. Ubiquitination was detected by anti-HA antibody, V5 was detected by anti-V5 antibody and GAPDH was used an internal control. (**G-H**) TRIM21 promotes the polyubiquitination of PP1α on Lys60. Mutants of only one lysine residue retained within N-terminal PP1α (G). HEK293T cells were co-transfected pcDNA3.1a-vector or pcDNA3.1a-TRIM21 with or the mutants of p3xFlag-CMV-PP1α-N for 48 h. Ubiquitination and immunoblotting were performed with the antibodies as described in (F) (H). (**I**) TRIM21 promotes K6-linked ubiquitination of PP1α on Lys60. HEK293T cells were co-transfected p3xFlag-CMV-PP1α-WT or p3xFlag-CMV-PP1α-K60R with pcDNA3.1a-vector or pcDNA3.1a-TRIM21-WT or pcDNA3.1a-TRIM21-C16S as well as HA-ub-K6O for 48 h. Ubiquitination and immunoblotting assays were performed with the antibodies as described in (F). Experiments were independently repeated two or three times with similar results.

Since TRIM21 targets PP1α and TRIM21 inhibits PKR activation in a manner dependent on its ubiquitin ligase activity, we investigated whether TRIM21 can ubiquitinate PP1α. We delivered pTRIM21-WT or pTRIM21-C16S together with pFlag-tagged PP1α and pHA-ub into HEK293T cells and found increased ubiquitination of PP1α in TRIM21-WT-transfected cells but not in TRIM21-C16S-transfected cells (Fig 4A), suggesting that TRIM21 promotes the ubiquitination of PP1α. Different types of polyubiquitin linkages have distinct functions. We cotransfected pFlag-tagged PP1α into HEK293T cells with individual ubiquitin mutants (K6O, K11O, K27O, K29O, K33O, K48O or K63O), each of which contained only one lysine residue available for modification. Apparently, TRIM21 specifically promoted K6-linked ubiquitination of PP1α; however, the K6-linked ubiquitination of PP1α disappeared by inactivation of the ubiquitination ligase activity of TRIM21 (Fig 4B and 4C). Moreover, as a negative control, neither TRIM21-WT nor TRIM21-C16S promoted K11-linked ubiquitination of PP1α (Fig 4C). Furthermore, TRIM21-mediated K6-linked ubiquitination of PP1α was enhanced under conditions of viral infection or TG treatment (Fig 4D). These data demonstrated that TRIM21 catalyzes K6-linked ubiquitination of PP1α. Next, we examined which lysine residue of PP1α is ubiquitinated by TRIM21. PP1α was divided into two fragments, the N-terminus and C-terminus, and we delivered the N-terminus or C-terminus of PP1α together with TRIM21-WT or TRIM21-C16S as well as HA-ub into HEK293T cells (Fig 4E). Co-IP assays revealed that the N-terminus of PP1α is ubiquitinated by TRIM21 (Fig 4F), demonstrating that TRIM21 ubiquitinates PP1α at the N-terminus. There are 5 lysine residues in the N-terminus of PP1α, and we generated the mutant PP1α-K0, in which all of the lysine residues in the N-terminus of PP1α were replaced with arginine. Then, we reintroduced individual lysine residues into PP1α-K0 to generate single lysine mutants (Fig 4G). Co-IP assay results showed that the K60 mutant of PP1α is ubiquitinated by TRIM21 (Fig 4H), suggesting that TRIM21 may catalyze the K6-linked ubiquitination of PP1α on Lys60. To confirm our conclusion, we constructed a PP1α mutant with replacement of the lysine residue with an arginine residue at K60 (PP1α-K60R). Consistently, TRIM21 no longer ubiquitinated PP1α by K60 mutation (Fig 4I). Collectively, these data demonstrated that TRIM21 ubiquitinates PP1α at Lys60.

### TRIM21 inhibits the activation of the PKR signaling pathway by catalyzing the K6-linked ubiquitination of PP1α

**Fig 5:**
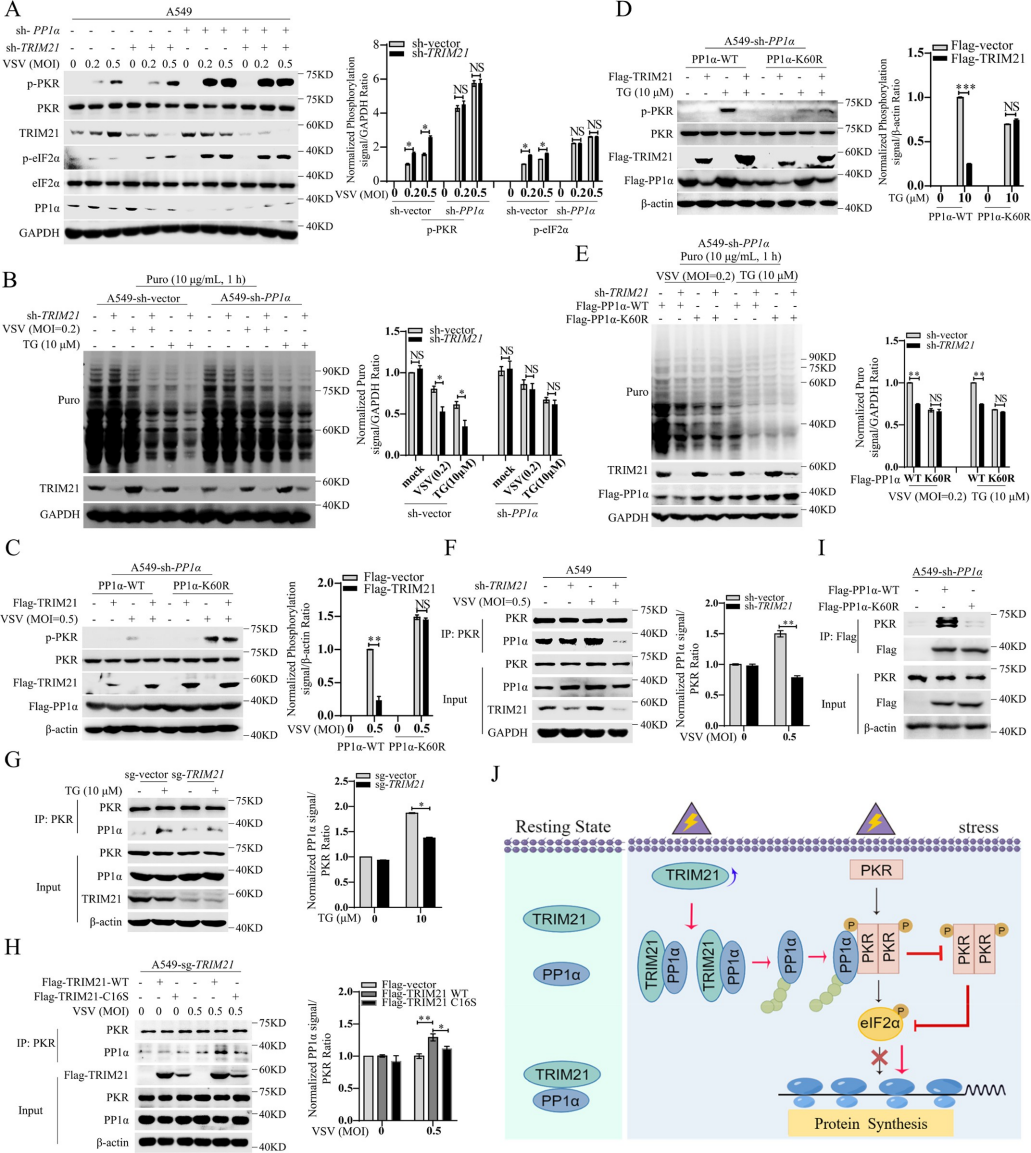
TRIM21 inhibits PKR activation by augmenting PKR-PP1α interaction. (**A**) A549 cells were infected with lentivirus-sh-vector (sh-vector) or lentivirus-sh- *PP1α* (sh-*PP1α*) for 12 h, and infected with lentivirus-sh-vector (sh-vector) or lentivirus-sh-*TRIM21* (sh-*TRIM21*) for 48 h, then infected by VSV (MOI = 0.2 or 0.5) for 6 h. The indicated proteins were detected by western blot by using the corresponding antibodies. GAPDH was used as an internal control. (**B**) Puromycin incorporation assays demonstrated the cellular protein synthesis in wild-type A549 cells (sh-vector) or *PP1α*-silenced A549 cells (sh-*PP1α*). Cells were infected with lentivirus-sh-vector (sh-vector) or lentivirus-sh-*TRIM21* (sh-*TRIM21*) for 48 h, then infected by VSV (MOI = 0.2) for 6 h or stimulated with TG (10 μM) for 12 h. The indicated proteins were detected by western blot by using the corresponding antibodies. GAPDH was used as a control. (**C-D**) *PP1α*-silenced A549 cells (sh-*PP1α*) were infected with lentivirus-Flag-PP1α-WT or lentivirus-Flag-PP1α-K60R for 48 h, then infected with VSV (MOI = 0.5) for 6 h (C) or stimulated with TG (10 μM) for 12 h (D). The indicated proteins were detected by western blot by using the corresponding antibodies. β-actin was used as an internal control. (**E**) Puromycin incorporation assays demonstrated the cellular protein synthesis in *PP1α*-silenced A549 cells (sh-*PP1α*). Cells were infected with lentivirus-sh-vector (sh-vector) or lentivirus-sh-*TRIM21* (sh-*TRIM21*) for 12 h, and infected with lentivirus-Flag-PP1α-WT or Lentivirus-Flag-PP1α-K60R for 48 h, then infected by VSV (MOI = 0.2) for 6 h or stimulated with TG (10 μM) for 12 h. GAPDH was used as a control. (**F-G**) TRIM21 blocks PKR-PP1α interaction. A549 cells were infected with lentivirus-sh-vector (sh-vector) or lentivirus-sh-*TRIM21* (sh-*TRIM21*) for 48 h, then infected by VSV (MOI = 0.5) for 6 h. The interaction of PP1α and PKR was analyzed by IP and western blot (F). The wild-type A549 cells (sg-vector) or *TRIM21*-deficient A549 cells (sg-*TRIM21*) were stimulated with TG (10 μM) for 12 h, The interaction of PP1α and PKR was analyzed by IP and western blot (G). GAPDH (F) or β-actin (G) was used as an internal control. (**H**) *TRIM21*-deficient A549 cells (sg-*TRIM21*) were infected with lentivirus-Flag-TRIM21-WT or lentivirus-Flag-TRIM21-C16S for 48 h, then infected by VSV (MOI = 0.5) for 6 h. The interaction of PP1α and PKR was analyzed by IP and western blot. β-actin was used as an internal control. (**I**) *PP1α*-silenced A549 cells (sh-*PP1α*) were infected with lentivirus-Flag-PP1α-WT or lentivirus-Flag-PP1α-K60R for 48 h. The interaction of PP1α and PKR was analyzed by IP and western blot. β-actin was used as an internal control. (**J**) Schematic model of TRIM21 regulating the translation initiation. Upon stress stimulation, TRIM21 augments the polyubiquitination of PP1α, enhancing the PKR-PP1α interaction, leading to PKR inactivation and subsequent release of protein synthesis inhibition. The relative ratios of p-PKR or p-eIF2α in **(A, C and D)**, the relative ratios of puro signal in **(B, E)** and the amounts of PP1α bound PKR in **(F-H)** were quantified by densiometric analysis, which were normalized to the value in the control group. Experiments were independently repeated two or three times with similar results, and the data shown are mean ± SD. *P* values were determined by Student’s *t*-test. *p<0.05, **P<0.01, ***p<0.001, NS, no significance difference.

To determine whether TRIM21 represses PKR activation via TRIM21-mediated K6-linked ubiquitination of PP1α, we knocked down the expression of *TRIM21* in *PP1α*-silenced A549 cells. Consistent with our data, *TRIM21* knockdown substantially enhanced PKR activation, while this phenomenon was lost by additional knockdown of *PP1α* (Fig 5A). Consequently, virus- or TG-induced global protein synthesis inhibition reversed by TRIM21 was also blocked by *PP1α* knockdown (Figs 5B and S2A). To confirm this conclusion, we constructed a mutant with phosphatase activity inactivation of PP1α (PP1α-H248K) and delivered it into *PP1α*-silenced A549 cells. Consistently, TRIM21 lost the ability to inactivate PKR by PP1α-H248K introduction upon VSV infection or TG stimulation (S2B and S2C Fig). These data demonstrated that TRIM21 inhibits PKR activation via PP1α. Next, we explored whether K6-linked ubiquitination of PP1α is essential for TRIM21-mediated PKR inactivation. We reintroduced wild-type PP1α (PP1α-WT) or K60-mutant PP1α (PP1α-K60R) together with pFlag-tagged TRIM21 into *PP1α*-silenced cells to detect the phosphorylation of PKR under viral infection or TG treatment. The phosphorylation of PKR was inhibited, and the inhibition of RNA translation was reversed by TRIM21 in PP1α-WT-transfected cells; however, this phenomenon disappeared in PP1α-K60R-transfected cells after VSV infection or TG treatment (Fig 5C–5E), demonstrating that TRIM21-mediated ubiquitination of PP1α is crucial for PKR inactivation. Collectively, these data suggested that TRIM21 impairs PKR activation by promoting the K6-linked ubiquitination of PP1α.

### TRIM21-mediated K6-linked ubiquitination enhances the PKR-PP1α interaction

To determine how TRIM21-mediated polyubiquitination of PP1α represses PKR activation, we first investigated whether TRIM21 affects the stability of the PP1α protein. TRIM21 did not alter the protein level of PP1α with or without VSV infection or TG treatment (S2D–S2F Fig), suggesting that TRIM21 does not affect the stability of the PP1α protein. Previous studies have demonstrated that K6-linked ubiquitination can affect protein–protein interactions [20,21], and therefore we speculated that TRIM21-mediated K6-linked ubiquitination may play a role in the PKR-PP1α interaction. To confirm our hypothesis, we performed a co-IP assay to examine the effect of the PKR-PP1α interaction by TRIM21. Although TRIM21 had no effect on the interaction of PKR and PP1α in the resting state, *TRIM21* knockdown significantly reduced their interaction upon VSV infection or TG stimulation (Fig 5F and 5G). These data indicated that TRIM21 can enhance the interaction between PKR and PP1α. Next, we examined whether the enhancement of the PKR-PP1α interaction is dependent on TRIM21-mediated K6-linked ubiquitination of PP1α. Immunoprecipitation showed that the interaction between PKR and PP1α is increased in TRIM21-WT-reconstituted sg-*TRIM21* cells but not in TRIM21-C16S-reconstituted sg-*TRIM21* cells upon VSV infection (Fig 5H). Moreover, the interaction between PP1α and PKR was abolished by PP1α-K60R (Fig 5I), suggesting that TRIM21-mediated ubiquitination of PP1α is essential for the enhancement of the PKR-PP1α interaction. Collectively, extracellular stimuli, such as viral infection or TG treatment, promote the interaction between TRIM21 and PP1α, which results in K6-linked ubiquitination of PP1α. Ubiquitinated PP1α enhances its interaction with PKR, resulting in PKR dephosphorylation and PKR inactivation, which subsequently inhibits eIF2α activation, leading to the release of PKR activation-mediated protein synthesis shutdown (Fig 5J).

### TRIM21 restricts viral infection by inhibiting PKR activation

**Fig 6:**
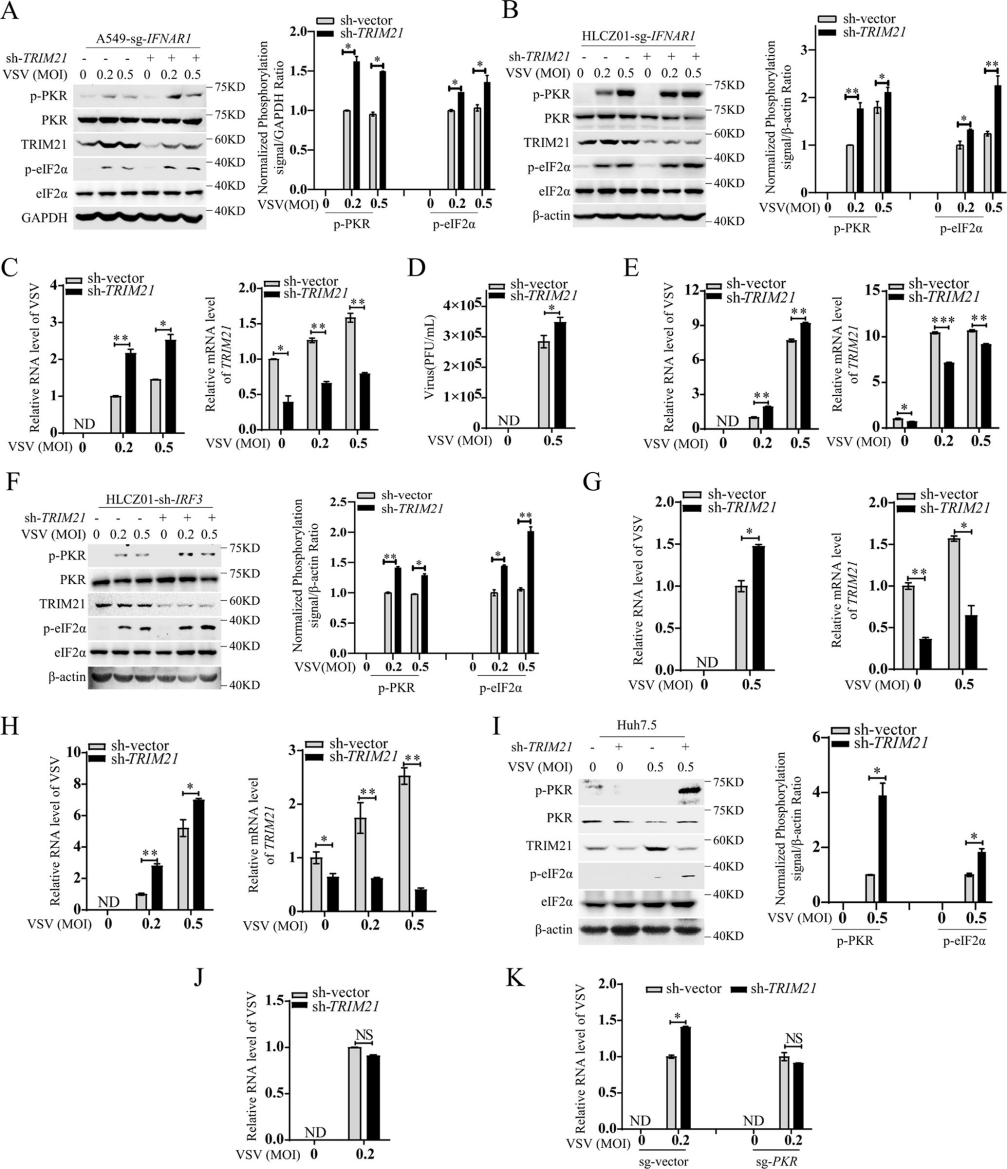
TRIM21 restricts viral infection by inhibiting the activation of PKR signaling pathway. (**A-B**) *IFNAR1*-deficient A549 cells (A) or *IFNAR1*-deficient HLCZ01 (B) cells were infected with lentivirus-sh-vector (sh-vector) or lentivirus-sh-*TRIM21* (sh-*TRIM21*) for 48 h, then infected with VSV (MOI = 0.2 or 0.5) for 6 h. The indicated proteins were detected by western blot. GAPDH (A) or β-actin (B) was used as an internal control. (**C**) RT-qPCR analysis of the VSV RNA or the mRNA levels of *TRIM21* in *IFNAR1*-deficient A549 cells (sg-*IFNAR1*) infected with lentivirus-sh-vector (sh-vector) or lentivirus-sh-*TRIM21* (sh-*TRIM21*) for 48 h, then infected with VSV (MOI = 0.2 or 0.5) for 6 h. (**D**) Plaque assay analysis of the VSV titers in *IFNAR1*-deficient HLCZ01 cells infected with lentivirus-sh-vector (sh-vector) or lentivirus-sh-*TRIM21* (sh-*TRIM21*) for 48 h, then infected with VSV (MOI = 0.5) for 6 h. (**E**) *IFNAR1/STAT2* double-deficient A549 cells were infected with lentivirus-sh-vector (sh-vector) or lentivirus-sh-*TRIM21* (sh-*TRIM21*) for 48 h, then infected with VSV (MOI = 0.2 or 0.5) for 6 h. RT-qPCR analysis of the VSV RNA or the mRNA level of *TRIM21*. (**F**) HLCZ01-sh-*IRF3* cells were infected with lentivirus-sh-vector (sh-vector) or lentivirus-sh-*TRIM21* (sh-*TRIM21*) for 48 h, then infected with VSV (MOI = 0.2 or 0.5) for 6 h. The indicated proteins were detected by western blot. (**G**) RT-qPCR analysis of the VSV RNA or the mRNA level of *TRIM21* in *IRF3*-silenced HLCZ01 cells (HLCZ01-sh-*IRF3*) infected with lentivirus-sh-vector (sh-vector) or lentivirus-sh-*TRIM21* (sh-*TRIM21*) for 48 h, then infected with VSV (MOI = 0.5) for 6 h. (**H**) Huh7.5 cells infected with lentivirus-sh-vector (sh-vector) or lentivirus-sh-*TRIM21* (sh-*TRIM21*) for 48 h, then infected with VSV (MOI = 0.2 or 0.5) for 6 h. RT-qPCR analysis of the level of VSV RNA or *TRIM21* mRNA. (**I**) Huh7.5 cells infected with lentivirus-sh-vector (sh-vector) or lentivirus-sh-*TRIM21* (sh-*TRIM21*) for 48 h, then infected with VSV (MOI = 0.5) for 6 h. The indicated proteins were detected by western blot. (**J**) *IFNAR1/PKR* double-deficient A549 cells were infected with lentivirus-sh-vector (sh-vector) or lentivirus-sh-*TRIM21* (sh-*TRIM21*) for 48 h, then infected with VSV (MOI = 0.2) for 6 h. The levels of VSV RNA were examined by RT-qPCR. (**K**) Wild-type (sg-vector) or *PKR*-deficient (sg-*PKR*) Huh7.5 cells were infected with lentivirus-sh-vector (sh-vector) or lentivirus-sh-*TRIM21* (sh-*TRIM21*) for 48 h, then infected with VSV (MOI = 0.2) for 6 h. The levels of VSV RNA were examined by RT-qPCR. Experiments were independently repeated two or three time with similar results. The relative ratios of p-PKR or p-eIF2α in **(A, B, F and I)** were quantified by densiometric analysis, which were normalized to the value in the control group with two or three independent repeats. The mRNA data in **(C-E, G-H, J-K)** from two or three independent experiments. The data shown are mean ± SD. *P* values were determined by Student’s *t*-test. *p<0.05, **p<0.01, ***p<0.001. NS, no significance difference. ND, not detected.

Several studies have demonstrated that PKR activation is essential for viral escape by inhibiting the RNA translation of antiviral effectors [10,22]. Given the negative regulatory role of TRIM21 in PKR activation, we speculated that TRIM21 has the ability to restrict viral infection by inhibiting PKR-mediated translational shutdown. To confirm our hypothesis, we constructed *IFNAR1-*deficient cell lines in both A549 cells (A549-sg-*IFNAR1*) and HLCZ01 cells (HLCZ01-sg-*IFNAR1*), as TRIM21 is able to inhibit viral infection by promoting IFN production (S3A Fig). Notably, the levels of phosphorylated PKR and eIF2α were repressed by TRIM21 in the cells upon VSV infection (Fig 6A and 6B), and silencing *TRIM21* increased viral replication and production in these cells (Fig 6C and 6D). The antiviral role of TRIM21 was also evidenced in A549-sg-*IFNAR1* cells upon SeV infection (S3B Fig). To exclude the possible role of the type III IFN-mediated antiviral response by viral infection, we knocked down *STAT2* to abolish the type III IFN signaling pathway in A549-sg-*IFNAR1* cells (S3C Fig). Notably, *TRIM21* deficiency still increased the replication of VSV in A549-sg-*IFNAR1* cells with *STAT2* knockdown (Fig 6E). Furthermore, we investigated *IRF3*-silenced cells, in which IFN production was abolished upon viral infection. Silencing *TRIM21* augmented viral replication and PKR activation upon VSV infection in these cells (Figs 6F and 6G and S3D). Similarly, the replication of VSV and SeV (Figs 6H and S3E), as well as the phosphorylation of PKR and eIF2α, was also increased by *TRIM21* deficiency (Fig 6I) in Huh7.5 cells, in which one of the main pattern recognition receptors of RNA viruses, RIG-I, is deficient. These data demonstrated that TRIM21 restricts viral infection in an IFN-independent manner.

Next, we examined whether this IFN-independent antiviral role of TRIM21 restricts viral infection by inhibiting PKR activation. *PKR/IFNAR1* double-deficient A549 cell lines were constructed (S3F Fig), and the antiviral function of TRIM21 was lost by *PKR* knockout (Fig 6J). Likewise, *PKR*-deficient Huh7.5 cells with *TRIM21* knockdown showed similar replication of VSV and SeV compared to that of the control cells (Figs 6K, S3G and S3H). Moreover, TRIM21 also lost the ability to restrict viral infection by overexpression of TRIM21 in *PKR/TRIM21* double-deficient Huh7.5 cells (S3I and S3J Fig), suggesting that TRIM21 restricts viral infection via PKR.

### TRIM21 restricts viral infection by releasing PKR-mediated inhibition of the RNA translation of intrinsic antiviral genes

A previous study reported that PKR-mediated inhibition of the RNA translation of IFN-stimulated genes (ISGs) contributes to restoring HCV replication by IFN treatment [22]. Thus, we examined whether TRIM21 can inhibit HCV replication by inhibiting PKR activation. Therefore, we performed an investigation in HCV-infected Huh7.5 cells treated with IFN. Similarly, in HCV-infected Huh7.5 cells, *TRIM21* knockdown augmented IFN-mediated PKR activation and inhibited the RNA translation of ISGs, such as ISG15 and STAT2 (S4A Fig). Consistently, the levels of HCV RNA, NS3 protein and viral particles in the supernatant were upregulated by *TRIM21* knockdown (S4B–S4D Fig). The replication of H77-S, HCV genotype 1a, was also inhibited by TRIM21 in response to IFN-α (S4E Fig). These data indicated that TRIM21 restricts HCV infection by reversing the *PKR*-mediated inhibition of ISG protein synthesis.

**Fig 7:**
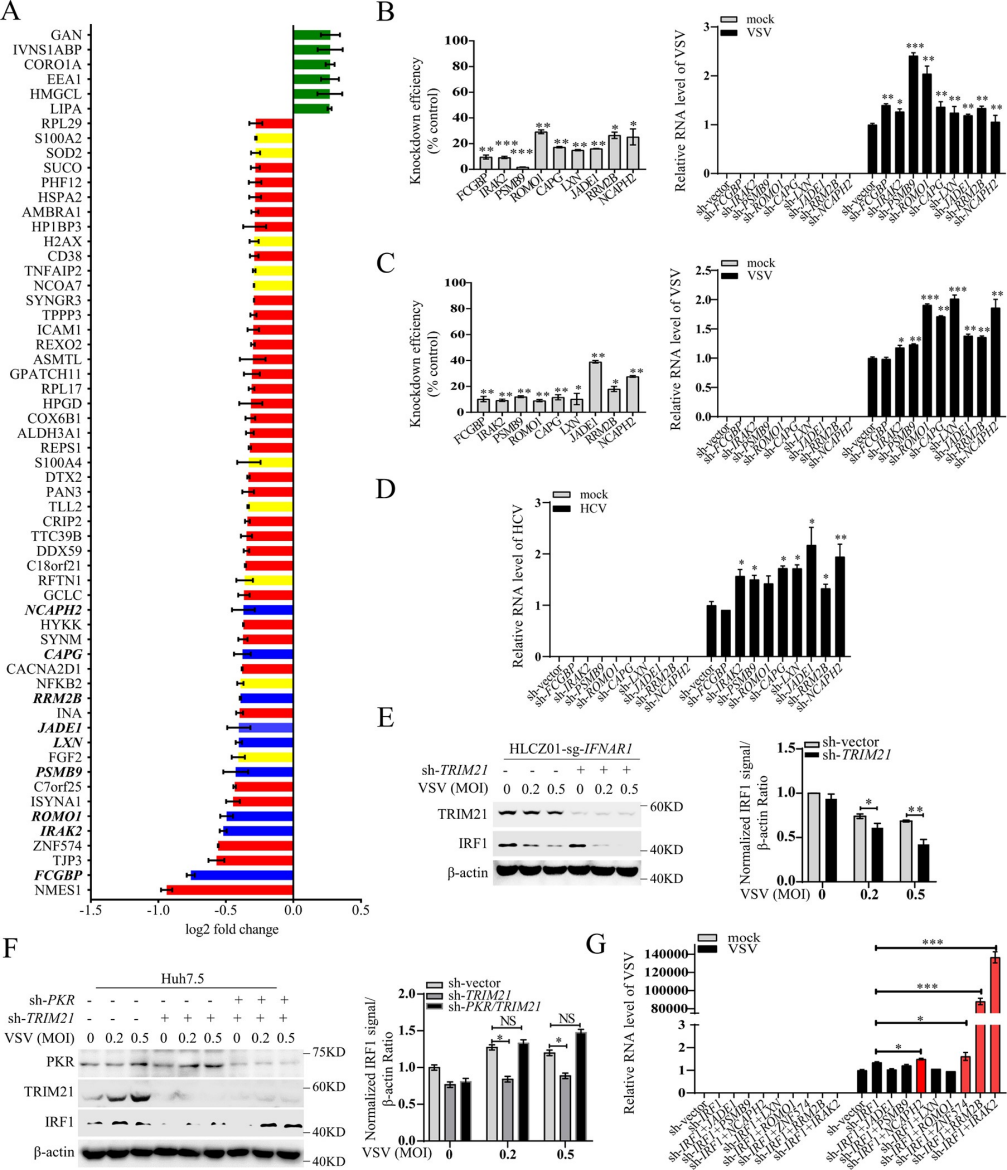
TRIM21 resists viral infection through inhibiting PKR activation-mediated global translation shutdown. (**A**) Proteome analysis of the reduced genes in *IFNAR1*-deficient A549 cells (sg-*IFNAR1*) and *IFNAR1/TRIM21* double-deficient cells (sg-*IFNAR1/TRIM21*). The cells were infected with VSV(MOI = 0.2) for 6 h. Known antiviral effectors were marked with yellow and the effectors with antiviral function verified in our study were marked with blue. (**B-C**) RT-qPCR analysis of VSV RNA or the mRNA levels of the indicated genes in A549 cells (B) or Huh7 cells (C) infected with lentivirus expressing shRNAs targeting the indicated genes for 48 h, then infected with VSV (MOI = 0.2) for 6 h. (**D**) Huh7.5 cells were infected with Lentivirus expressing shRNAs targeting the indicated genes for 12 h, then infected with HCV (MOI = 0.1) for 72 h. HCV RNA level was analyzed by RT-qPCR. (**E)** *IFNAR1*-deficient HLCZ01 cells (HLCZ01-sg-*IFNAR1*) were infected with lentivirus-sh-vector (sh-vector) or lentivirus-sh-*TRIM21* (sh-*TRIM21*) for 48 h, then infected with VSV (MOI = 0.2 or 0.5) for 6 h. IRF1 protein was analyzed by western blot. β-actin was detected as control. The relative amounts of IRF1 proteins were quantified by densiometric analysis, which were normalized to the value in the control group. The data shown are mean ± SD. *P* values were determined by Student’s *t*-test. *p<0.05; **p<0.01. (**F**) Huh7.5 cells were infected with lentivirus-sh-vector (sh-vector) or lentivirus-sh-*PKR* (sh-*PKR*), or lentivirus-sh-vector (sh-vector) or lentivirus-sh-*TRIM21* (sh-*TRIM21*) for 48 h, then infected with VSV (MOI = 0.2 or 0.5) for 6 h. IRF1 protein was analyzed by western blot. β-actin was detected as control. The relative amounts of IRF1 proteins were quantified by densiometric analysis, which were normalized to the value in the control group. The data shown are mean ± SD. *P* values were determined by Student’s *t*-test. *p<0.05, NS, no significant difference. (**G**) Huh7 cells were infected with Lentivirus expressing shRNAs targeting the indicated genes for 48 h, then infected with VSV (MOI = 0.2) for 6 h. The levels of VSV RNA were examined by RT-qPCR. Experiments were independently repeated two or three times with similar results. The data shown are mean ± SD. *P* values were determined by Student’s *t*-test. *p<0.05; **p<0.01, ***p<0.001, NS, no significant difference.

Given its beneficial role in protein synthesis of ISGs, we speculated that the IFN-independent antiviral role of TRIM21 in *IFNAR1*-deficient cells is due to promote protein synthesis of the intrinsic antiviral genes in the host. To confirm our hypothesis, we first investigated whether TRIM21 affects global protein synthesis in *IFNAR1*-deficient cells by assessing puro-labeled nascent peptides. Consistent with the results above that TRIM21 inhibits PKR signaling pathway activation, VSV-induced nascent protein synthesis inhibition was reversed by overexpression of TRIM21 in *IFNAR1/TRIM21* double-deficient cells (S4F–S4H Fig), indicating that TRIM21 can release virus-induced protein synthesis inhibition in *IFNAR1*-deficient cells, which implies that TRIM21 may have a role in reversing the inhibition of RNA translation of antiviral effectors. Thus, to identify the specific TRIM21-PKR-regulated antiviral effectors, we performed proteomic analysis in *IFNAR1*-deficient cells and *IFNAR1/TRIM21* double-deficient cells upon VSV infection (Fig 7A). The proteomics assay showed that the protein abundances of 53 genes with significant differences were downregulated in *IFNAR1*-deficient cells with *TRIM21* depletion relative to those of *IFNAR1*-deficient cells (Fig 7A). Notably, these genes included some known antiviral effectors, such as proteasome 20S subunit beta 9 (*PSMB9*), nuclear factor kappa B subunit 2 (*NFKB2*), OUT deubiquitinase 4 (*OTUD4*), methyl-CpG binding protein 2 (*MECP2*), H2A.X variant histone (*H2AX*), nuclear receptor coactivator 7 (*NCOA7*), superoxide dismutase 2 (*SOD-2*), S100 calcium binding protein A2 (*S100A2*), raftlin, lipid raft linker 1 (*RFTN1*), S100 calcium binding protein A2 (*S100A4*), fibroblast growth factor 2 (*FGF2*), tolloid like 2 (*TLL2*), and TNF-induced protein 2 (*TNFAIP2*) (marked in yellow) [23–35], in addition to multiple genes with no previously recognized antiviral function (marked in red and green). Among the genes with unknown antiviral function, we focused on nine genes (marked in blue) that were significantly downregulated in *TRIM21* knockout cells. A549 cells were infected with lentivirus expressing shRNAs targeting IgG Fc-binding protein (*FCGBP*), interleukin-1 receptor-associated kinase-like 2 (*IRAK2*), proteasome subunit beta type-9 (*PSMB9*), reactive oxygen species modulator 1 (*ROMO1*), latexin (*LXN*), Jade family PHD finger 1 (*JADE1*), ribonucleotide reductase regulatory TP53 inducible subunit M2B (*RRM2B*), non-SMC condensin II complex subunit H2 (*NCAPH2*), or capping actin protein (*CAPG*) and then infected with VSV. Notably, knockdown of these genes enhanced the replication of VSV (Fig 7B). Similar results were obtained in VSV-infected hepatocytes and Huh7 cells (Fig 7C). Of note, these genes could constitutively restrict HCV replication in HCV-infected Huh7.5 cells (Fig 7D). However, FCGBP had no effect on the replication of VSV or HCV in hepatocytes, which demonstrated that the function of FCGBP in hepatocytes is distinctive. These data supported the hypothesis that reversing the PKR-mediated inhibition of antiviral gene protein synthesis by TRIM21 is sufficient for viral clearance.

### TRIM21 restricts viral infection through IRF1-dependent and IRF1-independent mechanisms

It has been reported that basal expression of interferon regulatory factor 1 (IRF1) drives hepatocyte resistance to multiple RNA viruses by maintaining constitutive transcription of antiviral effectors, while the level of IRF1 protein is tightly regulated by PKR [10,26,36]. Given that TRIM21 suppresses PKR activation, we investigated whether TRIM21 can resist viral infection by promoting the protein synthesis of IRF1. Notably, silencing *TRIM21* reduced IRF1 protein levels in *IFNAR1*-deficient HLCZ01 cells infected with VSV (Fig 7E), suggesting that TRIM21 can increase the abundance of IRF1 protein. Similarly, the levels of IRF1 protein were reduced in *TRIM21*-silenced Huh7.5 cells with VSV infection; however, the protein levels of IRF1 were restored by additional knockdown of PKR (Fig 7F), indicating that TRIM21 promotes the protein synthesis of IRF1 by negatively regulating PKR activation. Next, we examined whether the antiviral role of TRIM21 is dependent on IRF1 in Huh7 cells. Compared with the results of single knockdown of the upregulated genes (Fig 7C), several genes, such as *JADE1*, *PSMB9*, *LXN* and *ROMO1*, lost their antiviral ability after IRF1 ablation (Fig 7G), indicating an IRF1-dependent antiviral role of TRIM21. However, IRF1 had no effect on the antiviral function of *NCAPH2*, *ZNF574*, *RRM2B* and *IRAK2* (marked in red), indicating an IRF1-independent antiviral role of TRIM21 (Fig 7G). These data indicated that some genes are regulated by both TRIM21 and IRF1, contributing to the IRF1-dependent antiviral response; however, some genes, such as *NCAPH2*, *ZNF574*, *RRM2B* and *IRAK2*, are regulated by TRIM21 rather than IRF1, causing an IRF1-independent antiviral response and even enhancing the antiviral efficiency of IRF1. Collectively, these data revealed that TRIM21 restricts viral infection in IRF1-dependent and IRF1-independent manners.

## Discussion

In response to stimulation, cellular protein homeostasis is tightly regulated by various strategies in all steps of RNA translation, including initiation, elongation, and termination, to make cells rapidly adapt to the changed extracellular or intracellular environment [2]. When translation is completed, nascent proteins are always modified in various manners, such as by ubiquitination by TRIM proteins, to confer the specific function of proteins or regulate protein fate. The crucial role of TRIM proteins in posttranslational modification has been widely investigated [14]; however, the role of TRIM proteins in regulating protein synthesis initiation is not clear. In this study, we found that a TRIM protein, TRIM21, positively regulates protein synthesis by inhibiting the PKR-eIF2α signaling pathway in a ubiquitination-dependent manner, providing new evidence for the role of ubiquitination in stress-controlled protein homeostasis.

As the first step of protein synthesis, translation initiation is typically controlled by eIF2α, which is phosphorylated by four cellular kinases, PKR, GCN2, PERK and HRI, upon stress stimulation [3]. Among them, PKR is distinct for its activation by direct detection of the dsRNA upon viral infection. In mammalian cells, at least two signaling pathways are initiated upon detection of cytosolic dsRNA. One is the RLR signaling pathway, and the other is the PKR signaling pathway [23]. The TRIM family regulates RLR-mediated transcription of antiviral cytokines for a proper and efficient antiviral response has been widely studied and well clarified [37–39]. However, the role of the TRIM family in the regulation of antiviral effector translation remains poorly studied. In the present study, we demonstrate that the E3 ligase TRIM21 has the ability to promote protein synthesis of antiviral effectors by inactivating the PKR signaling pathway, which results in an antiviral response upon virus infection, which provides new insight into the TRIM family in the regulation of antiviral invasion.

TRIM21 is a member of the TRIM superfamily, which has been reported to be involved in diverse biological processes and implicated in various diseases [14]. Our previous study showed an important role of TRIM21 in the virus-triggered RLR signaling pathway [15]. Whether TRIM21 regulates the PKR signaling pathway remains elusive. In the present study, we found that TRIM21 negatively regulates PKR activation by promoting K6-linked ubiquitination of the PKR phosphatase PP1α. Several lines of evidence strongly support our conclusion. First, knocking out *TRIM21* augments stress-induced PKR activation and subsequent release of protein synthesis shutdown. Second, although TRIM21 has no effect on PKR ubiquitination, E3 ligase activity is essential for TRIM21-mediated inactivation of PKR, suggesting indirect regulation of PKR by TRIM21. Third, TRIM21 specifically abolishes PKR phosphorylation but not dsRNA detection or dimerization of PKR, indicating that the phosphatases of PKR may be regulated by TRIM21. Fourth, PP1α, the phosphatase of PKR, is responsible for TRIM21-mediated inhibition of PKR activation. TRIM21 promotes its interaction with PP1α and catalyzes the K6-linked ubiquitination of PP1α at Lys60 under stress. Fifth, silencing PP1α or K60 mutation of PP1α abolishes the inhibition of PKR phosphorylation by TRIM21. Collectively, our findings demonstrate that the TRIM21-PP1α axis is a newly discovered program for regulating stress-induced PKR-mediated protein synthesis. Moreover, we found an IFN-independent antiviral function of TRIM21. By performing proteomics analysis, several previously known and unknown antiviral effectors regulated by the TRIM21-PKR axis have been identified and proven to protect against viral infection, which broadens our understanding of antiviral genes and strengthens the evidence that the antiviral function of TRIM21 is achieved by reversing PKR-mediated translational shutdown.

As one of the classic posttranslational modifications (PTMs), ubiquitination profoundly affects fundamental physiological processes, such as cell proliferation, differentiation, cell death, and protein stability and structure, in all species [39,40]. E3 ligases are vital components in this process, directly interacting with and catalyzing different ubiquitin linkages of their specific substrates, which can occur through K6-, K11-, K27-, K29-, K33-, K48- and K63-linked ubiquitination [41]. Several types of ubiquitin linkages catalyzed by TRIM21 have been reported, and different ubiquitin linkages play distinct roles in host defense against pathogenic invasion [42]. For instance, TRIM21-mediated K48-linked ubiquitination is tightly related to proteasome-dependent degradation, which results in the degradation of viral proteins in virus-infected cells [43–45]. However, K48-linked ubiquitination of DDX41, one of the sensors detecting dsDNA, results in the inhibition of the innate immune response triggered by DNA virus [46]. K63-linked ubiquitination catalyzed by TRIM21 augments the activation of the intracellular antibody-triggered innate immune response [47]. K27-linked ubiquitination promotes RNA virus-induced MAVS activation and the innate immune response to viral infection [15]. Importantly, in this study, we find that TRIM21-catalyzed K6-linked ubiquitination of PP1α restricts viral infection by promoting the protein synthesis of intrinsic antiviral effectors. Thus, our findings indicate a newly discovered type of ubiquitin linkage mediated by TRIM21 that can participate in the host antiviral response with a distinct mechanism.

PKR activation suppresses the synthesis of interferon-stimulated genes (ISGs) upon viral infection [10,22]. Emerging evidence demonstrate the negative role of PKR-triggered protein inhibition in the host defense against viral infection. For example, one study reported that NLRX1 is a positive regulator of the antiviral response by limiting PKR-mediated translational shutoff of the IRF1 protein, which results in inhibition of the production of IRF1-dependent antiviral factors [48]. Similarly, our data show an IRF1-dependent antiviral efficiency regulated by TRIM21. TRIM21 promotes IRF1 protein synthesis by inhibiting PKR activation upon viral infection. In addition to TRIM21’s positive role in innate immunity, we define the antiviral role of TRIM21 as both augmenting the transcription of IFN production and facilitating translation of the IRF1 protein. In addition to its role in the innate antiviral response, IRF1 also plays a role in adaptive immunity and tumor suppression. Given that, we speculate that TRIM21 may have a role in restricting not only viral infection but also tumorigenesis via adaptive immunity, which needs further study. In addition, we also found an IRF1-independent antiviral function mediated by TRIM21 by a proteomics assay. Some previously unknown antiviral factors were discovered, all of which are directly regulated by TRIM21. Together with these findings, we demonstrate that an IFN-independent antiviral role of TRIM21 restricts viral infection by reversing PKR-mediated RNA translation inhibition of antiviral factors. Collectively, the results of our study highlight a newly discovered biological role of TRIM21, and these data provide new evidence of PKR-mediated translational arrest in host resistance to virus infection.

## Materials and methods

### Cell culture and reagents

The HLCZ01 cell line, a hepatoma cell line supporting the entire life cycle of HCV and HBV, was previously established in our laboratory. HEK293T cells were purchased from Boster, Huh7.5 cells were kindly provided by Charles M. Rice (Rockefeller University, New York), and Huh7 and A549 cells were obtained from the American Type Culture Collection. HLCZ01 cells were cultured in collagen-coated tissue culture plates containing Dulbecco’s modified Eagle medium (DMEM)–F-12 medium supplemented with 10% (vol/vol) fetal bovine serum (FBS) (Gibco), 40 ng/ml dexamethasone (Sigma), insulin-transferrin-selenium (ITS) (Lonza), and penicillin–streptomycin (Thermo Fisher Scientific). Other cells were propagated in DMEM supplemented with 10% FBS, nonessential amino acid solution (Thermo Fisher Scientific), and penicillin–streptomycin. Cell transfection with plasmids was conducted using ViaFect Transfection Reagent (Promega) or Lipofectamine 2000 (Thermo Fisher Scientific) in Opti-MEM medium (Thermo Fisher Scientific). For stable transfection or infection, monoclonal cells were screened using the antibiotic Puro (Gibco).

### Virus

The pJFH1 plasmid was a gift from Takaji Wakita (National Institute of Infectious Diseases, Tokyo, Japan). The linearized DNA from the pJFH1 plasmid was purified and used as the template for in vitro transcription with a MEGAscript kit (Ambion, Austin, TX). In vitro-transcribed genomic JFH1 RNA was delivered into Huh7.5 cells by electroporation. The transfected cells were cultured for the indicated periods. The cells were passaged every 3 to 5 days, while the corresponding supernatants were collected and filtered with a 0.45 μm filter device. The viral titers are presented as focus-forming units (FFUs) per milliliter, determined as the average number of NS5A-positive foci detected in Huh7.5 cells. VSV were kindly shared by Jianguo Wu (Jinan University, Guangzhhou, China), and SeV was kindly shared by Xingyi Ge (Hunan University, Changsha, China).

### Antibodies

Antibodies against the following proteins were obtained from commercial sources and used for immunoblots: anti-flag-tag (F3165, Sigma), anti-V5-tag (R960-25, Thermo Fisher Scientific), anti-puromycin (MABE343, Merck Millipore), anti-GAPDH (MAB374, Merck Millipore), anti-phospho-eIF2α Ser51 (3398, CST), anti-TRIM21 (92043, CST), anti-PKR (12297, CST), anti-Myc tag (2276, CST), anti-HA tag (ab236632, Abcam), anti-TRIM21 (ab91423, Abcam), anti-phospho-PKR T451 (ab81303, Abcam), and anti-IRF1 (8478, CST). Anti-interferon alpha/beta receptor 1 (ab45172, Abcam), anti-STAT2 (72604, CST), anti-PP1α (ab137512, Abcam), anti-ISG15 (ab285367, Abcam), anti-GST mouse monoclonal antibody (HT601-01, Transgen), β-actin (A5541, Sigma), and goat anti-mouse IgG (HRP-linked) (AP124P, Merck Millipore) were also used. The following antibodies were obtained from commercial sources and used for immunofluorescence staining: anti-puromycin (MABE343, Merck Millipore) and donkey anti-rabbit IgG (H + L) highly cross-adsorbed secondary antibody conjugated with Alexa Fluor 594 (A-21207, Thermo Fisher Scientific). The mouse monoclonal anti-HCV core antibody was a gift from Chen Liu.

### Plasmid construction

TRIM21, PP1α, and PKR cDNAs were synthesized from total cellular RNA isolated from HLCZ01 cells by standard reverse transcription-PCR (RT–PCR). Subsequently, they were cloned into the pcDNA3.1a vector, p3FLAG-CMV vector pCMV-N-Myc or pGEX4T2 vector. Multiple domains of TRIM21 and PP1α were amplified from the templates of full-length TRIM21 and PP1α, which were then cloned into p3xFLAG-CMV. The primers for amplifying these genes are listed in Table 1. The pHA-ub (K6, K11, K27, K29 and K33) plasmids were kindly shared by Hongbing Shu (Wuhan University). The pHA-ub (WT, K48 and K63) plasmids were kindly provided by Zhengfan Jiang (Peking University).

**Table 1:** Primers for the construction of plasmids.

GST-TRIM21 (F)	5’- CGGAATTCCCATGGCTTCAGCAGCACGCTTG-3’
GST-TRIM21 (R)	5’- ATAAGAATGCGGCCGCTCAATAGTCAGTGGATCC-3’
*PP1α* (F)	5’-GGGGTACCATGTCCGACAGCGAGAAGCTC-3’
*PP1α*-C (F)	5’-GGGGTACCATGAGGGGCAAGCAGTCCTTG-3’
*PP1α* (R)	5’-GCTCTAGATTTCTTGGCTTTGGCGGAATT-3’
*PP1α*-N (R)	5’-GCTCTAGAGTCCACATAGTCCCCCAGAAA-3’
GST-*PP1α* (F)	5’-CGGAATTCCCATGTCCGACAGCGAGAAGCTC-3’
GST-*PP1α* (R)	5’- ATAAGAATGCGGCCGCTCAATAGTCAGTGGATCC-3’
*PKR* (F)	5’-GGGGTACCATGGCTGGTGATCTTTCA-3’
*PKR* (R)	5’-GCTCTAGAACATGTGTGTCGTTAATTC-3’
Myc- PKR (F)	5’-CCCAAGCTTATGGCTGGTGATCTTTCA-3’
Myc- PKR (R)	5’-GGGGTACCACATGTGTGTCGTTCATT-3’
*eIF2α* (F)	5’-GGGGTACCATGCCGGGTCTAAGTTGTAGA-3’
*eIF2α* (R)	5’-GCTCTAGAATCTTCAGCTTTGGCTTCCAT-3’

### Lentivirus production and generation of stable cell lines

HEK293T cells plated in 10 cm dishes were transfected with 8 μg packaging plasmid psPAX2, 2.7 μg envelope plasmid pMD2G and 8 μg target plasmid encoding shRNA or lentiCRISPRv2 encoding sgRNA using Lipofectamine 2000 (Thermo Fisher Scientific). Lentivirus supernatants were collected at 36 h, 48 h, 56 h and 72 h post-transfection and clarified by filtration through 0.45 μm syringe filters. HLCZ01, A549 or Huh7.5 cells were plated in 6-well plates prior to being transduced with 500 μL per well of lentivirus supernatant. Transduced cells were selected for by the addition of 2 μg/mL puromycin at 72 h post-transduction. Loss of target protein expression was confirmed by Western blot. The target sequences used for shRNA gene silencing and lentiCRISPRv2 encoding sgRNA plasmids are listed in Table 2.

**Table 2:** Short hairpin RNAs used for knockdowns and single guide RNAs used for CRISPR/Cas9.

Target gene	Target sequence
sh-*FCGBP*	5’-GGGCTGTGTGGCAACTATAAT-3’
sh-*IRAK2*	5’-CCCACTTCGTCTGATTCAAAG-3’
sh-*PSMB9*	5’-CATCTACCTGGTCACTATTAC-3’
sh-*ROMO1*	5’-ATGGGCTTCGTGATGGGTTGC-3’
sh-*LXN*	5’-CCAGAAGTCAACTTCACATTT-3’
sh-*JADE1*	5’-GCCTGAGGAAGTAGTGGATTT-3’
sh-*RRM2B*	5’-GCGATGGATAGCAGATAGAAA-3’
sh-*CAPG*	5’-GCTGATATCTGATGACTGCTT-3’
sh-*NCAPH2*	5’-TACAGTAAGAAGGTGGAATAC-3’
sh-*TRIM21*	5’-GGCATGGTCTCCTTCTACAAC-3’
sh-*PP1α*	5’-GTGCAAGAGACGCTACAAC-3’
sh-*IRF3*	5’-GCCAACCTGGAAGAGGAAT-3’
sh-*STAT2*	5’-TGTCTTCTGCTTCCGATATAA-3’
sh-*IFR1*	5’-CGTGTGGATCTTGCCACAT-3’
sh-*PKR*	5’-GCTGAACTTCTTCATGTATGT-3’
sg-*TRIM21*	5’-GGAGCCTGTGAGCATCGAGTG-3’
sg-*IFNAR1*	5’-GCTGCGGACAACACCCA-3’
sg-*PKR*	5’-GATGGAAGAGAATTTCCAGA-3’

### Real-time PCR assay

Total cellular RNA was extracted by TRIzol reagent (Invitrogen) according to the manufacturer’s protocol. The Superscript III first-strand synthesis kit for reverse transcription of RNA was purchased from Invitrogen. After DNase (Promega) treatment, the extracted RNA was used as the template for reverse transcription-PCR. Real-time PCR was performed as described previously [15]. GAPDH was used as the internal control. The primers used for real-time PCR are listed in Table 3.

**Table 3:** Primers for Real-time PCR.

*GAPDH* (F)	5’-AATGGGCAGCCGTTAGGAAA-3’
*GAPDH* (R)	5’-GCGCCCAATACGACCAAATC-3’
VSV (F)	5’-CAAGTCAAAATGCCCAAGAGTCACA—3’
VSV (R)	5’-TTTCCTTGCATTGTTCTACAGATGG-3’
SeV (F)	5’-TGTTATCGGATTCCTCGACGCAGTC-3
SeV (R)	5’-TACTCTCCTCACCTGATCGATTATC-3
HCV (F)	5’-TCTGCGGAACCGGTGAGTA-3
HCV (R)	5’- TCAGGCAGTACCACAAGGC-3
H77-S (F)	5’-TCACTGCTTATGCCCAGCAA-3
H77-S (R)	5’-GCTCCGTGGTAAACAGTCCA-3
*TRIM21* (F)	5’-CCCCTCTAACCCTCTGTCCA-3’
*TRIM21* (R)	5’-GGGGAAAAGAGGCAGGGTTT-3’
*PSMB9* (F)	5’-GTGGATGCAGCATATAAGCC-3’
*PSMB9* (R)	5’-AGTGACCAGGTAGATGACAC-3’
*IRAK2* (F)	5’-CGCGTATCTGCCAGAGGATT-3’
*IRAK2* (R)	5’-AACCGGGCTTCGGTTGTTAT-3’
*FCGBP* (F)	5’-GTGTCTGCATCCCTGTCCAA-3’
*FCGBP* (R)	5’-GACAGGACACAGAGACCACG-3’
*ROMO1* (F)	5’-TTCGACCGTGTCAAAATGGG-3’
*ROMO1* (R)	5’-GCCACTCTGCATCATGGTTT-3’
*LXN* (F)	5’-AAACAAGCCAGCATGGAGGATA-3’
*LXN* (R)	5’-TCAGCTGTGCAGTTCACCTT-3’
*JADE1* (F)	5’-TGGGTTCTCGATCTGTAGCG-3’
*JADE1* (R)	5’-ACAGCAGGCAGCTGATCCAA-3’
*RRM2B* (F)	5’-ATGTTATTCGCCGCGGTCAG-3’
*RRM2B* (R)	5’-TGAAGATGATCTCCCGGCCT-3’
*CAPG* (F)	5’-GGAAGGTGGTGTGGAGTCAG-3’
*CAPG* (R)	5’-ACCAGGCGAAGATGTTCTGG-3’
*NCAPH2* (F)	5’-GGGGGCAGCAGATGACTTT-3’
*NCAPH2* (R)	5’-TCCTCGTAGCTCAGGGACAT-3’

### Western blotting

Cells were washed with PBS and lysed with RIPA buffer (Thermo Fisher Scientific) supplemented with protease inhibitor cocktail (Thermo Fisher Scientific). The lysates were incubated on ice for 30 min and centrifuged at 16000 g for 15 min at 4°C. Proteins were resolved on SDS–PAGE gels and transferred to polyvinylidene difluoride (PVDF) membranes (Merck Millipore). The PVDF membranes were then blocked with 5% skim milk and sequentially incubated with primary and secondary antibodies. The bound antibodies were detected using SuperSignal West Pico chemiluminescent substrate (Pierce, Rockford, IL).

### IP and immunoblotting

Cells were washed with PBS and lysed with IP Lysis Buffer (Thermo Fisher Scientific) supplemented with protease inhibitor cocktail. The lysates were incubated on ice for 30 min and centrifuged at 16000 g for 15 min at 4°C. The lysates were diluted to a concentration of 2 μg/μL with PBS before IP. The lysates (200 μg) were immunoprecipitated with the indicated antibodies. The immunocomplex was captured by adding Protein G Agarose (Merck Millipore, Darmstadt, Germany). The protein binding to the beads was boiled in 2 x Laemmli sample buffer (Bio–Rad, Hercules) and was then subjected to SDS–PAGE.

### RNA immunoprecipitation

The cells were trypsinized to detach them, and the supernatant was discarded. The cells were washed by gently resuspending them in 1 ml PBS and pelleted by centrifugation at 3,000 g for 1 min. Then, the cells were resuspended in 1 mL 1% formaldehyde (diluted in PBS) and allowed to stand for 10 min at room temperature. The cells were pelleted by centrifugation at 3,000 g for 1 min, resuspended in 1 mL 0.25 M glycine solution (diluted in PBS) and again allowed to stand for 10 min at room temperature. The cells were pelleted and washed with 500 μL PBS, and then the cells were lysed with RIPA buffer supplemented with a protease inhibitor cocktail and an RNase inhibitor on ice for 30 min. The subsequent immunoprecipitation was processed as described above. Protein–RNA complexes binding to beads were eluted in PBS at 70°C for 45 min. The eluted material was lysed in ice-cold TRIzol reagent for RT–PCR.

### Immunofluorescence staining

Cells were seeded into a confocal dish and fixed with 4% paraformaldehyde for 15 min at room temperature. The cells were washed with PBS, permeabilized for 15 min with 0.2% Triton X-100 in PBS for 10 min, blocked with goat serum (Thermo Fisher Scientific) for 30 min at room temperature, and sequentially incubated with primary and fluorescence-labeled secondary antibodies (Invitrogen) (diluted in PBS to 1:500) at room temperature for 2 h. Nuclei were counterstained with DAPI (Vector Laboratories, Burlingame) for 5 min. Images were captured using a TI-E + A1 SI confocal microscope (Nikon).

### Plaque assay

The supernatants were diluted into multiple concentrate gradients with medium without FBS and transduced into VERO cells. After 1 h, the cells were placed in complete medium. Two hours later, 1% agarose media was added on top of the virus-infected Vero cells. After 1 day, cells with plaques were fixed with 4% paraformaldehyde for 30 min at room temperature and stained with 0.1% crystal violet.

### Puromycin incorporation assay

Cells were pulsed with puromycin (10 μg/mL) for 1 h and then fixed with 4% paraformaldehyde, followed by staining with anti-puromycin and DAPI. Cells were imaged by confocal microscopy. Alternatively, cells were pulsed with puromycin (10 μg/mL) for 1 h, lysed, and puromycin-labeled proteins were identified by immunoblot analysis.

### Statistical analysis

All results are presented as the means and standard deviations (SDs). Comparisons between two groups were performed by using Student’s t test.

## Supporting information

S1 Fig TRIM21 inhibits the activation of PKR. (A) Immunoblot analysis of TRIM21 in wild-type A549 cells (sg-vector) and TRIM21-deficient A549 cells (sg-TRIM21). GAPDH was detected as an internal control. (B) TRIM21 was assessed by immunoblot in A549 cells infected with Lentivirus-sh-vector (sh-vector) or Lentivirus-sh-TRIM21 (sh-TRIM21) for 72 h. GAPDH was detected as an internal control. (C) A549 cells were infected with Lentivirus-sh-vector (sh-vector) or Lentivirus-sh-TRIM21 (sh-TRIM21) for 48 h, then transfected with poly (I: C) (1 μg or 2 μg) for 12 h. The indicated proteins were detected by western blot. β-actin was used as an internal control. (D-E) A549 cells were infected with Lentivirus-sh-vector (sh-vector) or Lentivirus-sh-TRIM21 (sh-TRIM21) for 48 h, then infected by VSV (MOI = 0.2 or 0.5) for 6 h (D) or SeV (MOI = 0.2 or 0.5) for 12 h (E). The indicated proteins were detected by western blot. β-actin was used as an internal control. (F) HLCZ01 cells were infected with Lentivirus-sh-vector (sh-vector) or Lentivirus-sh-TRIM21 (sh-TRIM21) for 48 h, then transfected with poly (I: C) (1 μg or 2 μg) for 12 h. The indicated proteins were detected by western blot. β-actin was used as an internal control. (G-H) HLCZ01 cells were infected with Lentivirus-sh-vector (sh-vector) or Lentivirus-sh-TRIM21 (sh-TRIM21) for 48 h, then infected by VSV (MOI = 0.2 or 0.5) for 6 h (G) or SeV (MOI = 0.2 or 0.5) for 12 h (H). The indicated proteins were detected by western blot. β-actin was used as an internal control. (I) Immunoblot analysis of PKR in wild-type A549 cells (sg-vector) and PKR-deficient A549 cells (sg-PKR). GAPDH was detected as an internal control. The relative ratios of p-PKR or p-eIF2α in (C-H) were quantified by densiometric analysis, which were normalized to the value in the control group. Experiments were independently repeated two or three time with similar results, and the data shown are mean ± SD. P values were determined by Student’s t-test. *p<0.05, **p<0.01, ***p<0.001.
(TIF)

S2 Fig TRIM21 inhibits PKR signaling pathway activation via PP1α. (A) Immunoblot analysis of PP1α protein in wild-type A549 cells (sh-vector) or PP1α-silenced A549 cells (sh-PP1α). β-actin was detected as an internal control. (B-C) PP1α-silenced A549 cells (sh-PP1α) pre-infected with Lentivirus-Flag-PP1α-WT or Lentivirus-Flag-PP1α-H248K for 24 h were infected with Lentivirus-sh-TRIM21 (sh-TRIM21) for 48 h, then infected with VSV (MOI = 0.2 or 0.5) for 6 h (B) or stimulated with TG (10 μM) for 12 h (C). The indicated proteins were detected by western blot and β-actin was used as an internal control. (D-E) A549 cells were infected with Lentivirus-sh-vector (sh-vector) or Lentivirus-sh-TRIM21 (sh-TRIM21) for 48 h, then infected with VSV (MOI = 0.2) for indicated times (D), or VSV (MOI = 0.2 or 0.5) for 6 h (E). The indicated proteins were analyzed by western blot. GAPDH was used as an internal control. (F) A549 cells were infected with Lentivirus-sh-vector (sh-vector) or Lentivirus-sh-TRIM21 (sh-TRIM21) for 48 h, then stimulated with TG (10 μM or 20 μM) for 12 h. The indicated proteins were analyzed by western blot. GAPDH was used as an internal control. The relative ratios of p-PKR in (B-C) were quantified by densiometric analysis, which were normalized to the value in the control group. Experiments were independently repeated two or three time with similar results, and the data shown are mean ± SD. P values were determined by Student’s t-test. *p<0.05, **p<0.01, NS, no significance difference.
(TIF)

S3 Fig TRIM21 restricts viral infection by inhibiting PKR signaling pathway activation. (A) Immunoblot analysis of IFNAR1 in wild-type A549 cells or HLCZ01 cells (sg-vector) or IFNAR1-deficient A549 or HLCZ01 cells (sg-IFNAR1). GAPDH was detected as an internal control. (B) IFNAR1-deficient A549 cells (sg-IFNAR1) were infected with Lentivirus-sh-vector (sh-vector) or Lentivirus-sh-TRIM21 (sh-TRIM21) for 48 h, then infected with SeV (MOI = 0.5) for 12 h. The RNA level of SeV was analyzed by RT- qPCR. (C) Immunoblot analysis of the STAT2 protein in STAT2-wild-type A549-sg-IFNAR1 cells (sh-vector) or STAT2-silenced A549-sg-IFNAR1 cells (sh-STAT2). β-actin was detected as an internal control. (D) Immunoblot analysis of IRF3 protein in wild-type HLCZ01 cells (sh-vector) and IRF3-silenced HLCZ01 cells (sh-IRF3). β-actin was used as an internal control. (E) Huh7.5 cells were infected with Lentivirus-sh-vector (sh-vector) or Lentivirus-sh-TRIM21 (sh-TRIM21) for 48 h, then infected with SeV (MOI = 0.5) for 12 h. The RNA level of SeV was analyzed by RT-qPCR. (F) Immunoblot analysis of the protein levels of PKR and IFNAR1 in wild-type A549 cells (sg-vector) and PKR/IFNAR1 double-deficient A549 cells (sg-PKR/IFNAR1). β-actin was used as an internal control. (G) Immunoblot analysis of the expression of PKR in wild-type Huh7.5 cells (sg-vector) and PKR-deficient Huh7.5 cells (sg-PKR). GAPDH was used as an internal control. (H) Wild-type (sg-vector) or PKR-deficient Huh7.5 cells (sg-PKR) were infected with Lentivirus-sh-vector (sh-vector) or Lentivirus-sh-TRIM21 (sh-TRIM21) for 48 h, then infected with SeV (MOI = 0.2) for 12 h. The RNA level of SeV was analyzed by RT-qPCR. (I) Immunoblot analysis of the expression of PKR and TRIM21 in wild-type Huh7.5 cells (sg-vector) and PKR/TRIM21 double-deficient Huh7.5 cells (sg-PKR/TRIM21). β-actin was used as an internal control. (J) PKR/TRIM21 double-deficient Huh7.5 cells were infected with Lentivirus-Flag-vector or Lentivirus-Flag-TRIM21 for 48 h, then infected with VSV (MOI = 0.2 or 0.5) for 6 h, or SeV (MOI = 0.2 or 0.5) for 12 h. The viral RNA levels were examined by RT-qPCR. Experiments were independently repeated two or three time with similar results, and the data shown are mean ± SD. P values were determined by Student’s t-test. *p<0.05, **p<0.01, ***p<0.001, NS, no significance difference. ND, not detected.
(TIF)

S4 Fig TRIM21 restricts HCV replication in response to IFN. (A-D) Huh7.5 cells per-infected with HCV (JFH-1 strain) (MOI = 0.1) for 24 h were infected with Lentivirus-sh-vector (sh-vector) or Lentivirus-sh-TRIM21 (sh-TRIM21) for 48 h, followed by IFN-α (100 U/ mL or 500 U/mL) treatment for 24 h. The indicated proteins were examined by western blot. β-actin was used as an internal control (A). The levels of HCV RNA were analyzed by RT-qPCR (B). NS3 protein was analyzed by western blot (C). The levels of HCV particles in the supernatant were analyzed by immunofluorescence (D). The relative ratios of p-PKR or p-eIF2α in (A) and the relative amounts of HCV NS3 proteins in (C) were quantified by densiometric analysis, which were normalized to the value in the control group with two or three repeats. The data shown are mean ± SD. P values were determined by Student’s t-test. *p<0.05, **P<0.01, ***P<0.001. (E) Huh7.5 cells pre-infected with HCV (H77-S strain) (MOI = 0.1) for 24 h were infected with Lentivirus-sh-vector (sh-vector) or Lentivirus-sh-TRIM21 (sh-TRIM21) for 48 h, followed by IFN-α (500 U/mL) treatment for 24 h. Viral RNA was quantified by real-time PCR. (F) Immunoblot analysis of the protein levels of TRIM21 or IFNAR1 in wild-type A549 cells, TRIM21-deficient A549 cells, IFNAR1-deficient A549 cells or IFNAR1/TRIM21 double-deficient A549 cells. GAPDH was used as an internal control. (G) IFNAR1/TRIM21 double-deficient A549 cells (sg-IFNAR1/TRIM21) were infected with Lentivirus-Flag-vector or Lentivirus-Flag-TRIM21 for 48 h, then infected with VSV (MOI = 0.2 or 0.5) for 6 h. Puromycin incorporation assays of the cellular protein synthesis. GAPDH was used as an internal control. The relative ratios of puro signals were quantified by densiometric analysis, which were normalized to the value in the control group with two or three repeats. The data shown are mean ± SD. P values were determined by Student’s t-test. *p<0.05. (H) IFNAR1/TRIM21 double-deficient A549 cells were infected with Lentivirus-Flag-vector or Lentivirus-Flag-TRIM21 for 48 h, then infected with VSV (MOI = 0.2) for 6 h. Cells were pulse-labeled with puromycin (10 μg/mL) for 1 h prior to fixation. Immunofluorescence staining for puromycin in the treated cells. The nuclei were stained with DAPI. Experiments were independently repeated two or three time with similar results. Student’s two-sided t test, and the data are represented as mean ± SD. *p<0.05 versus the control, **p<0.01 versus the control, ***p<0.001 versus the control.
(TIF)

S1 File Raw data for proteome analysis. (XLSX)

S2 File Raw data for western blot. (PDF)
