# Supplementary figures and images for: Regulation of PKR-dependent RNA translation inhibition by TRIM21 upon virus infection or other stress
Source: PLoS Pathog. 2023 Jun 16;19(6):e1011443. doi: 10.1371/journal.ppat.1011443 (PMC10310049; doi:10.1371/journal.ppat.1011443)

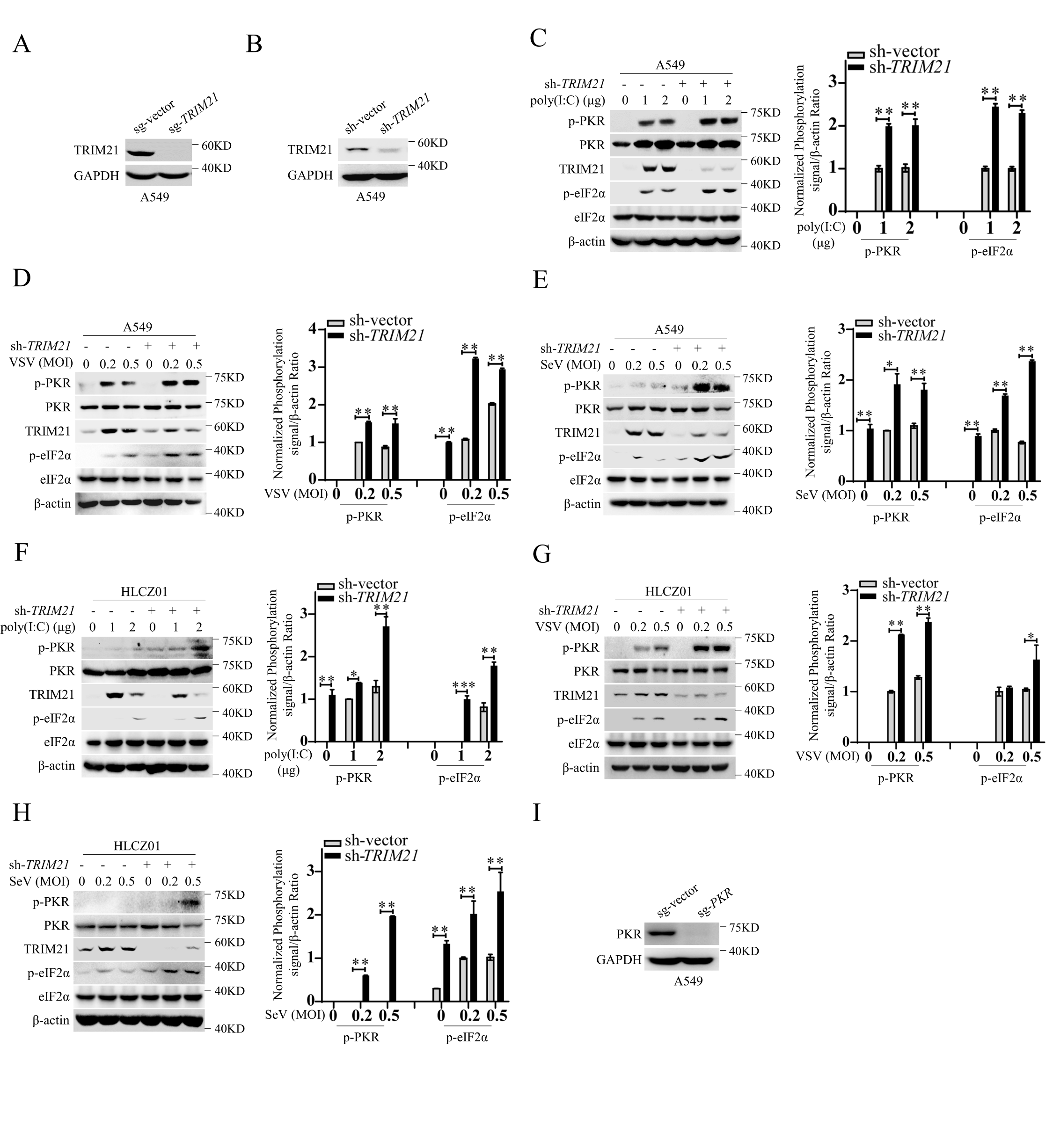

Supplement: S1 Fig — (A) Immunoblot analysis of TRIM21 in wild-type A549 cells (sg-vector) and TRIM21-deficient A549 cells (sg-TRIM21). GAPDH was detected as an internal control. (B) TRIM21 was assessed by immunoblot in A549 cells infected with Lentivirus-sh-vector (sh-vector) or Lentivirus-sh-TRIM21 (sh-TRIM21) for 72 h. GAPDH was detected as an internal control. (C) A549 cells were infected with Lentivirus-sh-vector (sh-vector) or Lentivirus-sh-TRIM21 (sh-TRIM21) for 48 h, then transfected with poly (I: C) (1 μg or 2 μg) for 12 h. The indicated proteins were detected by western blot. β-actin was used as an internal control. (D-E) A549 cells were infected with Lentivirus-sh-vector (sh-vector) or Lentivirus-sh-TRIM21 (sh-TRIM21) for 48 h, then infected by VSV (MOI = 0.2 or 0.5) for 6 h (D) or SeV (MOI = 0.2 or 0.5) for 12 h (E). The indicated proteins were detected by western blot. β-actin was used as an internal control. (F) HLCZ01 cells were infected with Lentivirus-sh-vector (sh-vector) or Lentivirus-sh-TRIM21 (sh-TRIM21) for 48 h, then transfected with poly (I: C) (1 μg or 2 μg) for 12 h. The indicated proteins were detected by western blot. β-actin was used as an internal control. (G-H) HLCZ01 cells were infected with Lentivirus-sh-vector (sh-vector) or Lentivirus-sh-TRIM21 (sh-TRIM21) for 48 h, then infected by VSV (MOI = 0.2 or 0.5) for 6 h (G) or SeV (MOI = 0.2 or 0.5) for 12 h (H). The indicated proteins were detected by western blot. β-actin was used as an internal control. (I) Immunoblot analysis of PKR in wild-type A549 cells (sg-vector) and PKR-deficient A549 cells (sg-PKR). GAPDH was detected as an internal control. The relative ratios of p-PKR or p-eIF2α in (C-H) were quantified by densiometric analysis, which were normalized to the value in the control group. Experiments were independently repeated two or three time with similar results, and the data shown are mean ± SD. P values were determined by Student’s t-test. *p<0.05, **p<0.01, ***p<0.001. (TIF) [file ppat.1011443.s001.tif]

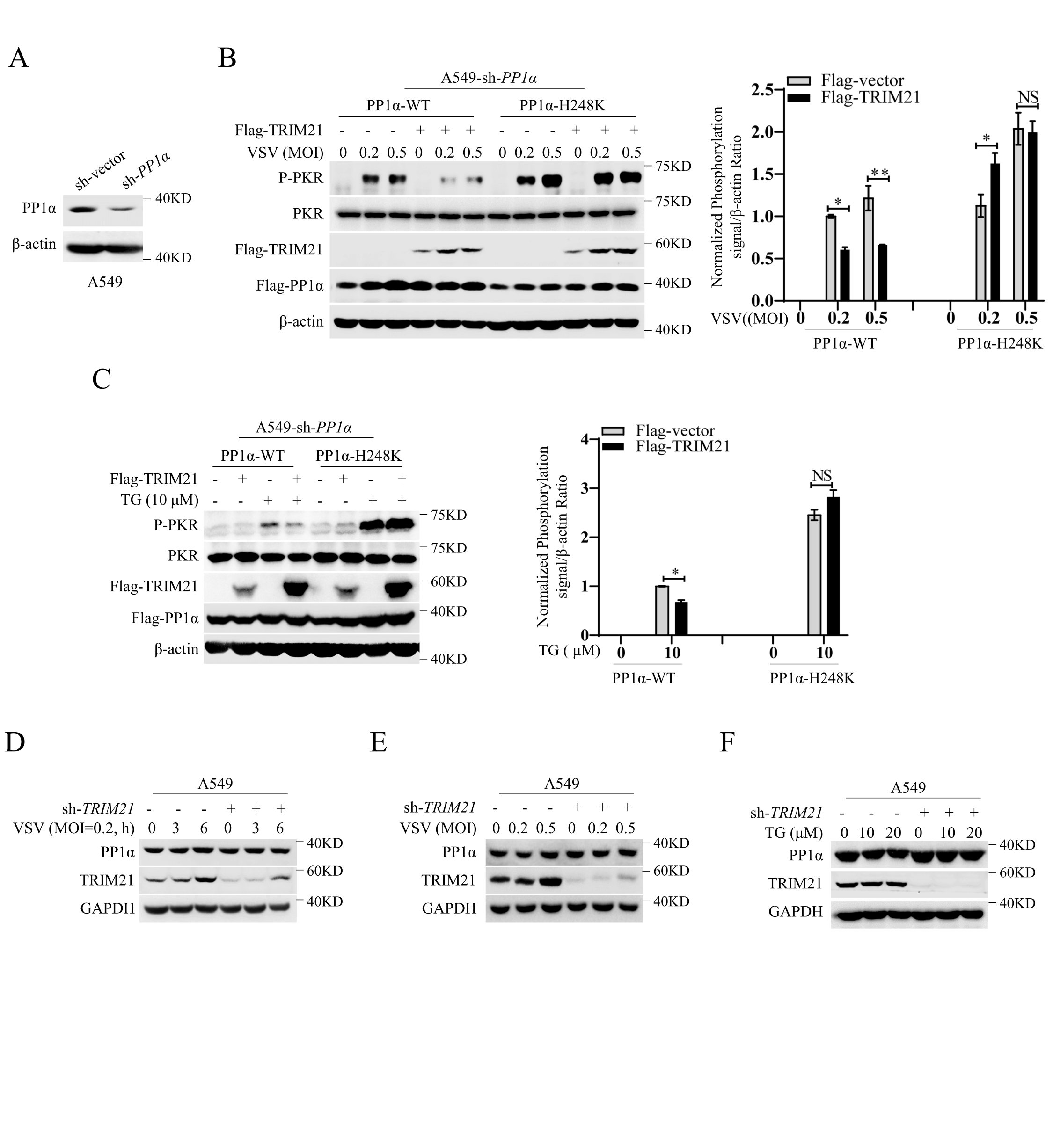

Supplement: S2 Fig — (A) Immunoblot analysis of PP1α protein in wild-type A549 cells (sh-vector) or PP1α-silenced A549 cells (sh-PP1α). β-actin was detected as an internal control. (B-C) PP1α-silenced A549 cells (sh-PP1α) pre-infected with Lentivirus-Flag-PP1α-WT or Lentivirus-Flag-PP1α-H248K for 24 h were infected with Lentivirus-sh-TRIM21 (sh-TRIM21) for 48 h, then infected with VSV (MOI = 0.2 or 0.5) for 6 h (B) or stimulated with TG (10 μM) for 12 h (C). The indicated proteins were detected by western blot and β-actin was used as an internal control. (D-E) A549 cells were infected with Lentivirus-sh-vector (sh-vector) or Lentivirus-sh-TRIM21 (sh-TRIM21) for 48 h, then infected with VSV (MOI = 0.2) for indicated times (D), or VSV (MOI = 0.2 or 0.5) for 6 h (E). The indicated proteins were analyzed by western blot. GAPDH was used as an internal control. (F) A549 cells were infected with Lentivirus-sh-vector (sh-vector) or Lentivirus-sh-TRIM21 (sh-TRIM21) for 48 h, then stimulated with TG (10 μM or 20 μM) for 12 h. The indicated proteins were analyzed by western blot. GAPDH was used as an internal control. The relative ratios of p-PKR in (B-C) were quantified by densiometric analysis, which were normalized to the value in the control group. Experiments were independently repeated two or three time with similar results, and the data shown are mean ± SD. P values were determined by Student’s t-test. *p<0.05, **p<0.01, NS, no significance difference. (TIF) [file ppat.1011443.s002.tif]

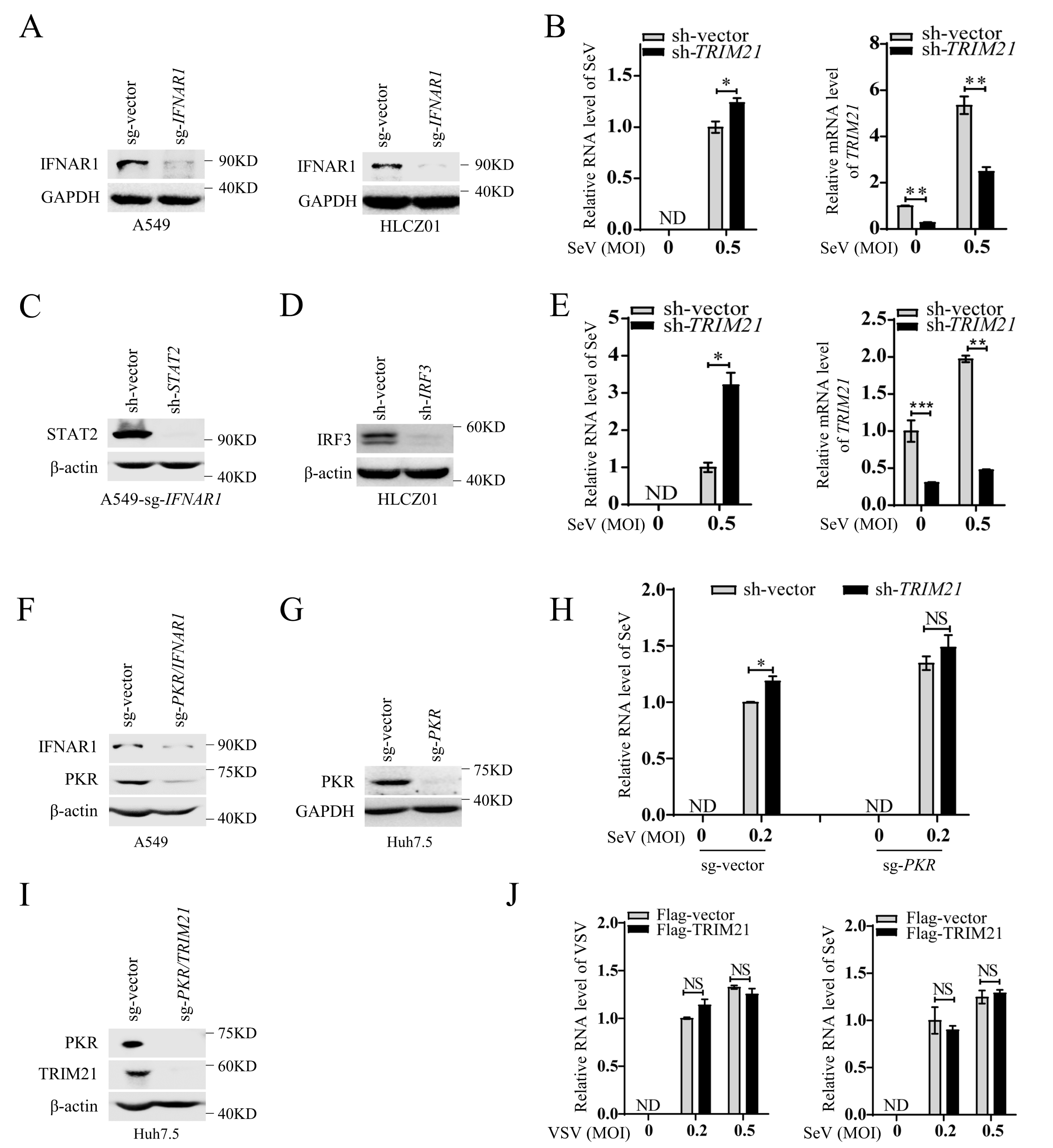

Supplement: S3 Fig — (A) Immunoblot analysis of IFNAR1 in wild-type A549 cells or HLCZ01 cells (sg-vector) or IFNAR1-deficient A549 or HLCZ01 cells (sg-IFNAR1). GAPDH was detected as an internal control. (B) IFNAR1-deficient A549 cells (sg-IFNAR1) were infected with Lentivirus-sh-vector (sh-vector) or Lentivirus-sh-TRIM21 (sh-TRIM21) for 48 h, then infected with SeV (MOI = 0.5) for 12 h. The RNA level of SeV was analyzed by RT- qPCR. (C) Immunoblot analysis of the STAT2 protein in STAT2-wild-type A549-sg-IFNAR1 cells (sh-vector) or STAT2-silenced A549-sg-IFNAR1 cells (sh-STAT2). β-actin was detected as an internal control. (D) Immunoblot analysis of IRF3 protein in wild-type HLCZ01 cells (sh-vector) and IRF3-silenced HLCZ01 cells (sh-IRF3). β-actin was used as an internal control. (E) Huh7.5 cells were infected with Lentivirus-sh-vector (sh-vector) or Lentivirus-sh-TRIM21 (sh-TRIM21) for 48 h, then infected with SeV (MOI = 0.5) for 12 h. The RNA level of SeV was analyzed by RT-qPCR. (F) Immunoblot analysis of the protein levels of PKR and IFNAR1 in wild-type A549 cells (sg-vector) and PKR/IFNAR1 double-deficient A549 cells (sg-PKR/IFNAR1). β-actin was used as an internal control. (G) Immunoblot analysis of the expression of PKR in wild-type Huh7.5 cells (sg-vector) and PKR-deficient Huh7.5 cells (sg-PKR). GAPDH was used as an internal control. (H) Wild-type (sg-vector) or PKR-deficient Huh7.5 cells (sg-PKR) were infected with Lentivirus-sh-vector (sh-vector) or Lentivirus-sh-TRIM21 (sh-TRIM21) for 48 h, then infected with SeV (MOI = 0.2) for 12 h. The RNA level of SeV was analyzed by RT-qPCR. (I) Immunoblot analysis of the expression of PKR and TRIM21 in wild-type Huh7.5 cells (sg-vector) and PKR/TRIM21 double-deficient Huh7.5 cells (sg-PKR/TRIM21). β-actin was used as an internal control. (J) PKR/TRIM21 double-deficient Huh7.5 cells were infected with Lentivirus-Flag-vector or Lentivirus-Flag-TRIM21 for 48 h, then infected with VSV (MOI = 0.2 or 0.5) for 6 h, or SeV (MOI = 0.2 or 0.5) [file ppat.1011443.s003.tif]

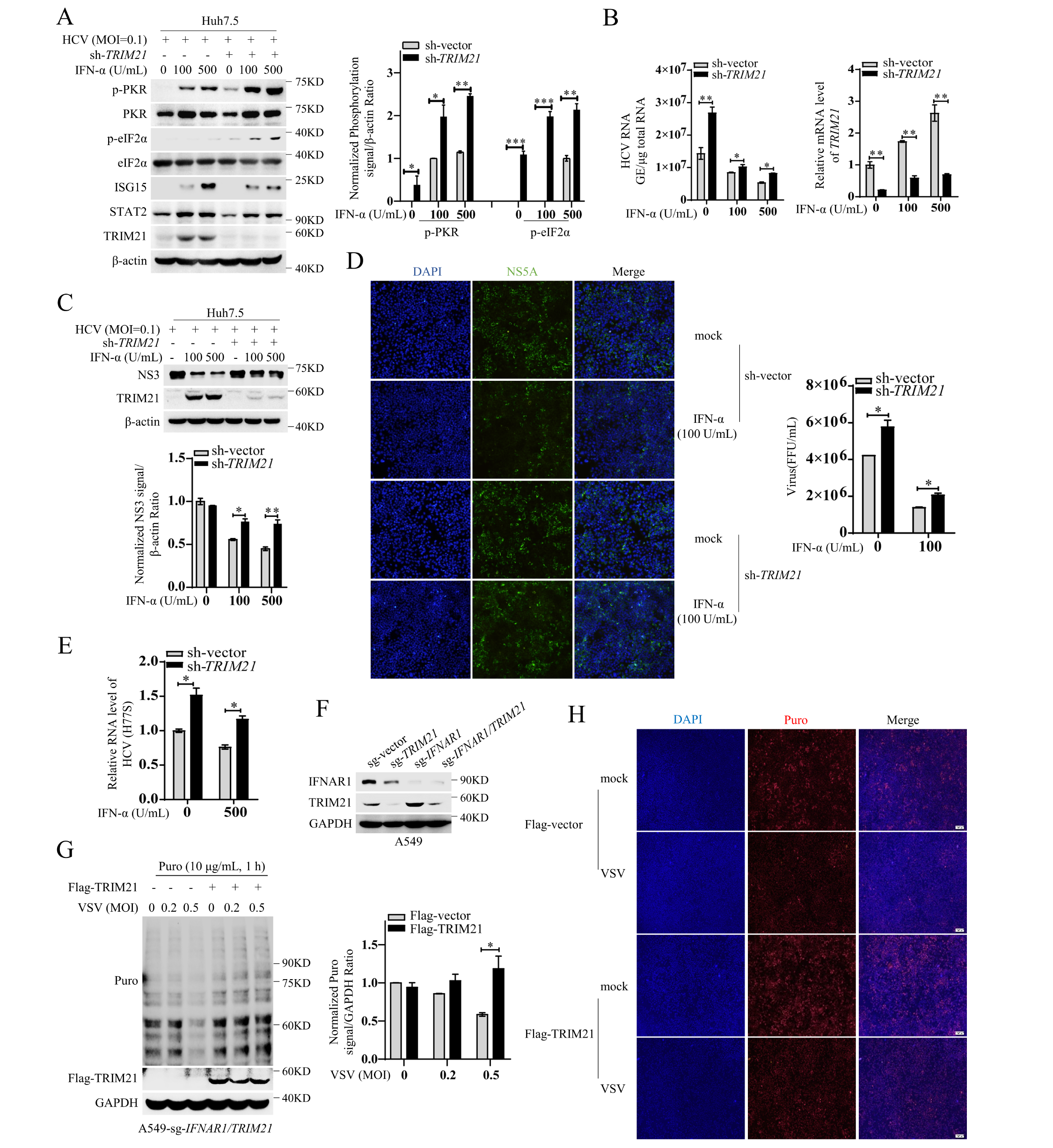

Supplement: S4 Fig — (A-D) Huh7.5 cells per-infected with HCV (JFH-1 strain) (MOI = 0.1) for 24 h were infected with Lentivirus-sh-vector (sh-vector) or Lentivirus-sh-TRIM21 (sh-TRIM21) for 48 h, followed by IFN-α (100 U/ mL or 500 U/mL) treatment for 24 h. The indicated proteins were examined by western blot. β-actin was used as an internal control (A). The levels of HCV RNA were analyzed by RT-qPCR (B). NS3 protein was analyzed by western blot (C). The levels of HCV particles in the supernatant were analyzed by immunofluorescence (D). The relative ratios of p-PKR or p-eIF2α in (A) and the relative amounts of HCV NS3 proteins in (C) were quantified by densiometric analysis, which were normalized to the value in the control group with two or three repeats. The data shown are mean ± SD. P values were determined by Student’s t-test. *p<0.05, **P<0.01, ***P<0.001. (E) Huh7.5 cells pre-infected with HCV (H77-S strain) (MOI = 0.1) for 24 h were infected with Lentivirus-sh-vector (sh-vector) or Lentivirus-sh-TRIM21 (sh-TRIM21) for 48 h, followed by IFN-α (500 U/mL) treatment for 24 h. Viral RNA was quantified by real-time PCR. (F) Immunoblot analysis of the protein levels of TRIM21 or IFNAR1 in wild-type A549 cells, TRIM21-deficient A549 cells, IFNAR1-deficient A549 cells or IFNAR1/TRIM21 double-deficient A549 cells. GAPDH was used as an internal control. (G) IFNAR1/TRIM21 double-deficient A549 cells (sg-IFNAR1/TRIM21) were infected with Lentivirus-Flag-vector or Lentivirus-Flag-TRIM21 for 48 h, then infected with VSV (MOI = 0.2 or 0.5) for 6 h. Puromycin incorporation assays of the cellular protein synthesis. GAPDH was used as an internal control. The relative ratios of puro signals were quantified by densiometric analysis, which were normalized to the value in the control group with two or three repeats. The data shown are mean ± SD. P values were determined by Student’s t-test. *p<0.05. (H) IFNAR1/TRIM21 double-deficient A549 cells were infected with Lentivirus-Flag-vector or Lentivirus-Fl [file ppat.1011443.s004.tif]
