# Supplementary material for: Regulation of PKR-dependent RNA translation inhibition by TRIM21 upon virus infection or other stress
Source: PLoS Pathog. 2023 Jun 16;19(6):e1011443. doi: 10.1371/journal.ppat.1011443 (PMC10310049; doi:10.1371/journal.ppat.1011443)
Supplement: S2 File — (PDF) [file ppat.1011443.s006.pdf]

Supporting Information-raw data for western blot

Figure 1

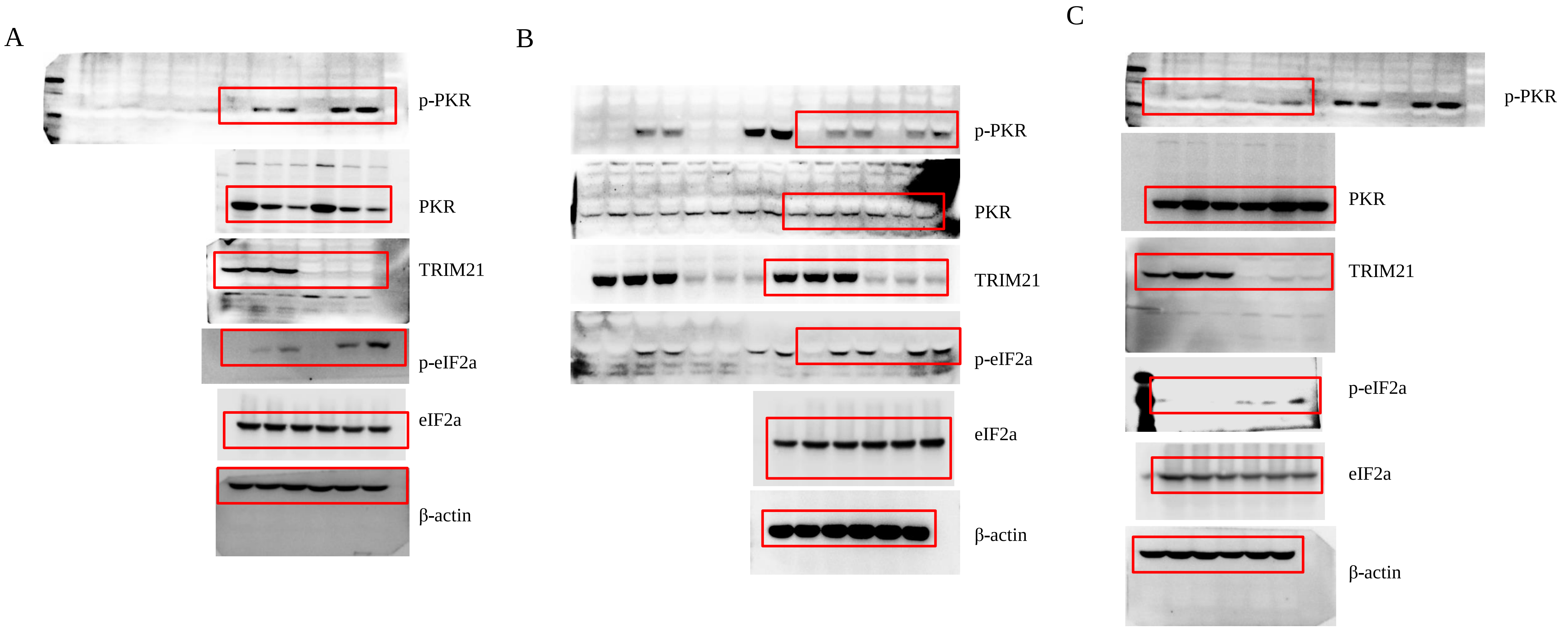

A
p-PKR
PKR
TRIM21
p-eIF2a
eIF2a
β-actin
B
p-PKR
PKR
TRIM21
p-eIF2a
eIF2a
β-actin
C
p-PKR
PKR
TRIM21
p-eIF2a
eIF2a
β-actin

D

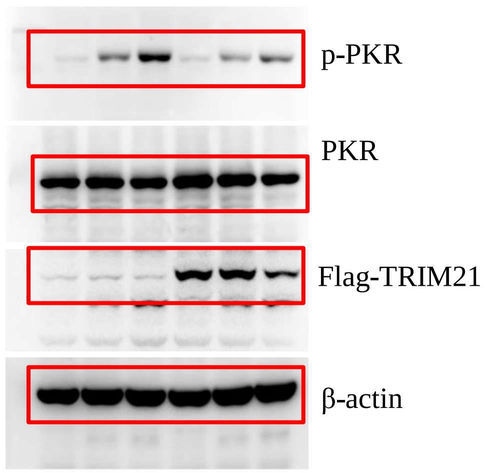
p-PKR
PKR
Flag-TRIM21
β-actin

E

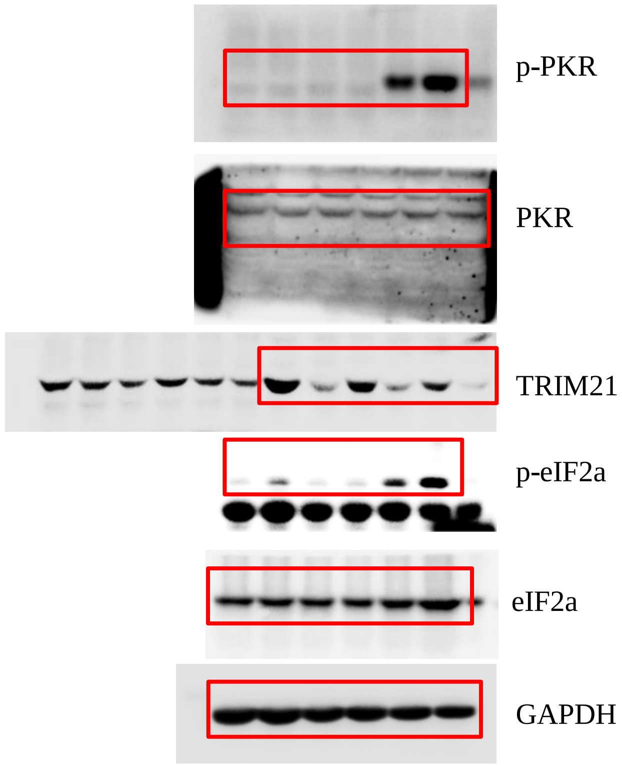
p-PKR
PKR
TRIM21
p-eIF2a
eIF2a
GAPDH

F

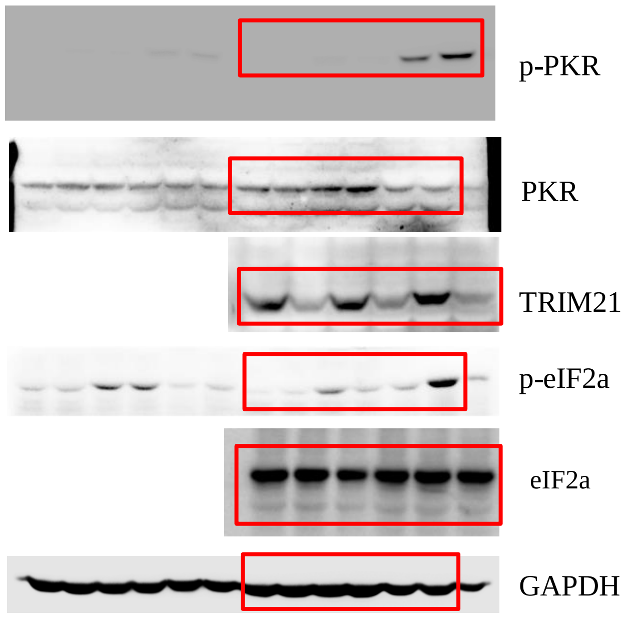
p-PKR
PKR
TRIM21
p-eIF2a
eIF2a
GAPDH

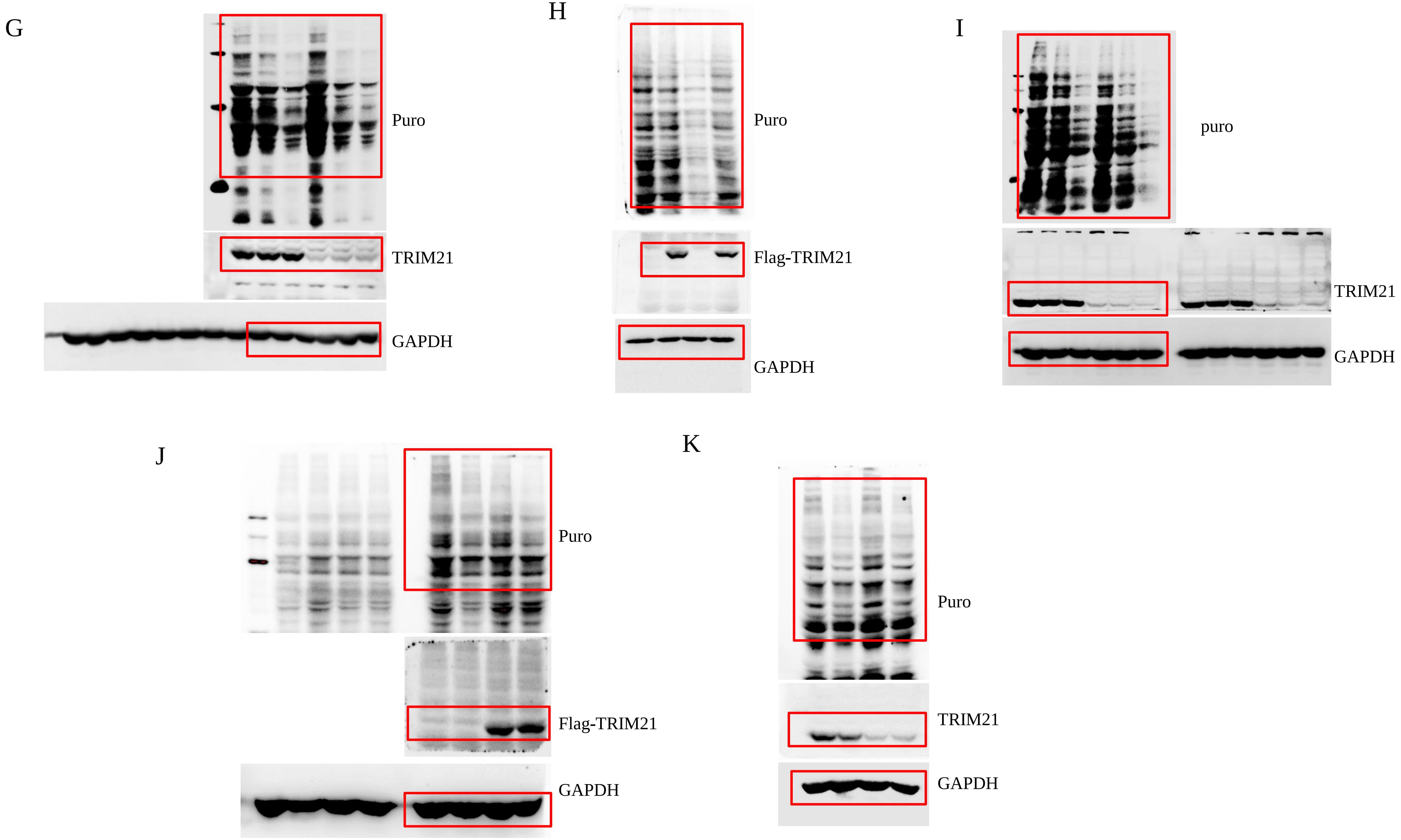
G
Puro
TRIM21
GAPDH
H
Puro
Flag-TRIM21
GAPDH
I
puro
TRIM21
GAPDH
J
Puro
Flag-TRIM21
GAPDH
K
Puro
TRIM21
GAPDH

Figure 2

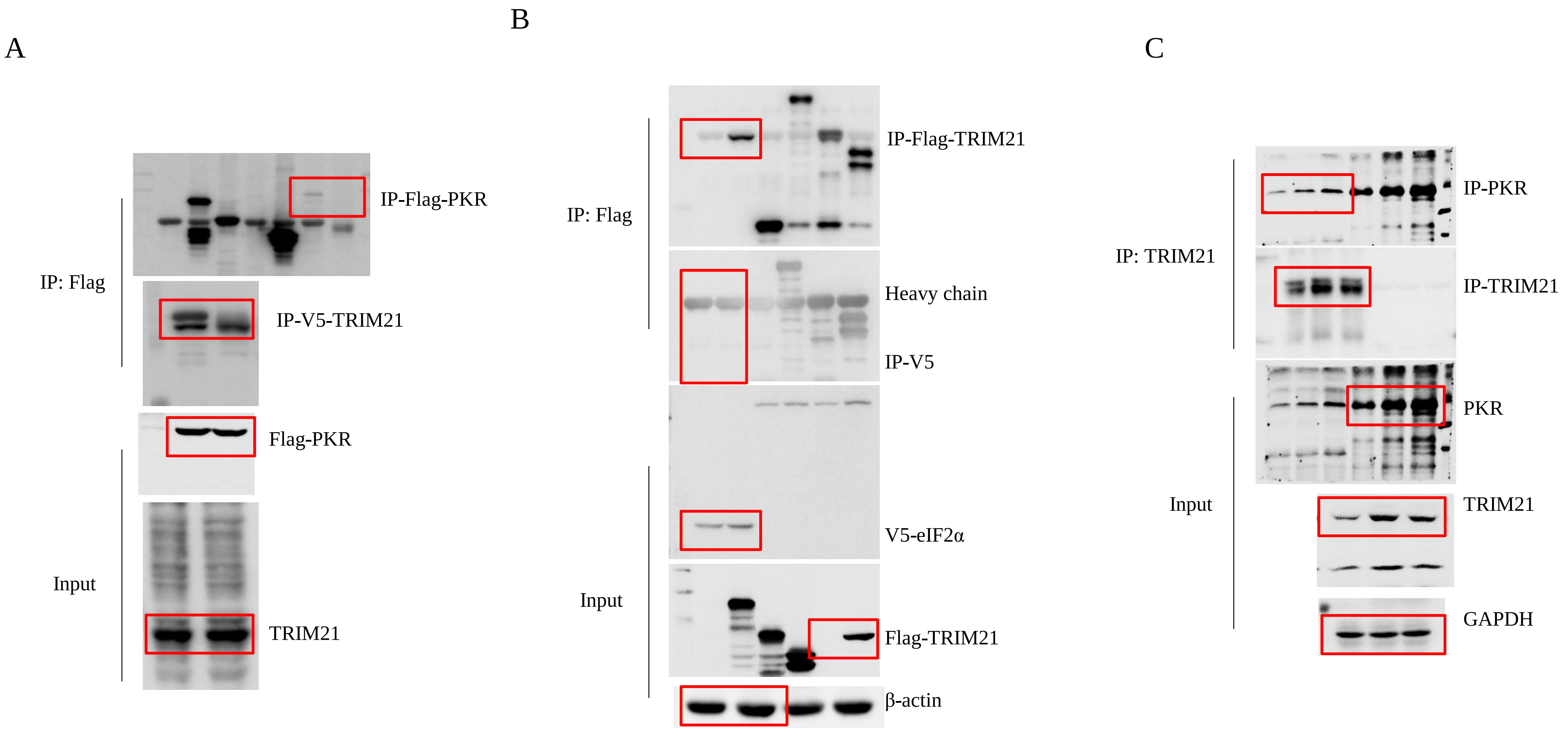
A
IP: Flag
IP-Flag-PKR
IP-V5-TRIM21
Flag-PKR
Input
TRIM21
B
IP: Flag
IP-Flag-TRIM21
Heavy chain
IP-V5
V5-eIF2α
Input
Flag-TRIM21
β-actin
C
IP: TRIM21
IP-PKR
IP-TRIM21
PKR
Input
TRIM21
GAPDH

D

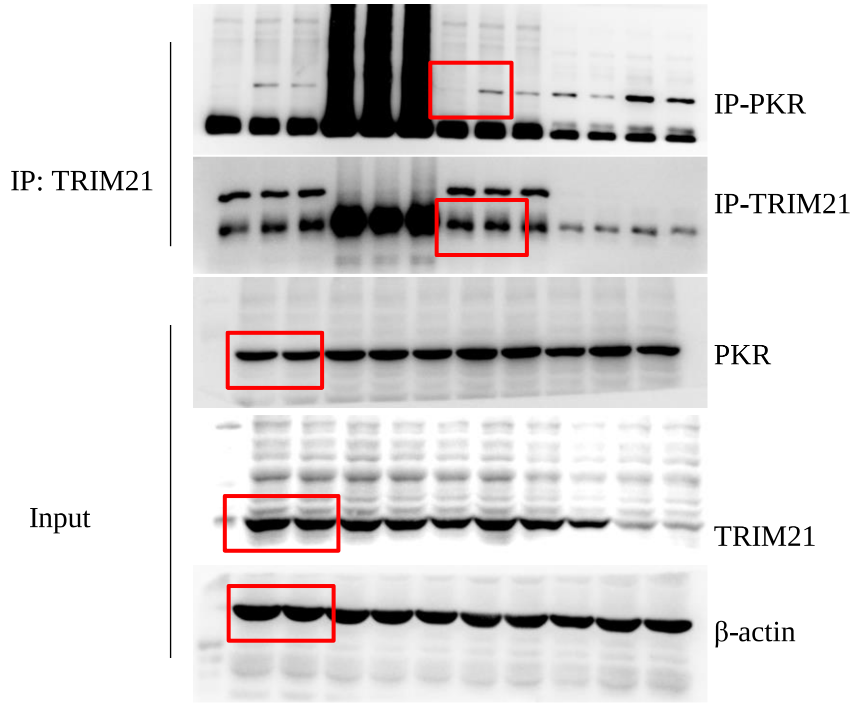
IP-PKR
IP-TRIM21
IP: TRIM21
PKR
Input
TRIM21
β-actin

E

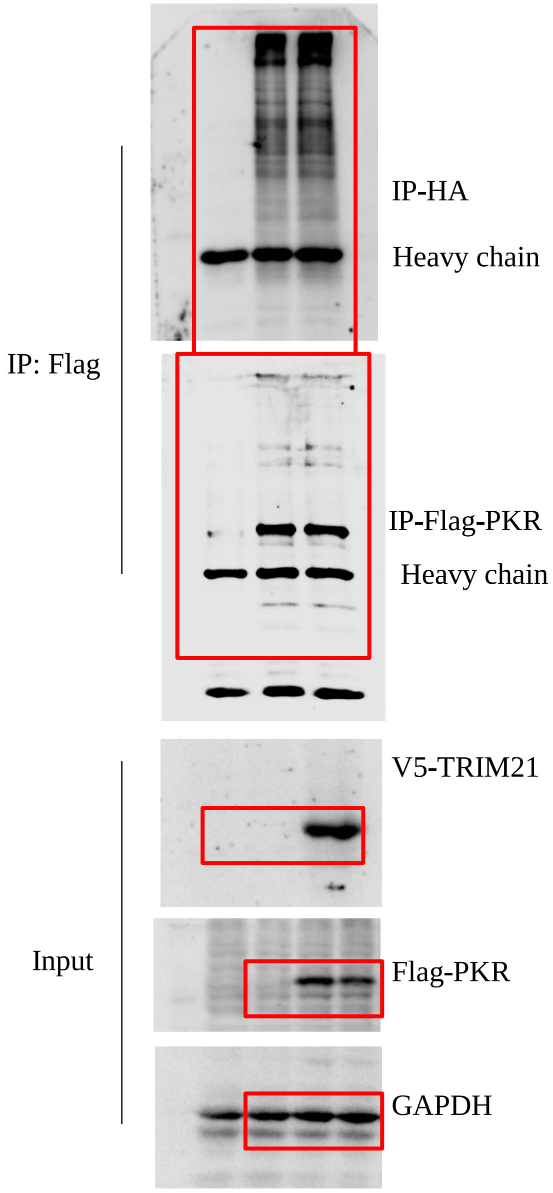
IP-HA
Heavy chain
IP: Flag
IP-Flag-PKR
Heavy chain
V5-TRIM21
Input
Flag-PKR
GAPDH

F

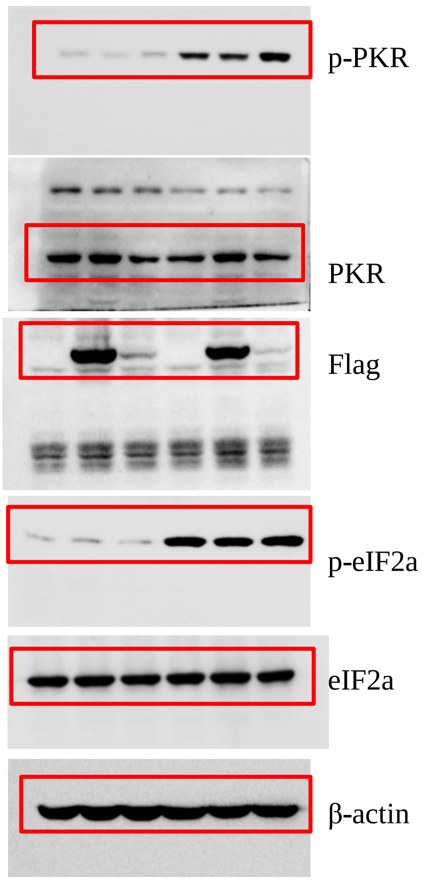
p-PKR
PKR
Flag
p-eIF2a
eIF2a
β-actin

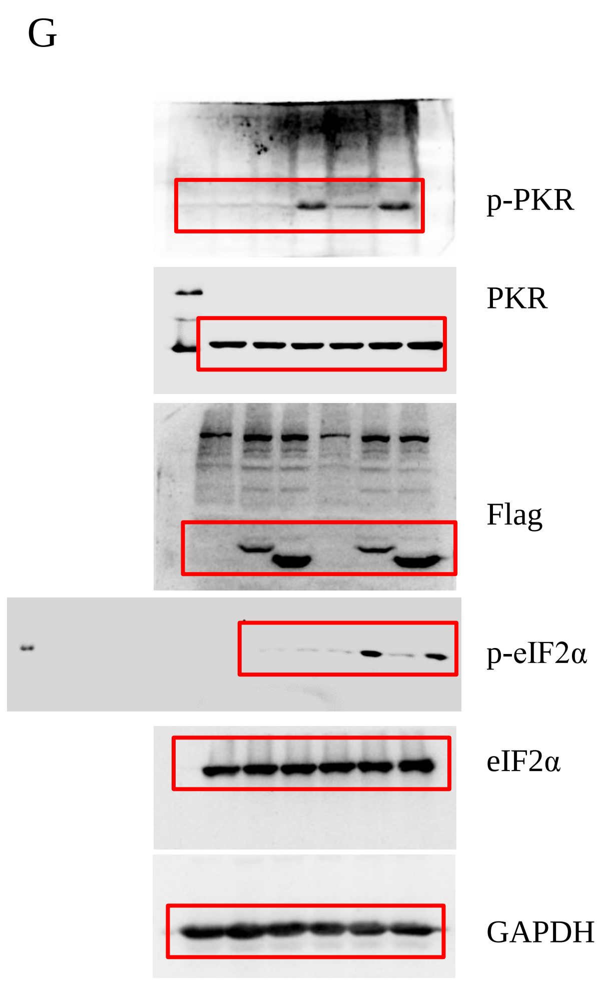
G
p-PKR
PKR
Flag
p-eIF2α
eIF2α
GAPDH

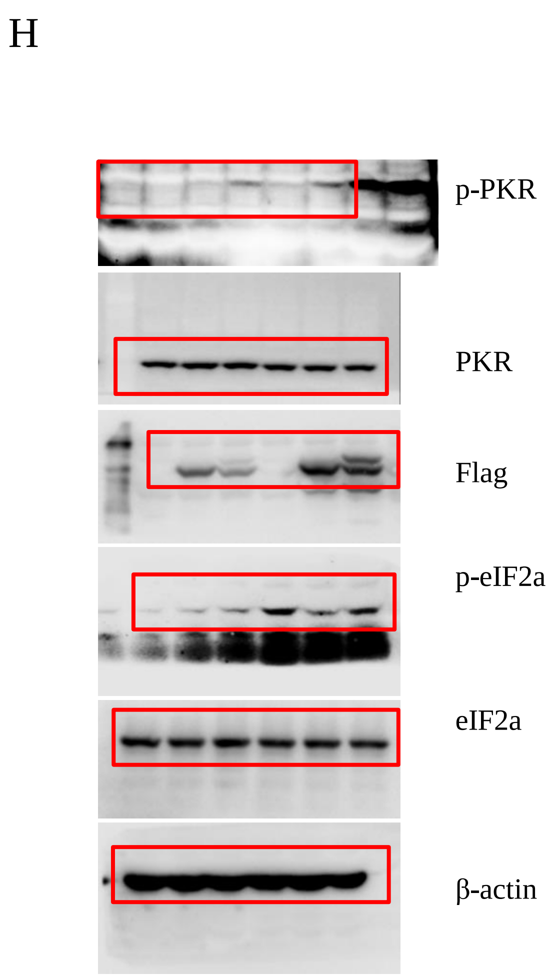
H
p-PKR
PKR
Flag
p-eIF2a
eIF2a
β-actin

Figure 3

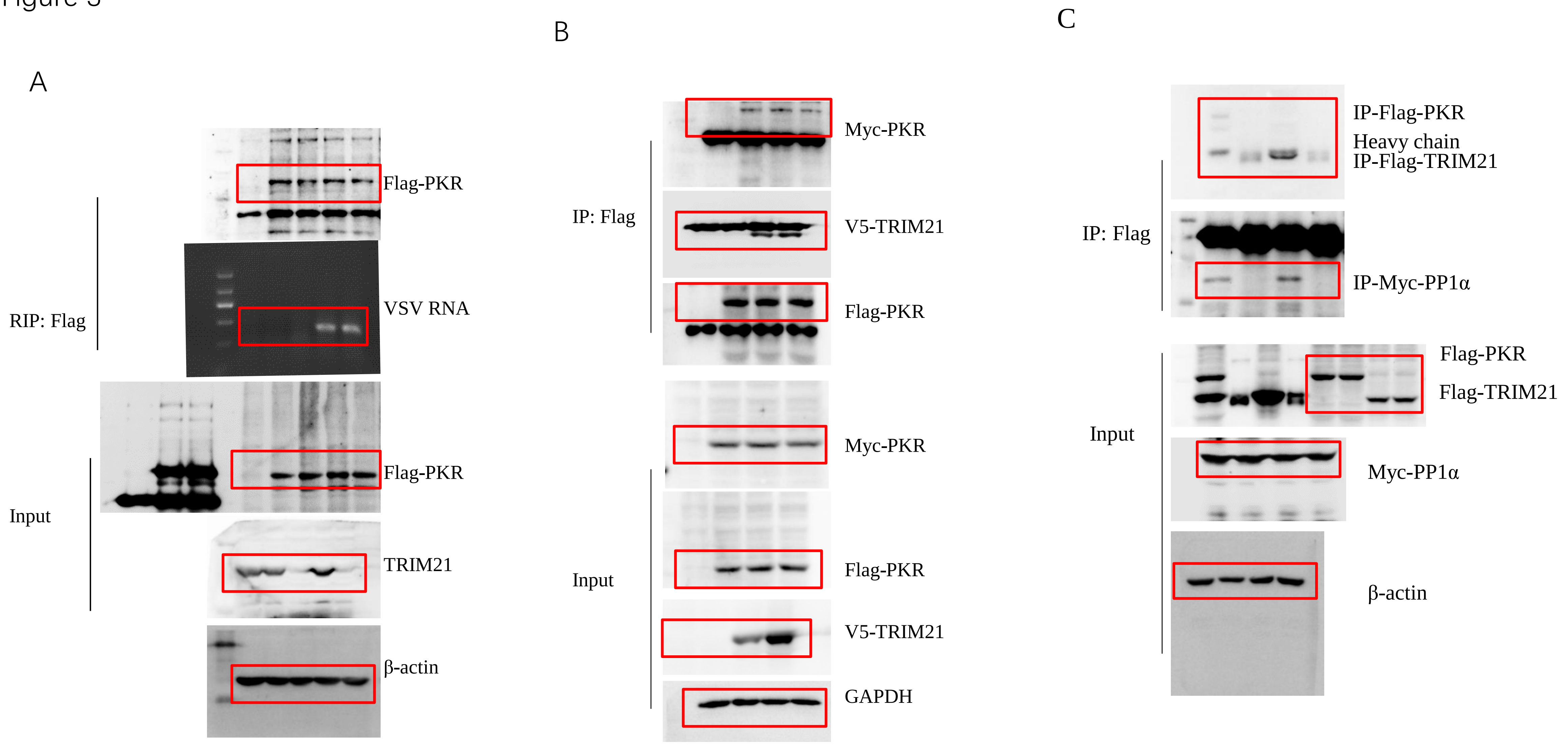
A
Flag-PKR
RIP: Flag
VSV RNA
Flag-PKR
Input
TRIM21
β-actin
B
Myc-PKR
IP: Flag
V5-TRIM21
Flag-PKR
Myc-PKR
Input
Flag-PKR
V5-TRIM21
GAPDH
C
IP-Flag-PKR
Heavy chain
IP-Flag-TRIM21
IP: Flag
IP-Myc-PP1α
Flag-PKR
Flag-TRIM21
Input
Myc-PP1α
β-actin

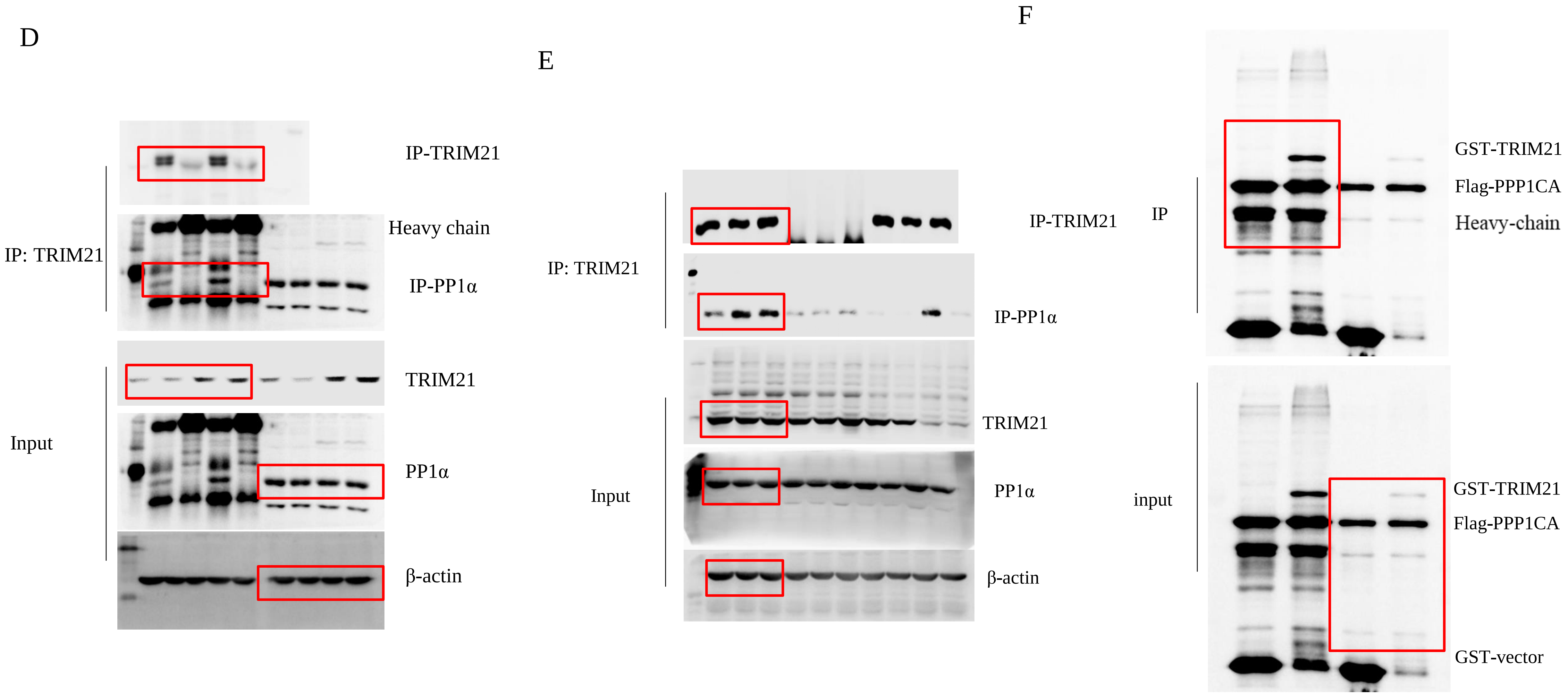
D
IP-TRIM21
Heavy chain
IP: TRIM21
IP-PP1α
TRIM21
Input
PP1α
β-actin
E
IP-TRIM21
IP: TRIM21
IP-PP1α
TRIM21
Input
PP1α
β-actin
F
GST-TRIM21
Flag-PPP1CA
IP
Heavy-chain
GST-TRIM21
input
Flag-PPP1CA
GST-vector

Figure 4

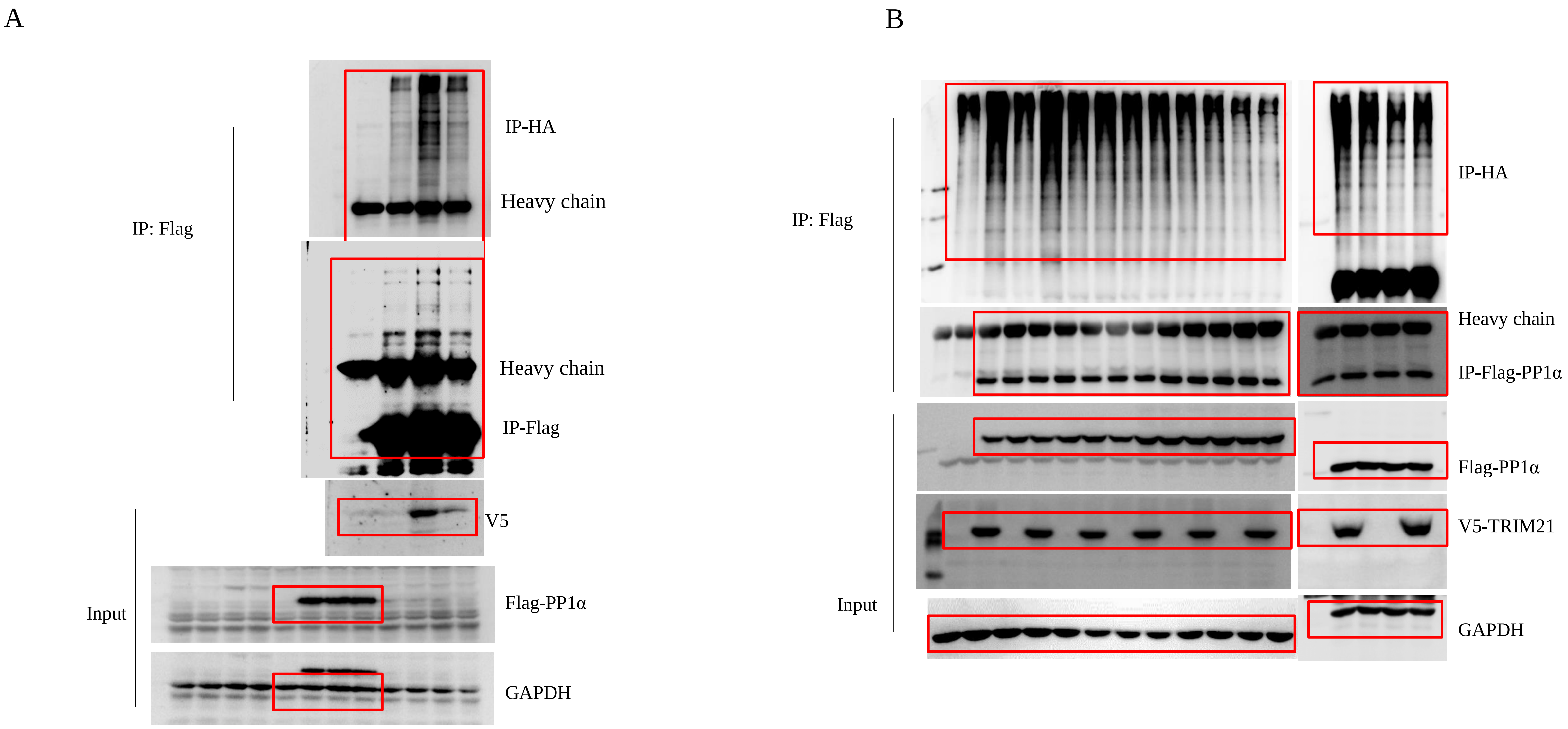
A
IP: Flag
IP-HA
Heavy chain
Heavy chain
IP-Flag
V5
Input
Flag-PP1α
GAPDH
B
IP: Flag
IP-HA
Heavy chain
IP-Flag-PP1α
Input
Flag-PP1α
V5-TRIM21
GAPDH

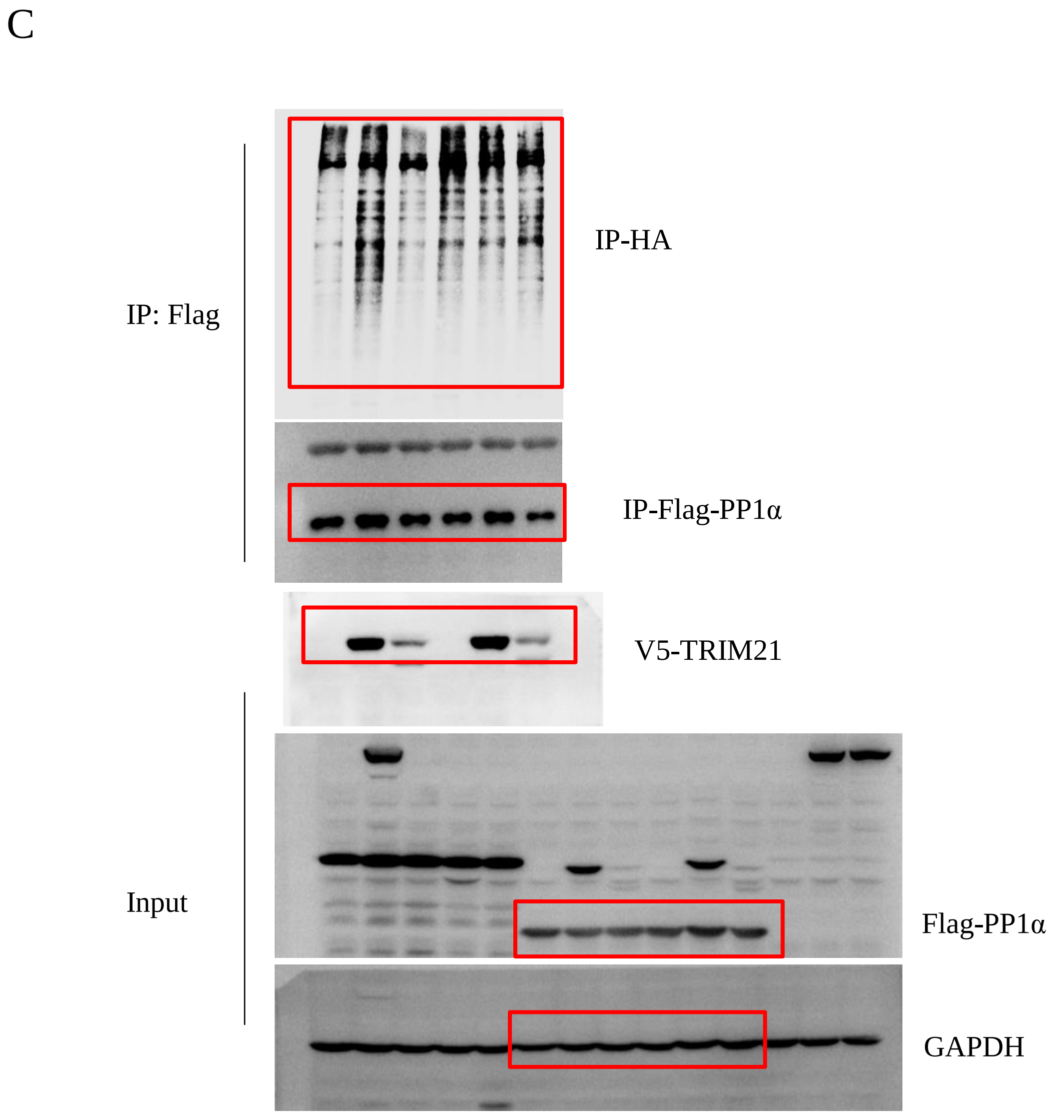
C
IP: Flag
IP-HA
IP-Flag-PP1α
V5-TRIM21
Input
Flag-PP1α
GAPDH

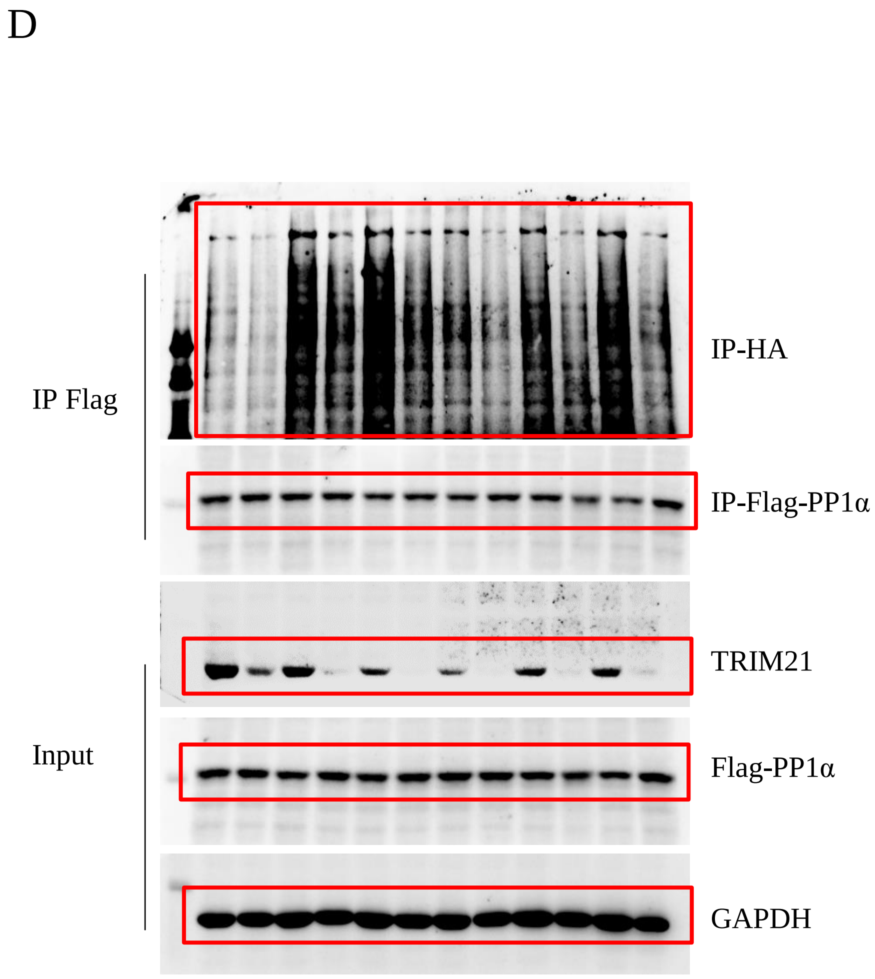
D
IP Flag
IP-HA
IP-Flag-PP1α
TRIM21
Input
Flag-PP1α
GAPDH

F

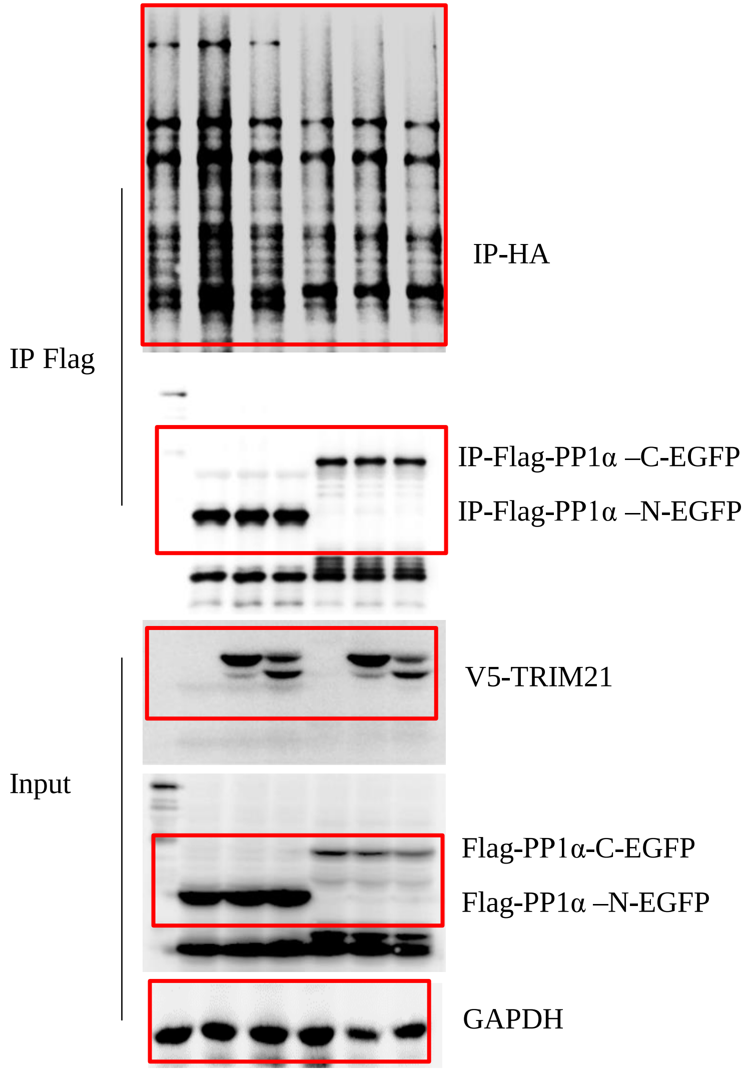
IP-HA
IP Flag
IP-Flag-PP1α –C-EGFP
IP-Flag-PP1α –N-EGFP
V5-TRIM21
Input
Flag-PP1α-C-EGFP
Flag-PP1α –N-EGFP
GAPDH

H

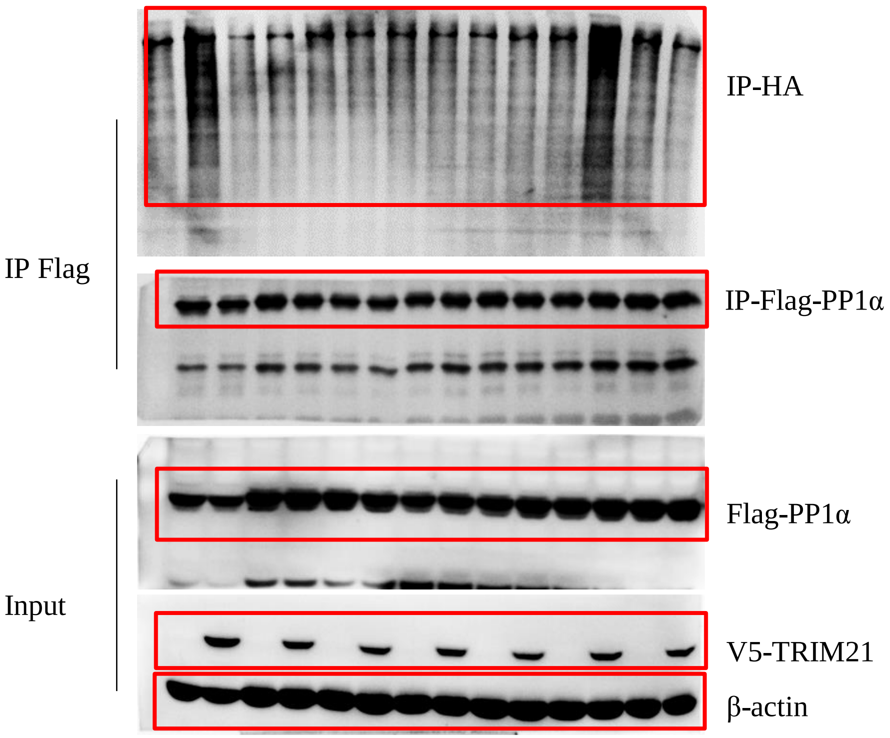
IP-HA
IP Flag
IP-Flag-PP1α
Flag-PP1α
Input
V5-TRIM21
β-actin

I

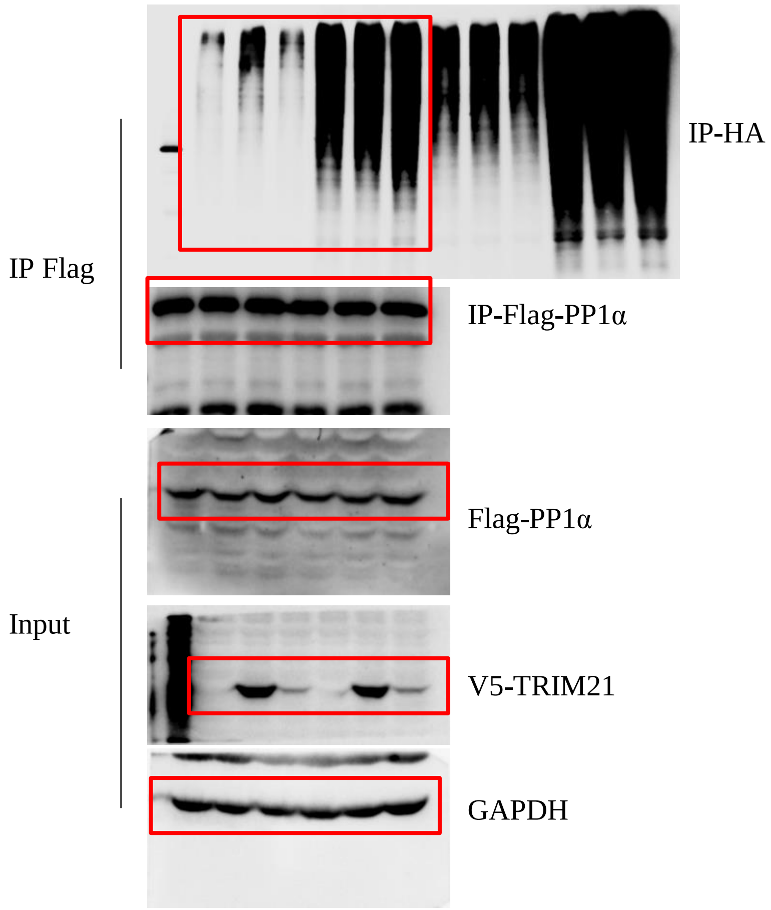
IP-HA
IP Flag
IP-Flag-PP1α
Flag-PP1α
Input
V5-TRIM21
GAPDH

Figure 5

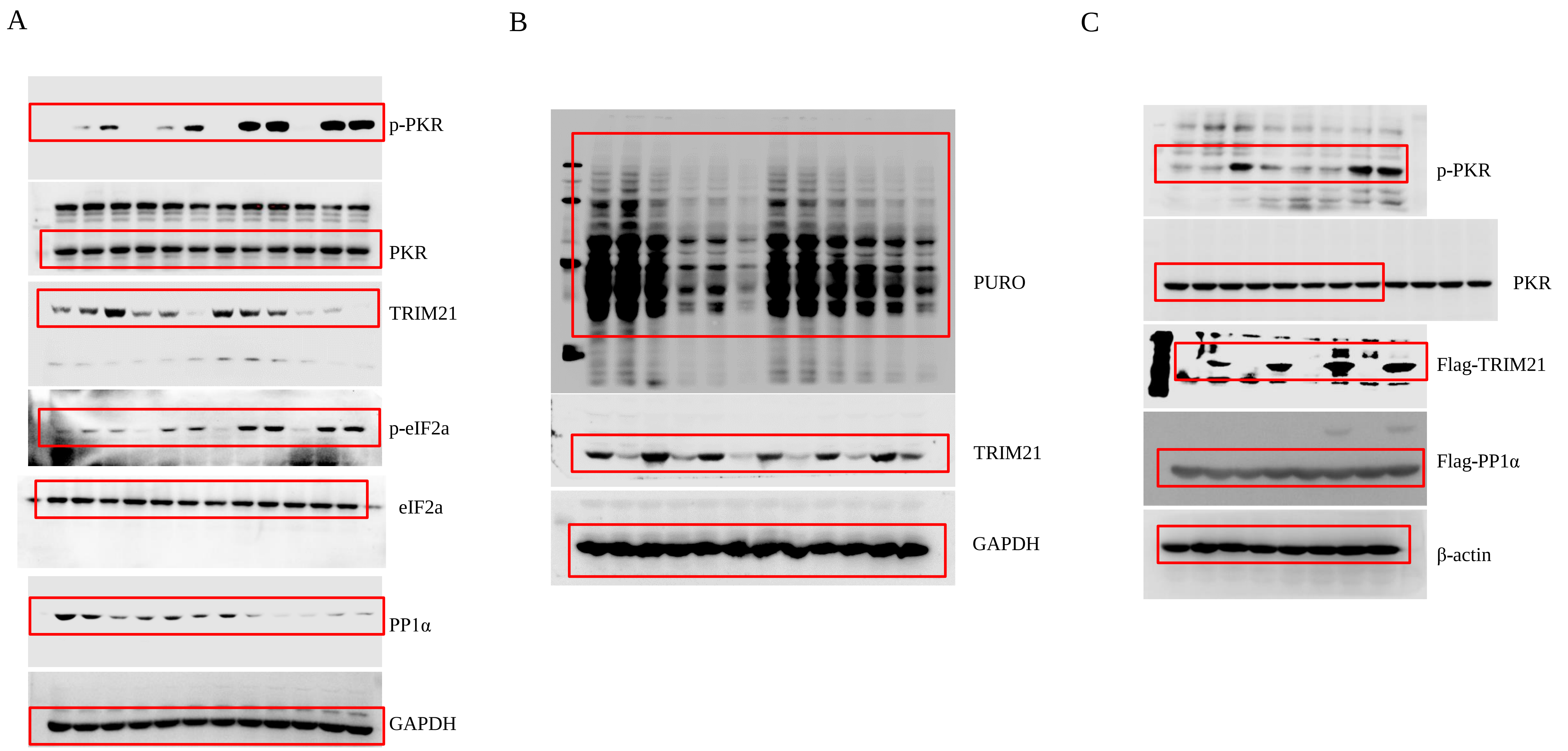
A
p-PKR
PKR
TRIM21
p-eIF2a
eIF2a
PP1α
GAPDH
B
PURO
TRIM21
GAPDH
C
p-PKR
PKR
Flag-TRIM21
Flag-PP1α
β-actin

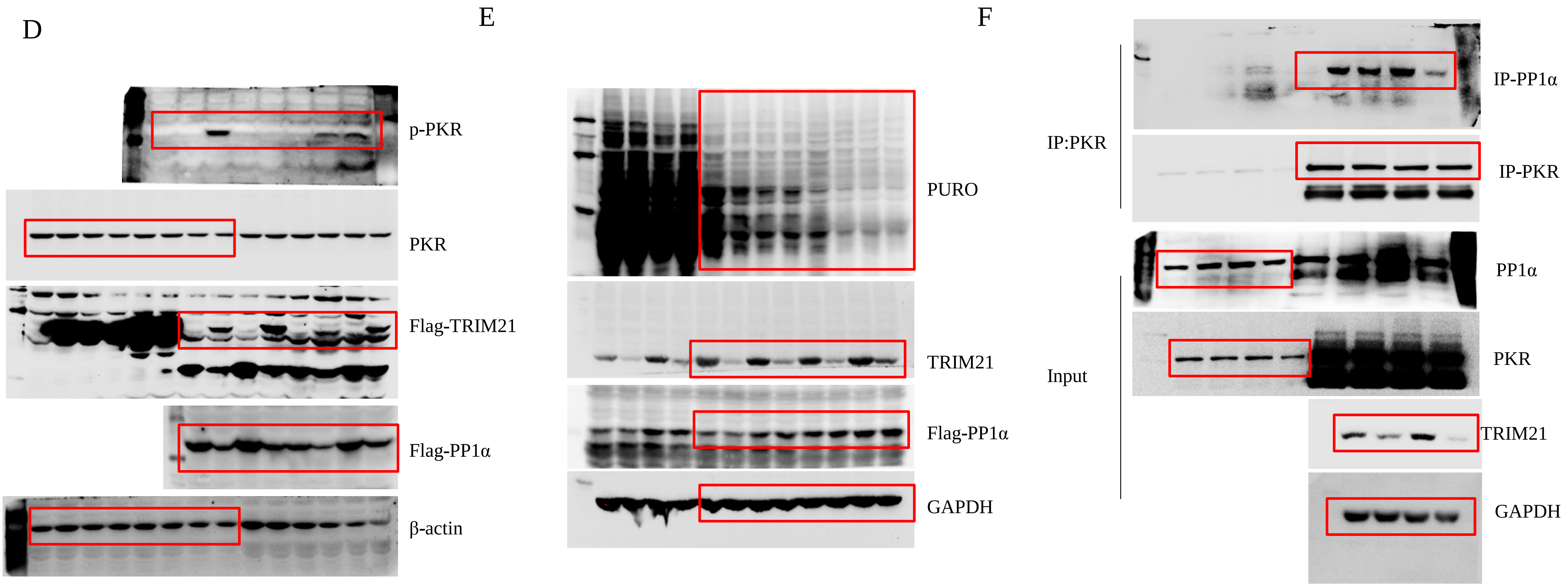
D
p-PKR
PKR
Flag-TRIM21
Flag-PP1α
β-actin
E
PURO
TRIM21
Flag-PP1α
GAPDH
F
IP:PKR
IP-PP1α
IP-PKR
Input
PP1α
PKR
TRIM21
GAPDH

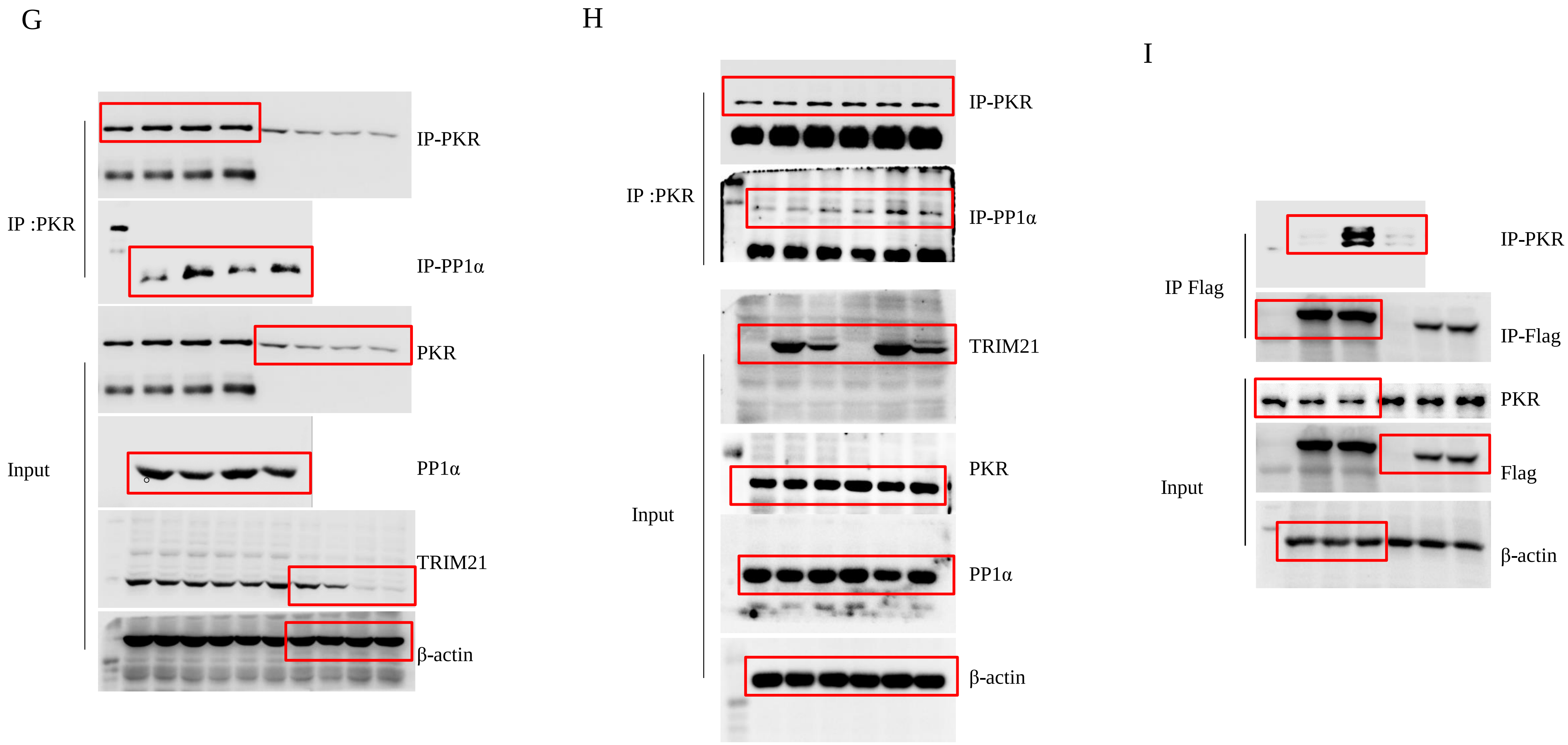
G
IP :PKR
IP-PKR
IP-PP1α
PKR
Input
PP1α
TRIM21
β-actin
H
IP :PKR
IP-PKR
IP-PP1α
TRIM21
Input
PKR
PP1α
β-actin
I
IP Flag
IP-PKR
IP-Flag
Input
PKR
Flag
β-actin

Figure 6

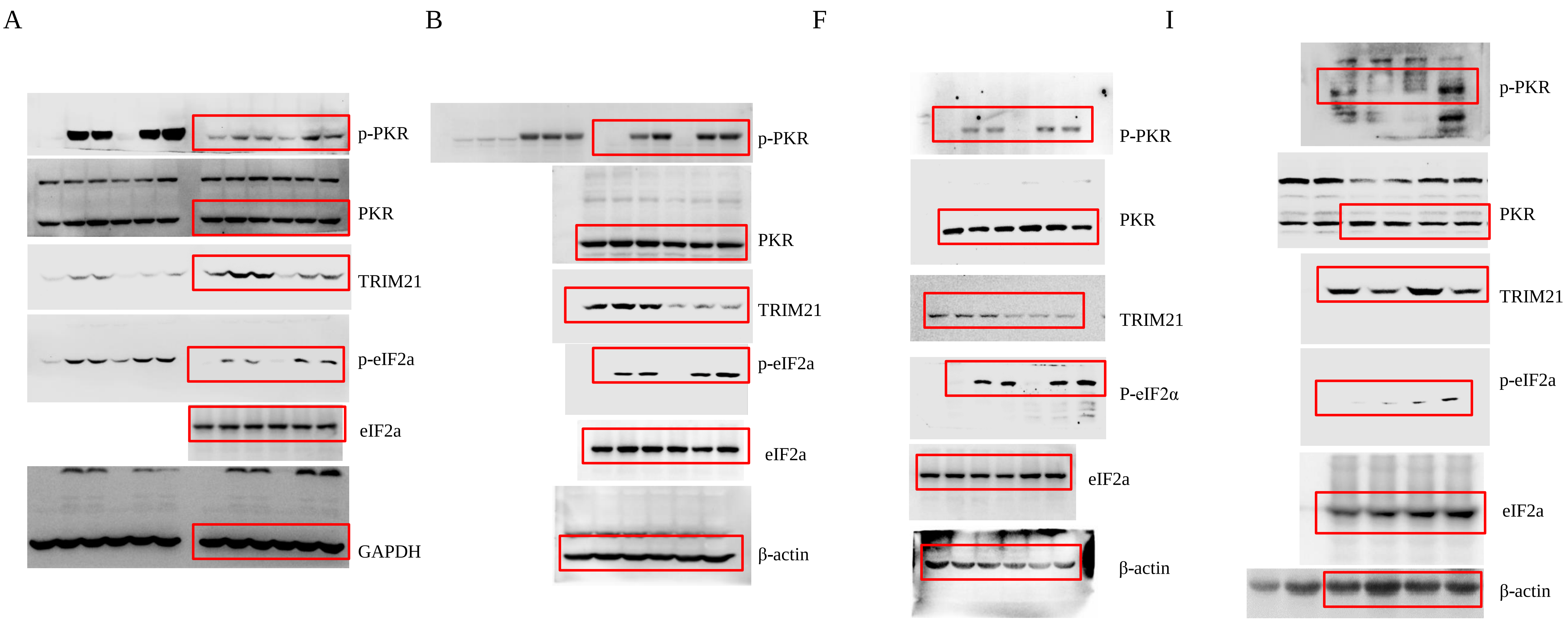
A
B
F
I
p-PKR
PKR
TRIM21
p-eIF2a
eIF2a
GAPDH
p-PKR
PKR
TRIM21
p-eIF2a
eIF2a
β-actin
P-PKR
PKR
TRIM21
P-eIF2α
eIF2a
β-actin
p-PKR
PKR
TRIM21
p-eIF2a
eIF2a
β-actin

Figure 7

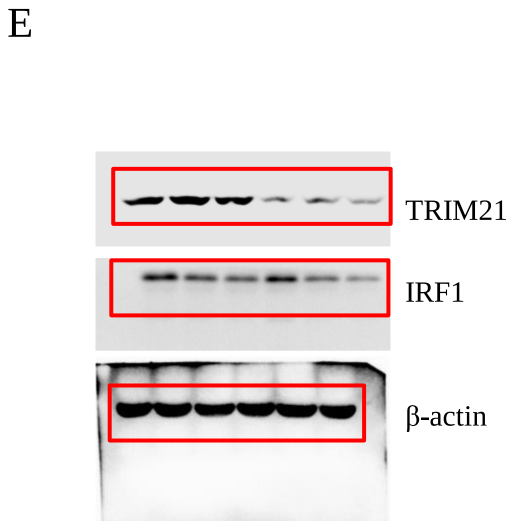

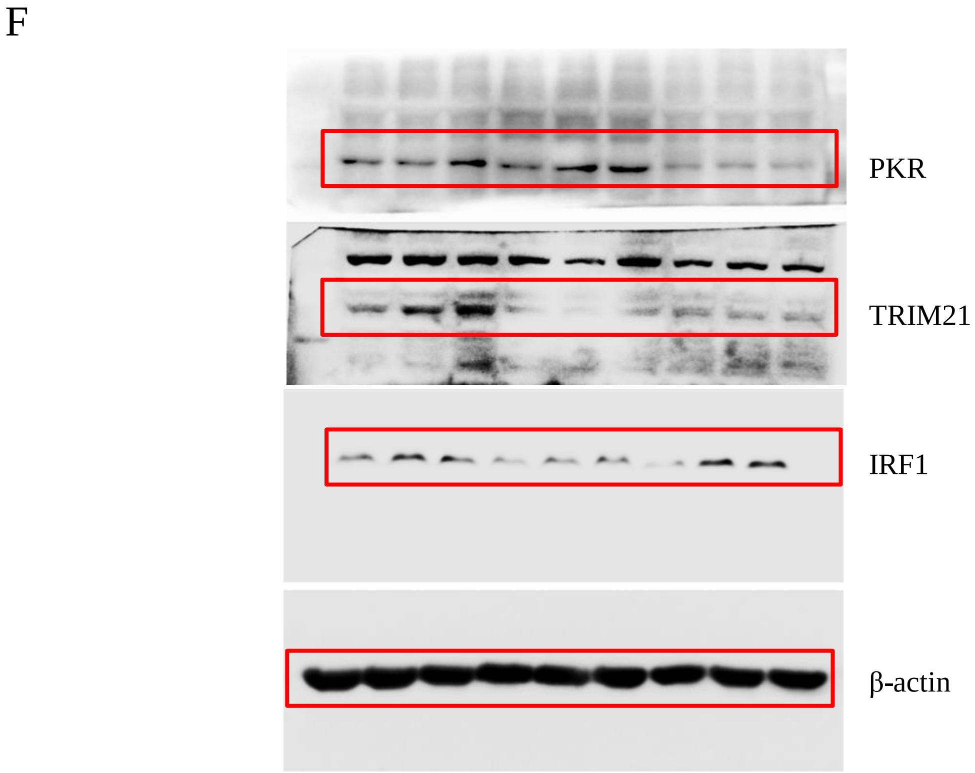

Figure S1

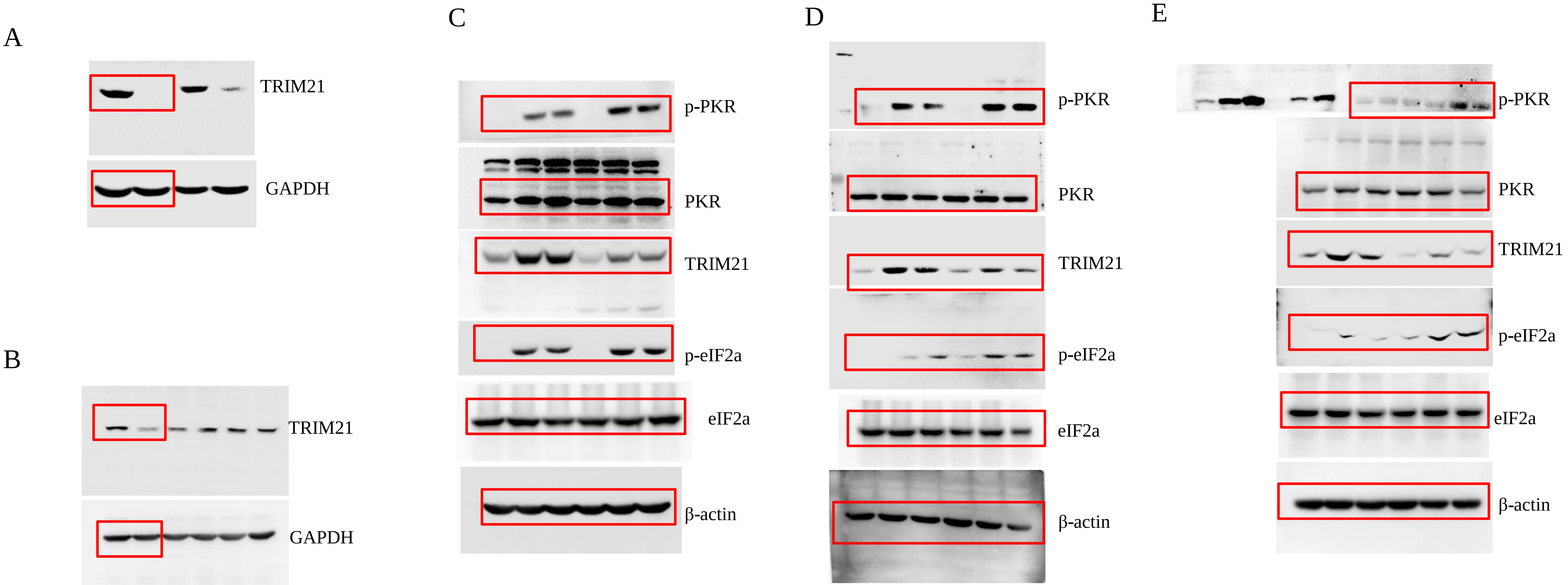
A
TRIM21
GAPDH
B
TRIM21
GAPDH
C
p-PKR
PKR
TRIM21
p-eIF2a
eIF2a
β-actin
D
p-PKR
PKR
TRIM21
p-eIF2a
eIF2a
β-actin
E
p-PKR
PKR
TRIM21
p-eIF2a
eIF2a
β-actin

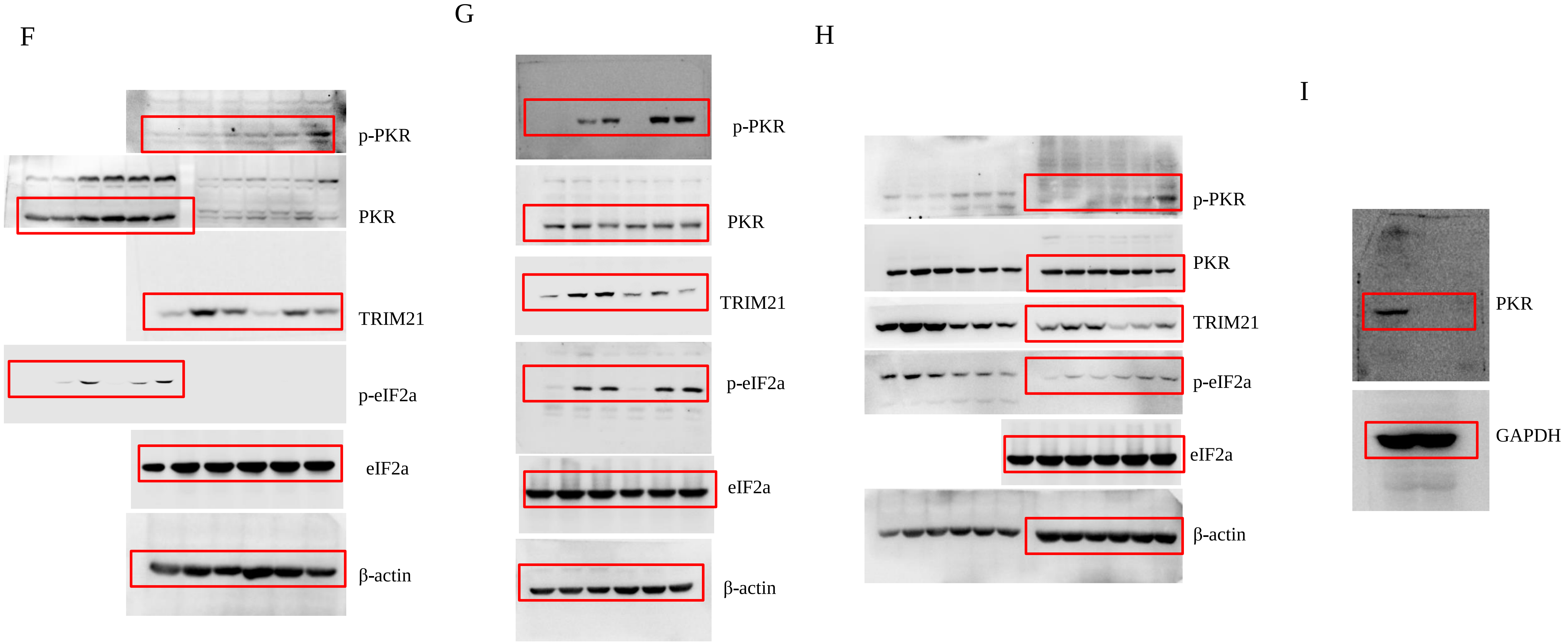
F
p-PKR
PKR
TRIM21
p-eIF2a
eIF2a
β-actin
G
p-PKR
PKR
TRIM21
p-eIF2a
eIF2a
β-actin
H
p-PKR
PKR
TRIM21
p-eIF2a
eIF2a
β-actin
I
PKR
GAPDH

Figure S2

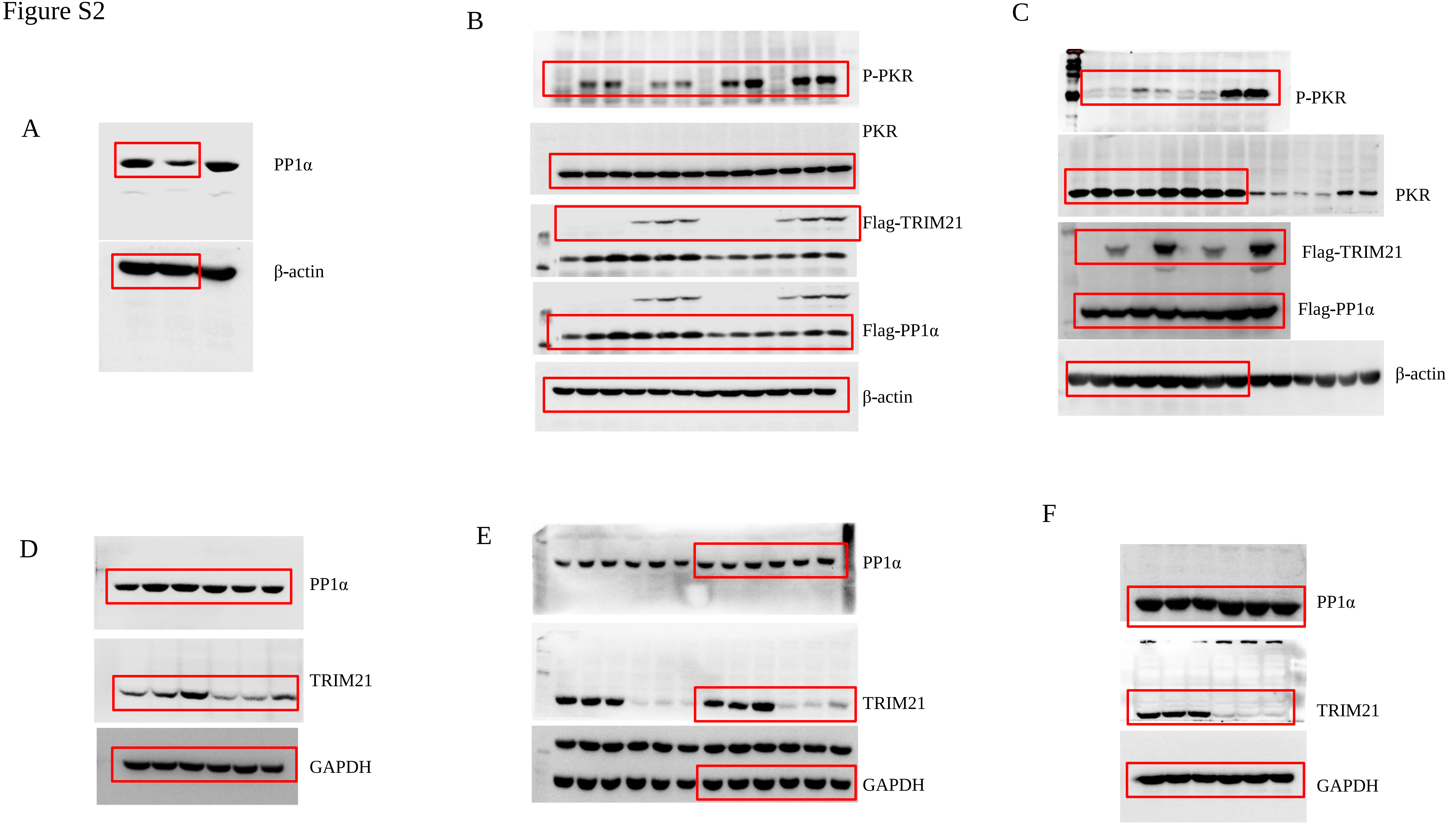

A
PP1α
β-actin
B
P-PKR
PKR
Flag-TRIM21
Flag-PP1α
β-actin
C
P-PKR
PKR
Flag-TRIM21
Flag-PP1α
β-actin
D
PP1α
TRIM21
GAPDH
E
PP1α
TRIM21
GAPDH
F
PP1α
TRIM21
GAPDH

Figure S3

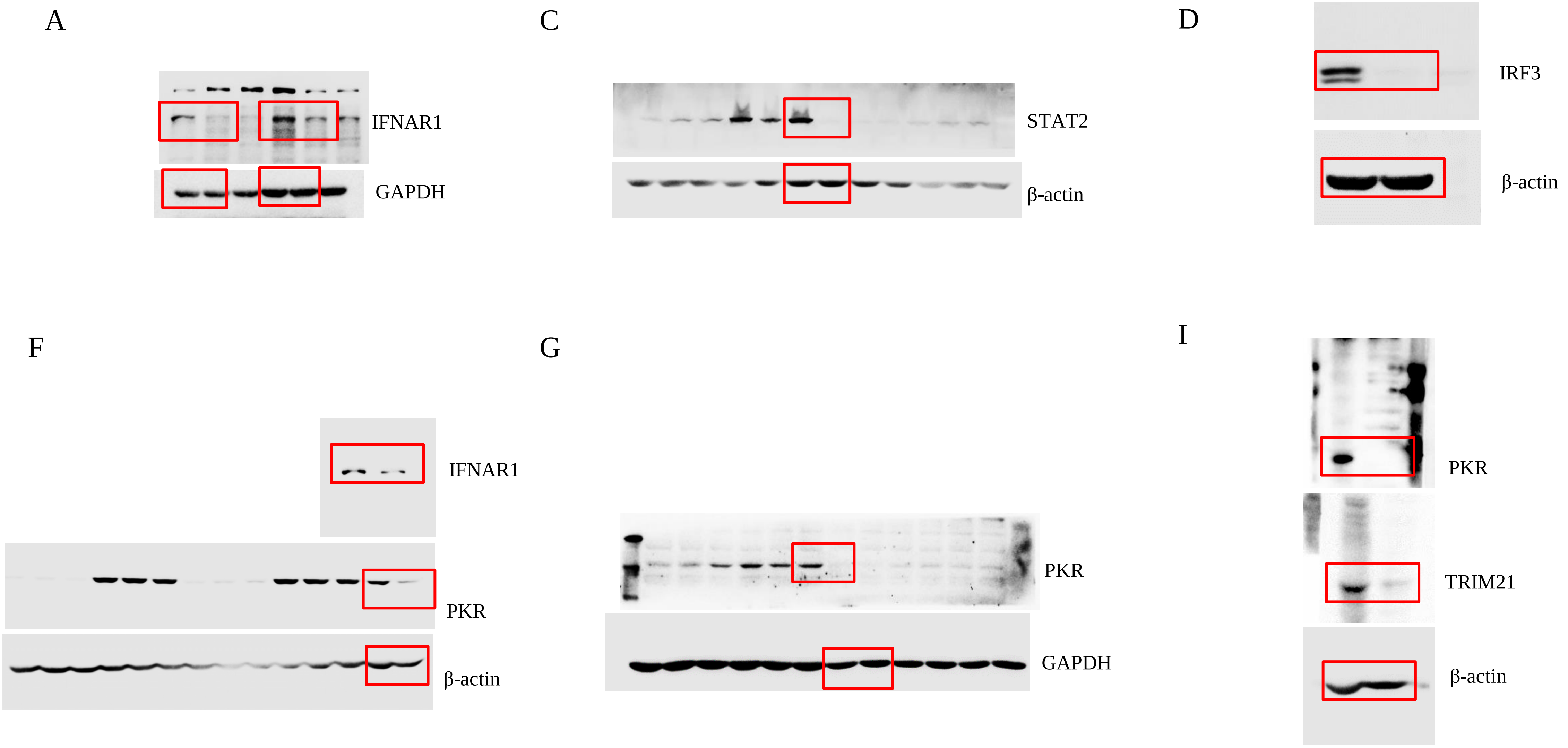
A
IFNAR1
GAPDH
C
STAT2
β-actin
D
IRF3
β-actin
F
IFNAR1
PKR
β-actin
G
PKR
GAPDH
I
PKR
TRIM21
β-actin

Figure S4

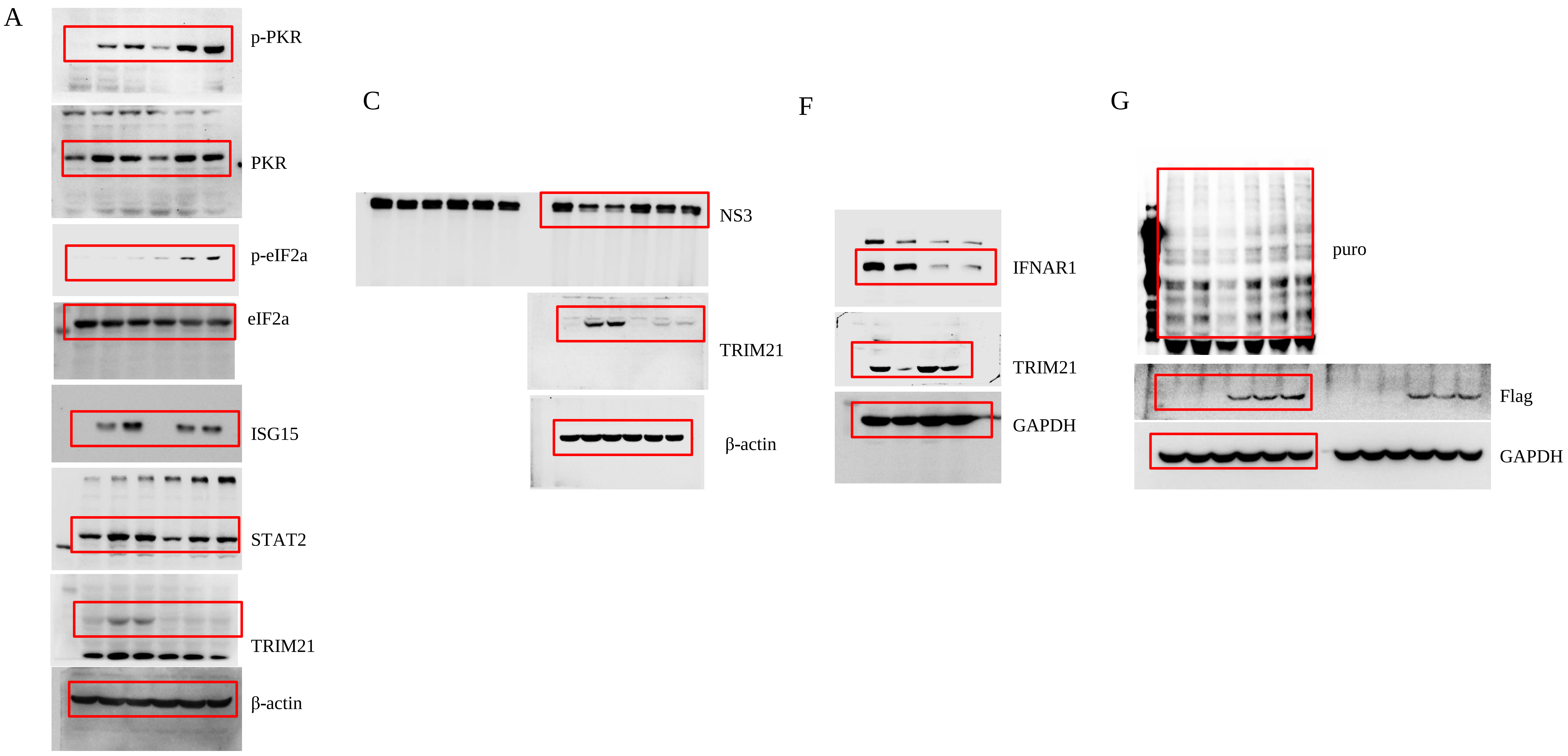
A
p-PKR
PKR
p-eIF2a
eIF2a
ISG15
STAT2
TRIM21
β-actin
C
NS3
TRIM21
β-actin
F
IFNAR1
TRIM21
GAPDH
G
puro
Flag
GAPDH
